# Design, synthesis, biological evaluation, and molecular modeling studies of pyrazole-benzofuran hybrids as new α-glucosidase inhibitor

**DOI:** 10.1038/s41598-021-99899-1

**Published:** 2021-10-21

**Authors:** Fateme Azimi, Homa Azizian, Mohammad Najafi, Ghadamali Khodarahmi, Lotfollah Saghaei, Motahareh Hassanzadeh, Jahan B. Ghasemi, Mohammad Ali Faramarzi, Bagher Larijani, Farshid Hassanzadeh, Mohammad Mahdavi

**Affiliations:** 1 Bioinformatics Research Center, School of Pharmacy and Pharmaceutical Sciences, Isfahan University of Medical Sciences, 81746-73461 Isfahan, Iran; 2 Department of Medicinal Chemistry, School of Pharmacy-International Campus, Iran University of Medical Science, Tehran, Iran; 3 Department of Chemistry, Isfahan University of Technology, Isfahan, 84156-83111 Iran; 4 Department of Medicinal Chemistry, Faculty of Pharmacy and Pharmaceutical Science, Isfahan University of Medical Science, Hezar Jerib, 817416-73461, Isfahan, Iran; 5 School of Chemistry, University College of Science, University of Tehran, P.O. Box 14155-6455, Tehran, Iran; 6 Department of Pharmaceutical Biotechnology, Faculty of Pharmacy, Tehran University of Medical Sciences, P.O. Box 14155-6451, Tehran, 1417614411 Iran; 7 Endocrinology and Metabolism Research Center, Endocrinology and Metabolism Research Institute, Tehran University of Medical Sciences, Tehran, Iran

**Keywords:** Medicinal chemistry, Computational biology and bioinformatics, Drug discovery, Diseases, Medical research, Chemistry, Mathematics and computing

## Abstract

In this work, new derivatives of biphenyl pyrazole-benzofuran hybrids were designed, synthesized and evaluated in vitro through enzymatic assay for inhibitory effect against α-glucosidase activity. Newly identified inhibitors were found to be four to eighteen folds more active with IC_50_ values in the range of 40.6 ± 0.2–164.3 ± 1.8 µM, as compared to the standard drug acarbose (IC_50_ = 750.0 ± 10.0 μM). Limited Structure-activity relationship was established. A kinetic binding study indicated that most active compound **8e** acted as the competitive inhibitors of α-glucosidase with *K*_*i*_ = 38 μM. Molecular docking has also been performed to find the interaction modes responsible for the desired inhibitory activity. As expected, all pharmacophoric features, used in the design of the hybrid, are involved in the interaction with the active site of the enzyme. In addition, molecular dynamic simulations showed compound **8e** oriented vertically into the active site from mouth to the bottom and stabilized the enzyme domains by interacting with the interface of domain A and domain B and the back side of the active site while acarbose formed non-binding interaction with the residue belong to the domain A of the enzyme.

## Introduction

α-glucosidase (EC 3.2.1.20) is a membrane-bound enzyme in the brush border of the intestine which hydrolyses oligosaccharides and polysaccharide to D-glucose, as only monosaccharides can be absorbed from the intestinal lumen^1^. Inhibition of α-glucosidase can retard the release of glucose from complex carbohydrates and therefore can be an important strategy to control hyperglycemia in type-2 diabetes. In addition, the role of cellular α-glucosidase in carbohydrate processing caused that the inhibitors of α-glucosidase also regarded as a convincing therapeutic target for the development of novel drugs for the treatment of numerous diseases, including cancer and viral infections^2–4^. Accordingly, acarbose (Glucobay), miglitol (Glyset), and voglibose (Volix, Basen) are the commercially available *α*-glucosidase inhibitors and are recommended as first-line drugs for the treatment of type-2 diabetes^5^. Due to the sugar-based structure of mentioned α-glucosidase inhibitors, their synthesis needs complicated multistep procedures^6^. Unfortunately, administration of these inhibitors is associated with undesirable side effects, including diarrhea, abdominal discomfort, and flatulence^7,8^. Thus, it would be essential to develop novel, safe, and efficient *α*-glucosidase inhibitors as an effective lead candidate for future antidiabetic drug discovery initiatives^9,10^.

**Figure 1:**
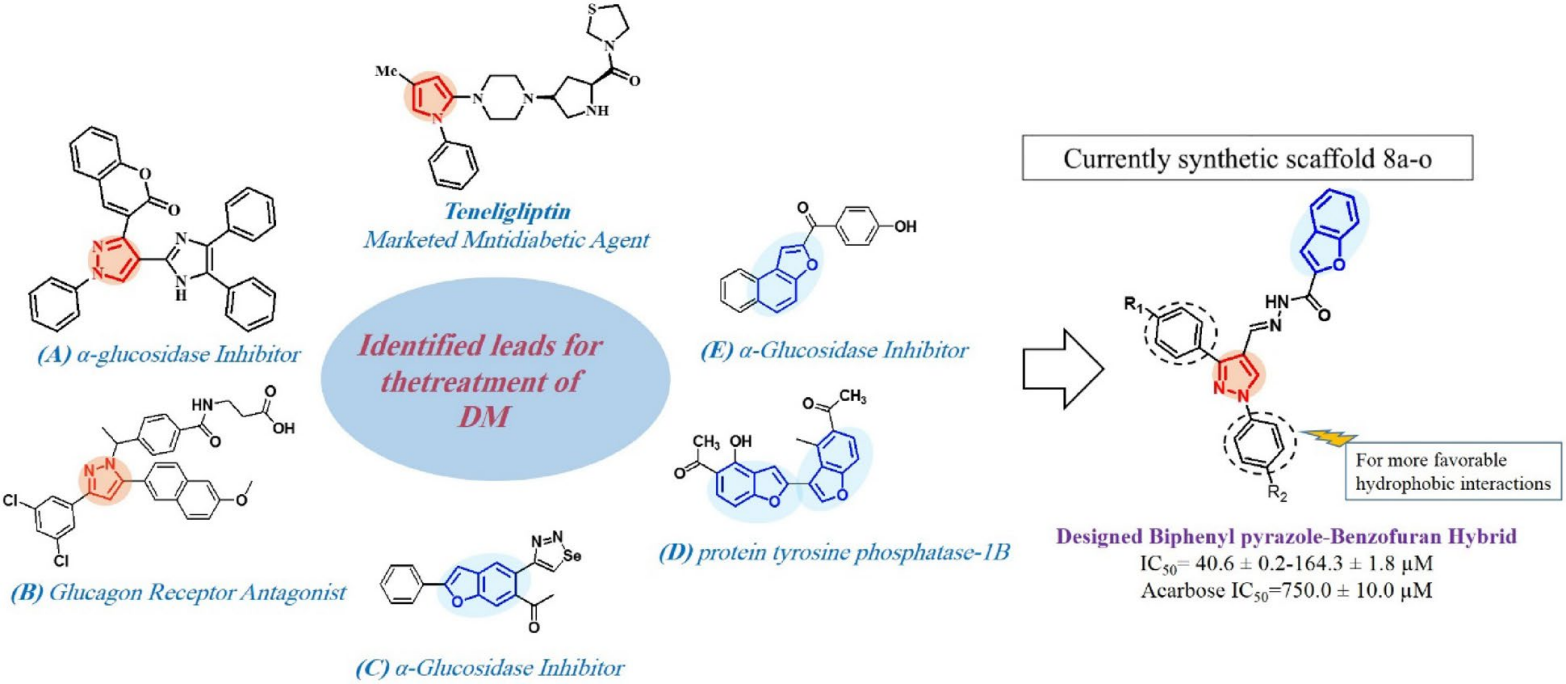
Rationale of the current study.

Pyrazole and its derivatives are one of the most important heterocyclic frameworks in medicinal chemistry, possessing a broad spectrum of pharmacological activities like anti-inflammatory, anti-tubercular, antitumor, antibacterial, anti-Alzheimer’s, antileishmanial, and antihypertensive activities^11^. Over the last years, several well-known drugs that possess pyrazole scaffold like Celecoxib, Viagra, Fipronil, etc., are in clinical use as therapeutic agents^12–14^. Therefore pyrazole nucleus served as a valuable candidate for the exploration of lead molecules. Historically, different derivatives of substituted pyrazole have been introduced as anti hypoglycemic agent^15^. Also recently, several pyrazole-containing agents have been reported as potent antidiabetic^16–19^ and hypoglycemic^20–22^ agents. For instance, Munawar et al. reported a new series of imidazolylpyrazole derivatives as potent α-glucosidase inhibitors (Fig. 1 compound A)^23^ and Xiong et al. discovered the pyrazole-containing derivatives as highly potent and selective glucagon receptor antagonist (Fig. 1 compound B)^24^. It is noteworthy that “Teneligliptin”, antidiabetic drug containing pyrazole, was approved for the treatment of type-II diabetes^25^. In addition to the success of this scaffold in the chemical class of antidiabetic reagents, significant metabolic stability and pharmacological efficiency of pyrazole-based antidiabetic agents encouraged us to further study pyrazole scaffolds to develop a new agent^26^.

On the other hand, interesting physiological and chemotherapeutic properties of benzofuran scaffolds make them promising pharmacophore to design and develop new potentially useful therapeutic agents^27,28^. Moreover, several benzofuran derivatives with α-glucosidase inhibitory activity have been reported. Mphahlele et al. design and synthesis 2-arylbenzofuran-selanadiazole hybrids and evaluated them in vitro through enzymatic assays for inhibitory effect against α-glucosidase (Fig. 1 compound C)^29^. Dimer structures of nature-mimicking hydroxybenzofuran methyl ketones have been evaluated for antidiabetic activity through inhibition of protein tyrosine phosphatase-1B (PTP-1B), which is a legitimate target for the treatment of Type 2 diabetes (Fig. 1 compound D)^30^. Spasov et al. have reported 2-acylbenzofurans as potent α-glucosidase inhibitors (Fig. 1 compound E)^31^.

Over the years, molecular hybridization has been extensively used as the most efficient strategy for the design of novel α-glucosidase inhibitors. It is expected that pharmacophoric hybridization facilitate the development of new compounds with improved affinity and efficacy^32–37^. On the other hand, previous studies have shown that p-stacking and hydrophobic effects play key roles in promoting inhibitory activity of the new compounds against α-glucosidase enzyme^38,39^. Therefore, the pyrazole ring with two attached phenyl groups was incorporated in the design to provide favorable hydrophobic interactions. Hence, prompted by the above observations and in continuation to our attempt in development of α-glucosidase inhibitors, we designed the skeleton of biphenyl pyrazole-benzofuran and evaluated their α-glucosidase inhibition potential^40–44^ (Fig. 1).

Apart from in vitro assessment of target compounds, the mechanism underlying enzymatic inhibition of the most potent compound was further explored using kinetic analysis. Docking studies and molecular dynamic simulation have been performed to determine plausible protein–ligand interactions.

## Results and discussion

### Chemistry

**Figure 2:**
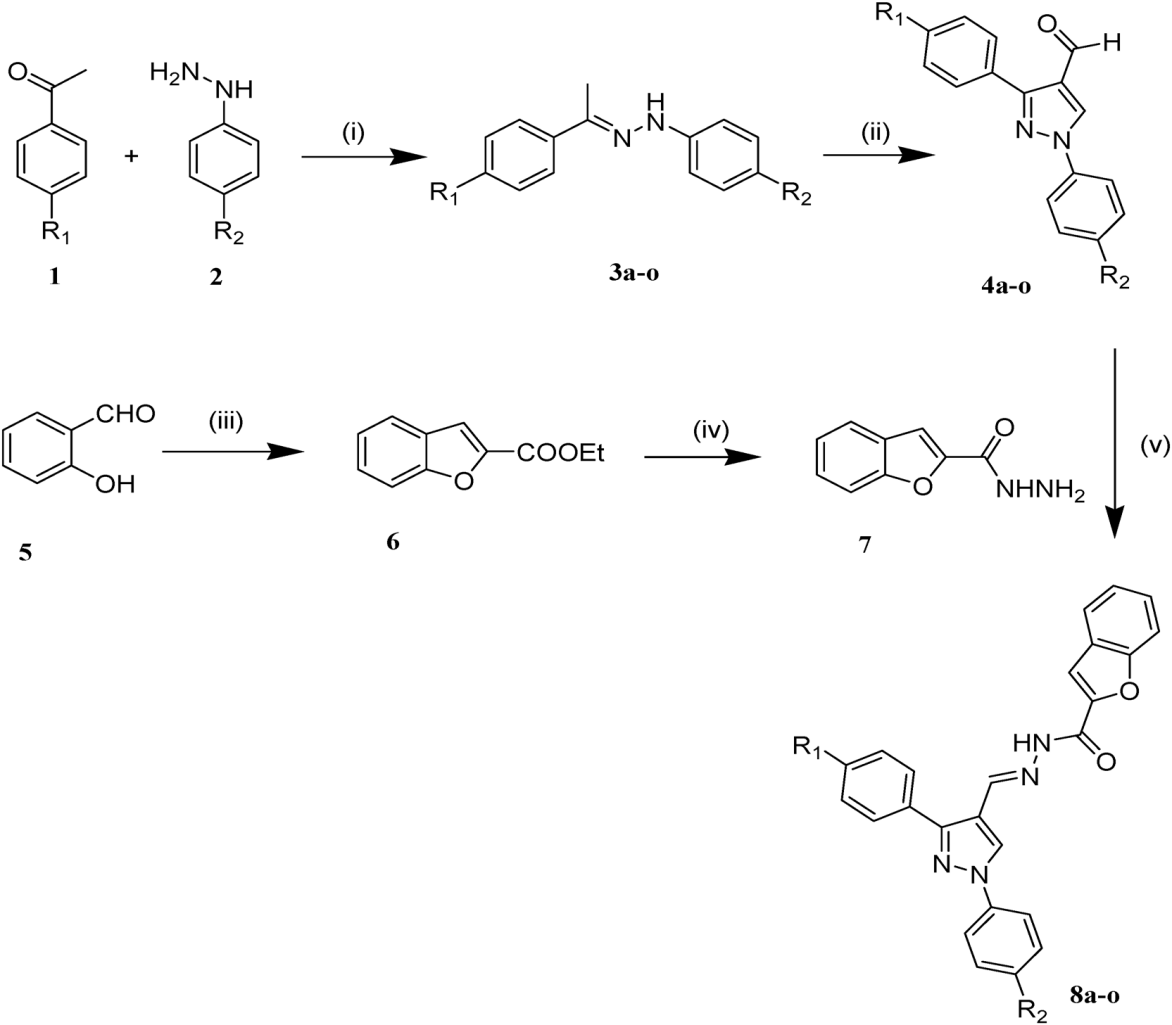
Reagents and conditions for the synthesis of compounds **8a–n**: (i) H_2_SO_4_, EtOH, reflux, 8–12 h, (ii) POCl3, DMF, 60–70 °C, 5–8 h, (iii) Ethyl bromoacetate, K_2_CO_3_, MeCN, reflux, 4 h, (iv) NH_2_NH_2_. H_2_O, EtOH, reflux, overnight, (v) AcOH, EtOH, reflux, 12–18 h.

The synthetic pathway for the preparation of diphenyl pyrazole-benzofuran hybrid **8a–o** is outlined in Fig. 2. The hydrazones **8a–o** were prepared by the condensation reaction of the acetophenone derivatives 1 with the phenylhydrazine or 4-methyl phenylhydrazine hydrochloride 2 under reflux condition in absolute ethanol and in the presence of a catalytic amount of sulfuric acid. Intermediate hydrazone derivatives **3a–o** were converted into 4-formyl pyrazole derivatives **4a–o** by applying Vilsmeier-Haack reaction with POCl_3_–DMF. On the other hand, benzofuran-2-carbohydrazide 7 was prepared by following the reaction scheme. Initially, a mixture of salicylaldehyde 5 with ethyl bromoacetate was refluxed in acetonitrile in the presence of potassium carbonate to afford ethyl benzofuran-2-carboxylate 6. Then, refluxing the ethanolic solution of the ethyl ester 6 and hydrazine hydrate yielded benzofuran-2-carbohydrazide 7. Finally, desired products were obtained by coupling the hydrazide 7 with key aldehydes 4a-o in absolute ethanol and in the presence of glacial acetic acid. The structures of newly synthesized compounds were confirmed by their IR, ^1^H NMR, ^13^C NMR, MASS and elemental analysis^29,45^.

### In vitro α-glucosidase inhibitory activity

All of the newly synthesized compounds **8a–o** were screened for α-glucosidase inhibition activity. The α-glucosidase enzyme (from Saccharomyces cerevisiae, EC3.2.1.20) was used to evaluate the α-glucosidase inhibitory activity. Compared to human α-glucosidase, they shared similarities in substrate specificity, pH optimum, catalytic residues in the active site and inhibitor sensitivity^46^. Therefore, α-glucosidase from yeast is extensively used for the preliminary screening of α-glucosidase inhibitors^47,48^. Acarbose, a commercially available α-glucosidase inhibitor, was used as the reference drug and the results are expressed as IC_50_ values (Table 1). Albeit, all pharmacophoric groups in the designed hybrid seem to have involved in the inhibitory potential but to further elucidate the role of substituents on the aryl rings connected to pyrazole, a wide variety of compounds **8** were synthesized and structure–activity relationship (SAR) has been established based on varying substituents in R_1_ and R_2_. Accordingly, SAR was investigated in two categories of **8a–i** (Table 1, R2 = H) and **8j–o** (Table 1, R2  = CH_3_). It is worth mentioning that all synthetic compounds showed significant activity with IC_50_ values in the range of 40.6 ± 0.2–164.3 ± 1.8 µM, when compared to acarbose (750.0 ± 10.0 µM). Among them, compound **8e** (IC_50_ = 40.6 ± 0.2 μM), having a nitro group in the R_1_ position, was found to be the most potent inhibitor. This is approximately 18-fold more potent than the standard acarbose. The introduction of other electron-withdrawing groups, such as halogen atoms and trifluoromethyl (**8f–i**), led to reduced inhibitory activity. Halogenated counterparts of compound **8e** in the first category of compounds, exhibited inhibitory activity generally depending on the size of halogen substitution. Compound **8f** (IC_50_ = 79.7 ± 0.5 μM) bearing bromo substituent was found to be the second most active analog in this category and nine-fold more potent than standard. Compounds **8g** having a relatively large chlorine substituent and **8h** having a small fluorine group showed lower activities with IC_50_ values of 125.3 ± 1.0 and 164.3 ± 1.8 µM, respectively. It should be noted that change the fluoro group with trifluoromethyl moiety, as in the case of compound **8i** (IC_50_ = 101.7 ± 0.7 μM), led to a significant improvement in the inhibition potential. Finally, the order of inhibition for compounds **8a–d** was **8c** > **8b** > **8d** > **8a** (IC_50s_ = 100.3 ± 0.7, 108.3 ± 0.8, 133.6 ± 1.2, and 141.2 ± 1.4 μM, respectively), which illustrated that inhibitory activity almost affected by the electron-donating property of substituent.

**Table 1:** In vitro α-glucosidase inhibitory activity of compounds 8a-p.


Compd	R_1_	R_2_	IC_50_(µM)^a^	Compd	R_1_	R_2_	IC_50_(µM)^a^
**8a**	H	H	141.2 ± 1.4	8j	H	CH_3_	157.1 ± 1.6
**8b**	CH_3_	H	108.3 ± 0.8	8 k	CH_3_	CH_3_	115.6 ± 0.9
**8c**	OCH_3_	H	100.3 ± 0.7	8 l	OCH_3_	CH_3_	54.7 ± 0.3
**8d**	OH	H	133.6 ± 1.2	8 m	OH	CH_3_	127.0 ± 1.0
**8e**	NO_2_	H	40.6 ± 0.2	8n	Br	CH_3_	95.6 ± 0.6
**8f**	Br	H	79.7 ± 0.5	8o	Cl	CH_3_	154.7 ± 1.6
**8g**	Cl	H	125.3 ± 1.0	Acarbose	–	–	750.0 ± 10.0
**8h**	F	H	164.3 ± 1.8				
**8i**	CF_3_	H	101.7 ± 0.7				

^a^Values are the mean ± SD. All experiments were performed at least three times.

For further investigation the SAR, second category of compounds **8j–o** possessing methyl group in R_2_ were also examined. Compound **8l** (IC_50_ = 54.7 ± 0.3 μM) containing methoxy group was found to be the most active compound in this series. Also, this compound is the second most potent among the screened compounds and > 13 folds more active than the standard. Replacement of methoxy group in **8l** with methyl (**8k**, IC_50_ = 115.6 ± 0.9 μM) or hydroxyl group (**8m**, IC_50_ = 127.0 ± 1.0 μM) resulted in a remarkable decrease the biological activity. The bromo substituted derivative **8n** was found as the second most potent molecule among this series of compounds and exhibited approximately eight-fold enhanced activity compared to the standard acarbose. So, as mentioned in the previous sections, the size of the bromine may be responsible for the higher activity of brominated compounds than other halogens.

It can be concluded that the in vitro α-glucosidase inhibitory activity mainly depends upon the substituents on R_1_ and the nature of substituents at this position affected the efficacy of the methyl group on R_2_. In this regard, the α-glucosidase inhibitory activity of bromine (**8f**, **8n**) and chlorine (**8g**, 8o) substituted analogs in two series of mentioned compounds revealed that the introduction of methyl groups on R_2_ led to lower activity (**8f** > **8n** and **8g** > **8o**). Similarly, the lack of methyl group in the case of compound **8k** resulted in a slight decreased in inhibitory activity as compound **8b**. In a different manner, compound **8l** was more active than compound **8c**. Both compounds have a methoxy group at the R_1_ position, but the presence of the methyl group on R_2_ in compound **8l** led to a two-fold increase in activity. The same is the case of compounds **8d** and **8m** having hydroxyl group, the methyl substituent on R_2_ position was found to confer an increase in inhibitory activity.

### Kinetic study

**Figure 3:**
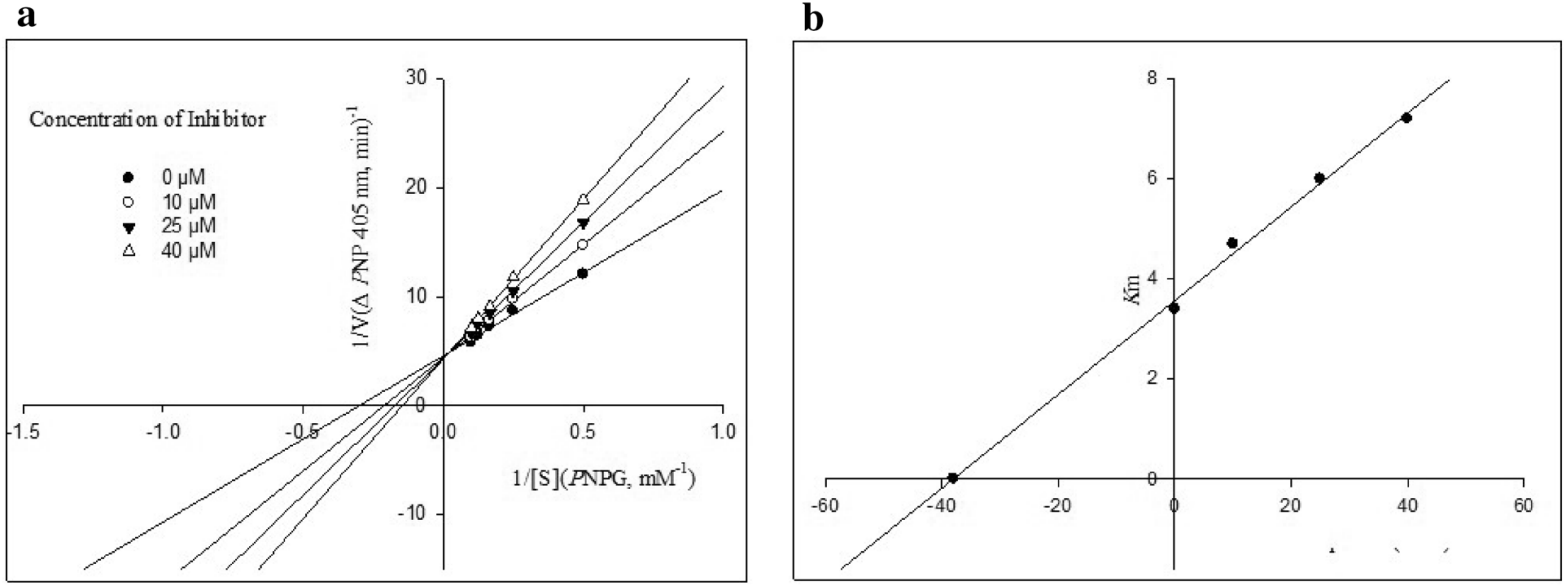
(**a**) Lineweaver–Burk plots for the inhibition of α-glucosidase by compound **8e**. (**b**) The secondary plot between K_m_ and various concentrations of compound **8e**.

To gain further insight into the mechanism of action of the synthesized compounds against α-glucosidase, a kinetics analysis was performed on the most potent compound **8e**. In different concentrations of test compound (0, 10, 25, and 40 µM) and with the incremental concentration (2–10 mM) of substrate, the rate of the enzyme activity was calculated. The type of inhibition and experimental inhibition constant (*K*_*i*_) value were determined by employing Lineweaver–Burk plots and secondary re-plot of these plots, respectively. The Lineweaver–Burk plot showed that with varying concentrations of compound **8e**, V_max_ of enzyme gradually increased without affecting the K_m_ of enzyme (Fig. 3a). This pattern indicates a competitive type of inhibition. The plot of the slope of lines in the Lineweaver–Burk plots (k_m_) against the inhibitor concentration gave an approximation of the inhibition constant, *K*_*i*_ of 38 μM for compounds **8e** (Fig. 3b).

### Cytotoxic activity

Cytotoxicity of the most potent compounds **8e**, **8l**, **8f** and **8n** were evaluated against normal 3T3 cell line by using MTT assay. The results of cytotoxic activity were expressed as the IC_50_ (µM) and outlined in Table 2. Results revealed that at 150 µM concentration, the selected compounds were noncytotoxic against studied normal cell line.

**Table 2:** Cytotoxicity of the most potent compounds **8e**, **8l**, **8f** and **8n** against 3T3 cell line.

Compounds	Cytotoxicity (3T3 cell line) IC_50_ (µM)^a^
**8e**	> 150
**8l**	> 150
**8f**	> 150
**8n**	> 150

^a^All experiments were performed at least three times.

### Homology modeling and molecular docking study

**Figure 4:**
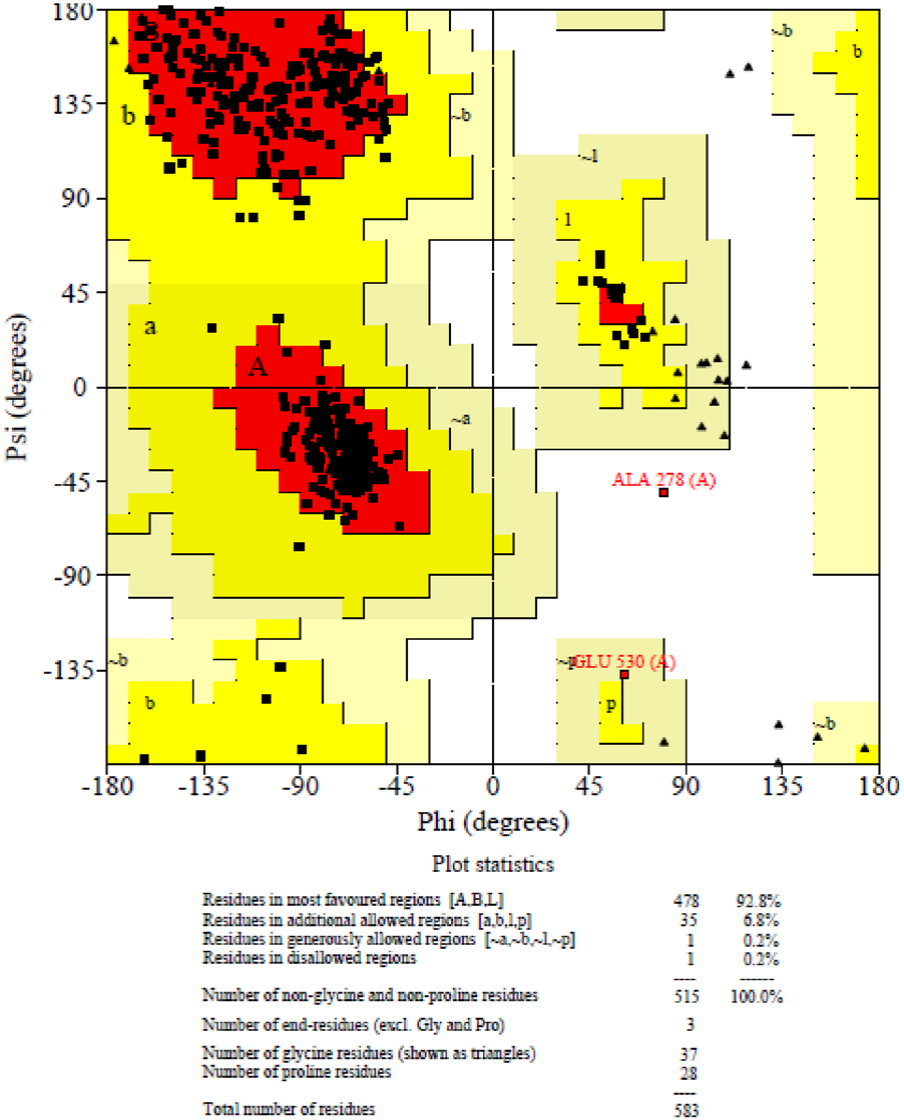
Ramachandran plot of the modelled a-glucosidase enzyme.

Molecular docking studies were also performed to rationalize the results of biological assays and gain structural insight into the binding of the synthetic derivatives against *α*-glucosidase. Due to the unavailability of the crystallographic structure of α-glucosidase from *S. cerevisiae*, the 3D structure of α-glucosidase was modeled using MODELER inbuilt in Discovery Studio (DS) package and synthetic compounds were docked against the established homology model. For this purpose, the FASTA format of the primary sequence was downloaded from Uniprot (Access code P53341) and submitted to NCBI BLAST to get a template with a suitable identity for sequence alignment^49^. Isomaltase from S. cerevisiae (PDB ID: 3A4A) with 71.4% identity and 86.7% similarity was selected as the template for modeling^50^. The best model was selected based on the lowest PDF Total Energy (3270.6404) and DOPE Scores (− 73110.257813) and evaluated for further validation. The PROCHECK program was applied to assess the stereochemical quality of the model. The phi/psi Ramachandran plot distributions indicated that 99.6% residues are in the favored and allowed regions and only 0.2% residues lie in the outlier region (Fig. 4).

**Figure 5:**
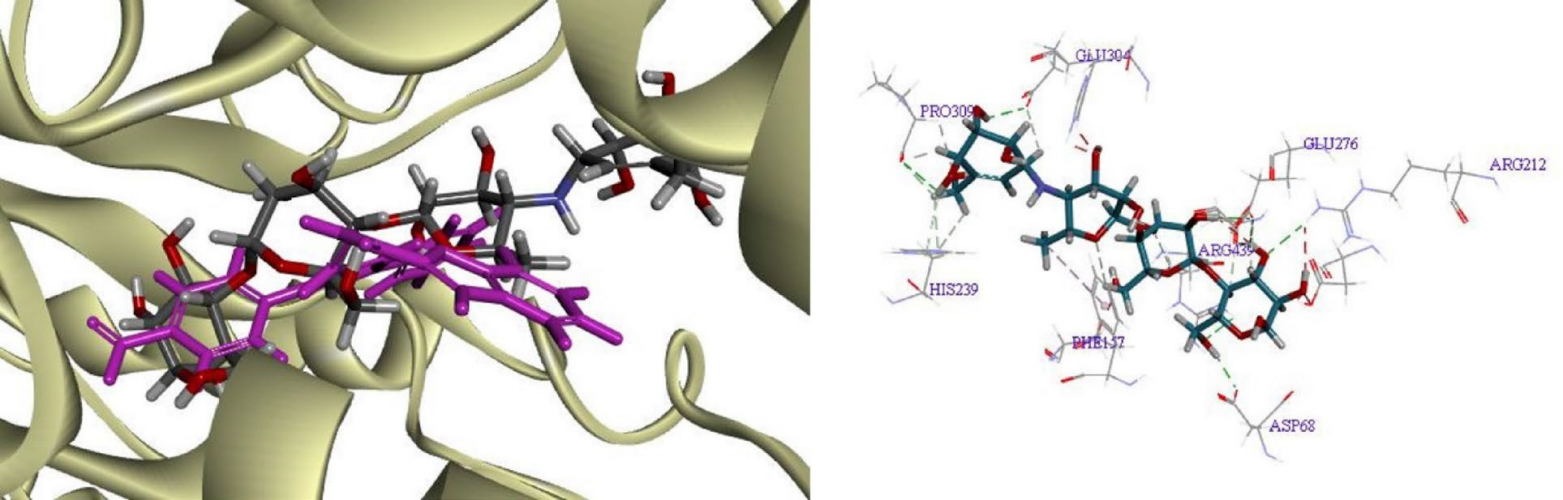
Acarbose (gray) and the most potent compounds **8e** (pink) superimposed in the active site pocket of modeled α-glucosidase.

The superposed structure of acarbose (standard inhibitor) and the predicted top-scored conformation of the most potent compound **8e** in the active site of a homology model of *α*-glucosidase was shown in Fig. 5 (left). The detailed binding mode of acarbose showed that it formed hydrogen bonding interactions with residues Asp349, His239, Asp68, Pro309, Glu304, Arg439, Arg212, Glu276 and one hydrophobic interaction with Phe157 (Fig. 5, right).

**Figure 6:**
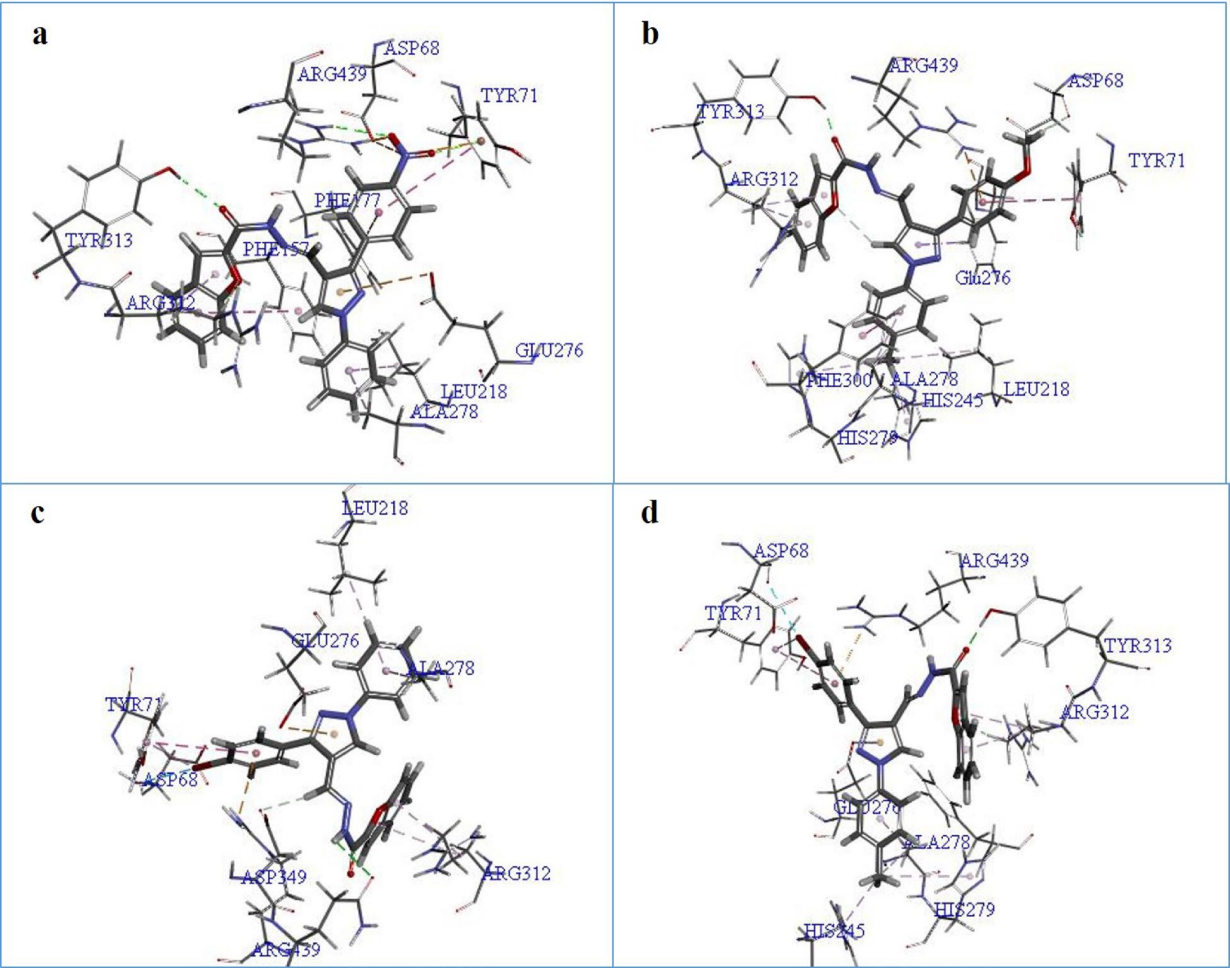
Docked conformer of compounds (**a**) **8e**, (**b**) **8l**, (**c**) **8f** and (**d**) **8n** in the active site of α-glucosidase.

The theoretical binding mode of most active compounds **8e**, **8l**, **8f** and **8n** were also shown in Fig. 6. Binding mode analysis showed that the following interactions are common among these compounds: (1) Pyrazole moiety interacts with Glu276 through *π*-anion binding. (2) Phenyl groups attached to the pyrazole moiety, on the other hand, are involved in hydrophobic interactions with Arg439, Ala278 and Tyr71. (3) The carbonyl oxygen of the amide group formed hydrogen bonds with the hydroxyl group of Tyr313, and (4) the planar benzofuran scaffold interacted with Arg312 via π-alkyl interaction. As expected, all pharmacophoric features used in the design of the hybrid, are involved in the interaction with the active site of the enzyme. The most potent compound **8e** establishes more interactions with the residues in the binding pocket. The nitro substituent of this compound created a hydrogen bond with Arg439 and also two electrostatic interactions with Asp68 and Tyr71. Besides, the N1-phenyl ring of pyrazole moiety and benzofuran also formed hydrophobic interactions with Leu218 and Phe157, respectively, which leads to a snug fit at the binding site (Fig. 6a). When the interaction mode of compound **8l** as the second most potent compound is compared to that of compound **8e**, only one hydrogen bond with Asp68 stabilizes diphenyl pyrazole moiety in **8l**, while in compound **8e** the electrostatic interactions play an important role in the binding of this moiety to the enzyme. Methyl substituent in compound **8l** is located in a hydrophobic pocket formed by residues Leu218, His245, His279, Phe300 and Ala278 (Fig. 6b). In the case of compounds **8f** and **8n**, both have bromine substitution at the R_1_ position, the presence of methyl group in R_2_ may cause their different orientations in the active site and then the difference in inhibitory activity. The predicted binding mode of compound **8f** shows that the NH proton of the amide group forms hydrogen bond interaction with the amide group of Gln350. The hydrogen bond belonging to the carbonyl oxygen of the amide group was not seen in this compound. Moreover, the CH imine group is forming a hydrogen contact with carboxyl oxygen of Asp349 and thus leads to a better fit of this compound in the enzyme's active pocket (Fig. 6c,d). The calculated GOLD Fitness Scores for compounds **8e** (70.4927), **8l** (65.7091), **8f** (64.84.4) and **8n** (61.1567) were in good agreement with those results obtained in in vitro assay.

### Molecular dynamic investigation

Molecular dynamic simulation was performed in order to understand the effect of the compound over the enzyme active site. For this purpose, the structural perturbations incurred by the most potent compound (**8e**) has been investigated through the study of the RMSD, RMSF and its effect on the active site environment in comparison to acarbose as α-glycosidase standard inhibitor and the apoenzyme.

**Figure 7:**
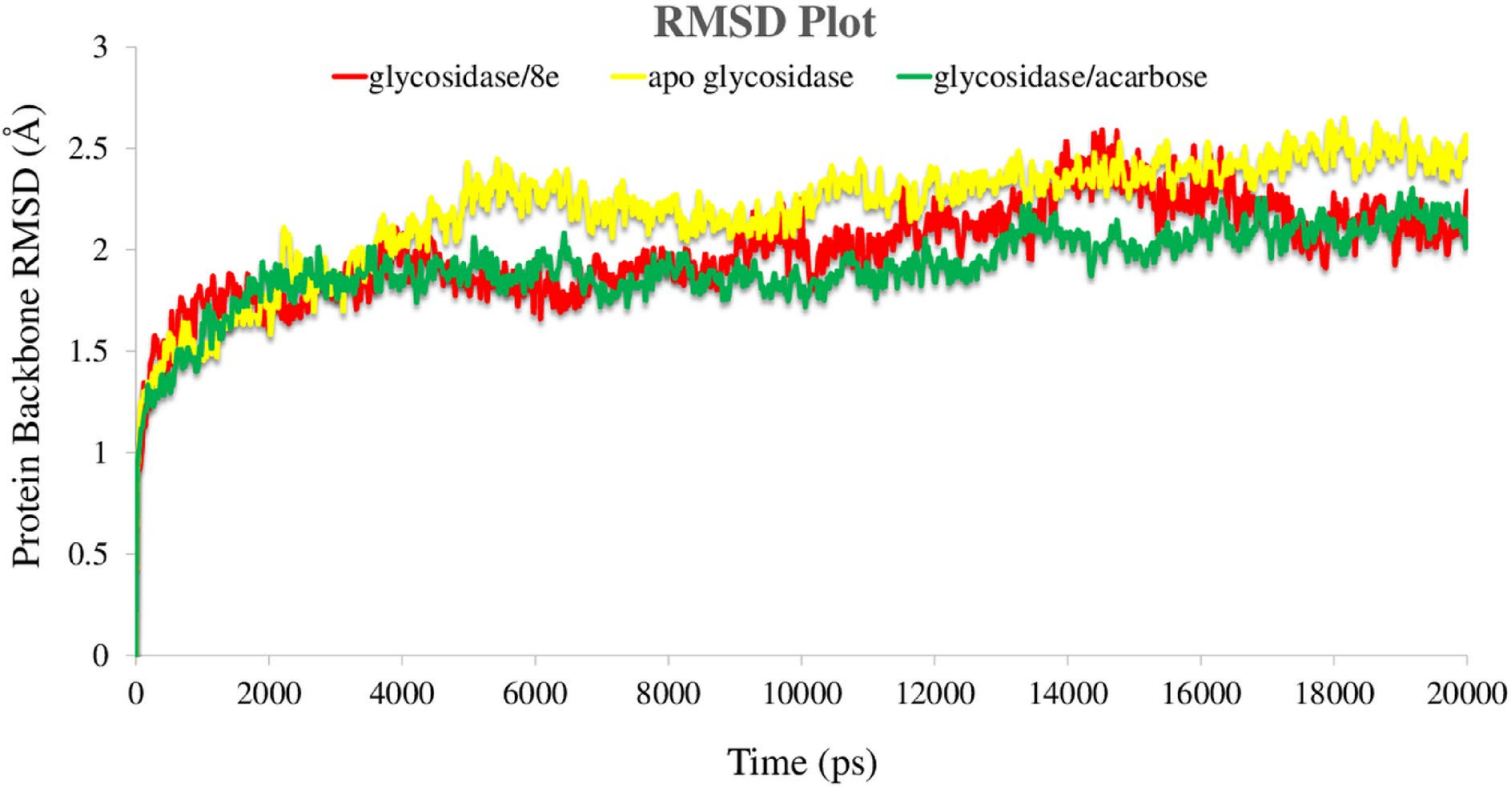
RMSD of the α-glycosidase backbone in complexed with acarbose (in green), compound **8e** (in red) and the apo enzyme (in yellow) for over 20 ns MD simulation time.

Root mean square deviation (RMSD) of the enzymes’ backbone was analyzed over 20 ns MD simulation in order to study the stability of the protein–ligand complex trajectories (Fig. 7). The RMSD value of the apo α-glycosidase depicts broad fluctuations throughout simulation time which is higher than the two enzyme complexes. The RMSD value increased after about 4 ns and steadily increased up to 16 ns and become more stable for the last 4 ns of simulation time with the value of 2.5 Å. The RMSD value of glycosidase complexed with acarbose was stable until 12 ns and slightly increased through the next 4 ns and become steady for the rest of the simulation time with the RMSD value of 2.1 Å. Although, the mentioned value of α-glycosidase complexed with compound **8e** is the same as acarbose bounded state for the first 8 ns. It is observed that compound **8e** had higher RMSD than acarbose for its higher number of rings and flexibility, which makes it more deviate from the initial structure for the next 8 ns and finally it decreased and stabilized for the last 2 ns with the same RMSD as acarbose bounded state (2.1 Å).

In summary, the RMSD value of the bounded state enzymes deviate from the initial structure of apoenzyme in the early part of the simulation and obviously decreased as a result of α-glycosidase structural rigidity. Thus, the structures at the last 2 ns of the MD equilibrium state used to investigate the structural specificity of the ligand–protein complexes.

**Figure 8:**
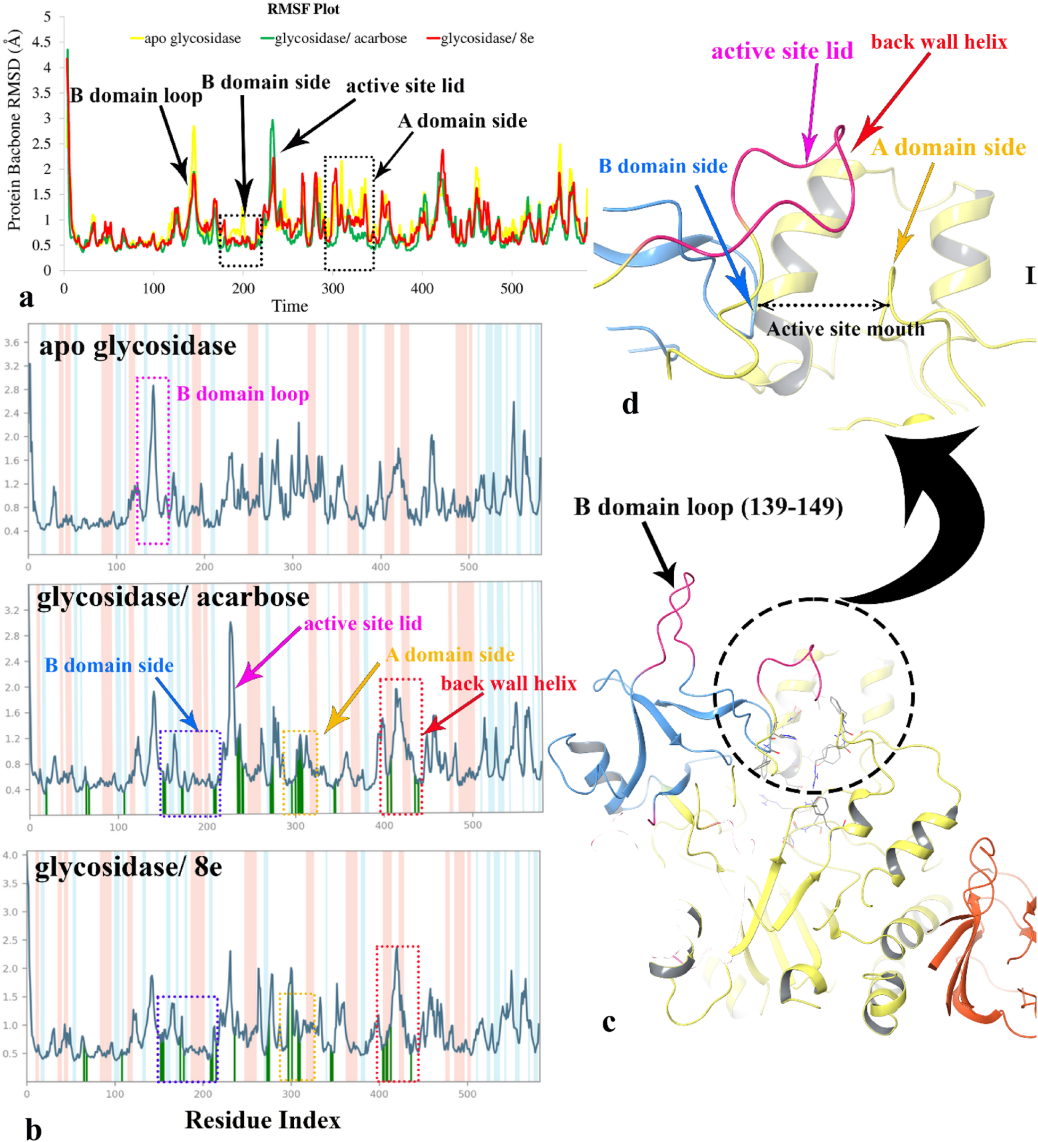
RMSF plot of the α-glycosidase backbone in complexed with compound **8e** (in red) and acarbose (in green) and the apo enzyme for over 20 ns MD simulation time (**a**). ligand binding location for over 20 ns MD simulation time. α-helical and ß-strand regions are highlighted in red and blue backgrounds, respectively (**b**). 3D representation of α-glycosidase structure. Enzyme domain of A, B and C are colored in yellow, blue, and orange, respectively. The the flexible regions are colored in pink (**c**). Close-up representation of α-glycosidase active site (**d**).

The RMSF, which indicates the flexibility of protein structure, refers to the fluctuation of the Cα atom from its average position throughout the simulation time. Figure 8a compares the residue RMSF values of α-glycosidase bound state and unbound state in which, the apoenzyme (yellow color line) had higher RMSF fluctuations compared to the glycosidase bound-states (green and red-colored lines). This observation occurs upon ligand binding to the enzyme, in which residues movement decrease as a result of non-bonding interaction between the ligand and the enzyme. In addition, the structural segments which are affected upon ligand binding have recognized and categorized into four apparent parts, including; B domain loop (residues 139–149), the active site lid, A domain and B domain sides of the active site mouth. Comparing RMSF values shows that the residues of the B domain loop, 139–149, would have significantly lower RMSF value in glycosidase/ acarbose and compound **8e** bound-state rather than apoenzyme (Fig. 8a,b). In contrast, the flexibility of the active site lid increased in enzyme bound-state and is more pronounced through acarbose binding. In order to investigate the reason for the mentioned observation, as noticed in Fig. 8b (glycosidase/ acarbose complex), acarbose interacted with several residues located into the A and B domain side of the active site mouth (the vertical green line). Although compound **8e** interacted with the same regions, it formed fewer interactions with the residues of the A domain. So, it can be proposed that the more ligand interaction with A and B domain sides of the active site mouth, the higher the RMSF of the active site lid. Moreover, Fig. 8c,d represent the organization of the α-glycosidase three main domains; A, B, and C and the close-up representation of the active site mouth with the corresponding residues of A and B domain at both sides in which the active side lid and a back-wall helix situated at the front and the back of the mentioned mouth, respectively. Backing to Fig. 8b, compound **8e** provides higher interaction with the back-wall of the active site rather than acarbose. Based on the observed result of RMSF plot, although α-glycosidase/ acarbose complex with higher interaction at the entrance region loop covering the active site (310–315) have lower RMSF value than in α-glycosidase-**8e** complex, the other lid loop covering the active site consists of residues 230–236^51^ shows significantly lower RMSF value in α-glycosidase-**8e** complex rather than α-glycosidase/acarbose complex.

Finally, based on RMSF plot, it can be proposed that compound **8e** had more interaction over the B domain side and back-wall helix of the active site, while acarbose formed more interaction with the A domain side of the α-glycosidase active site.

**Figure 9:**
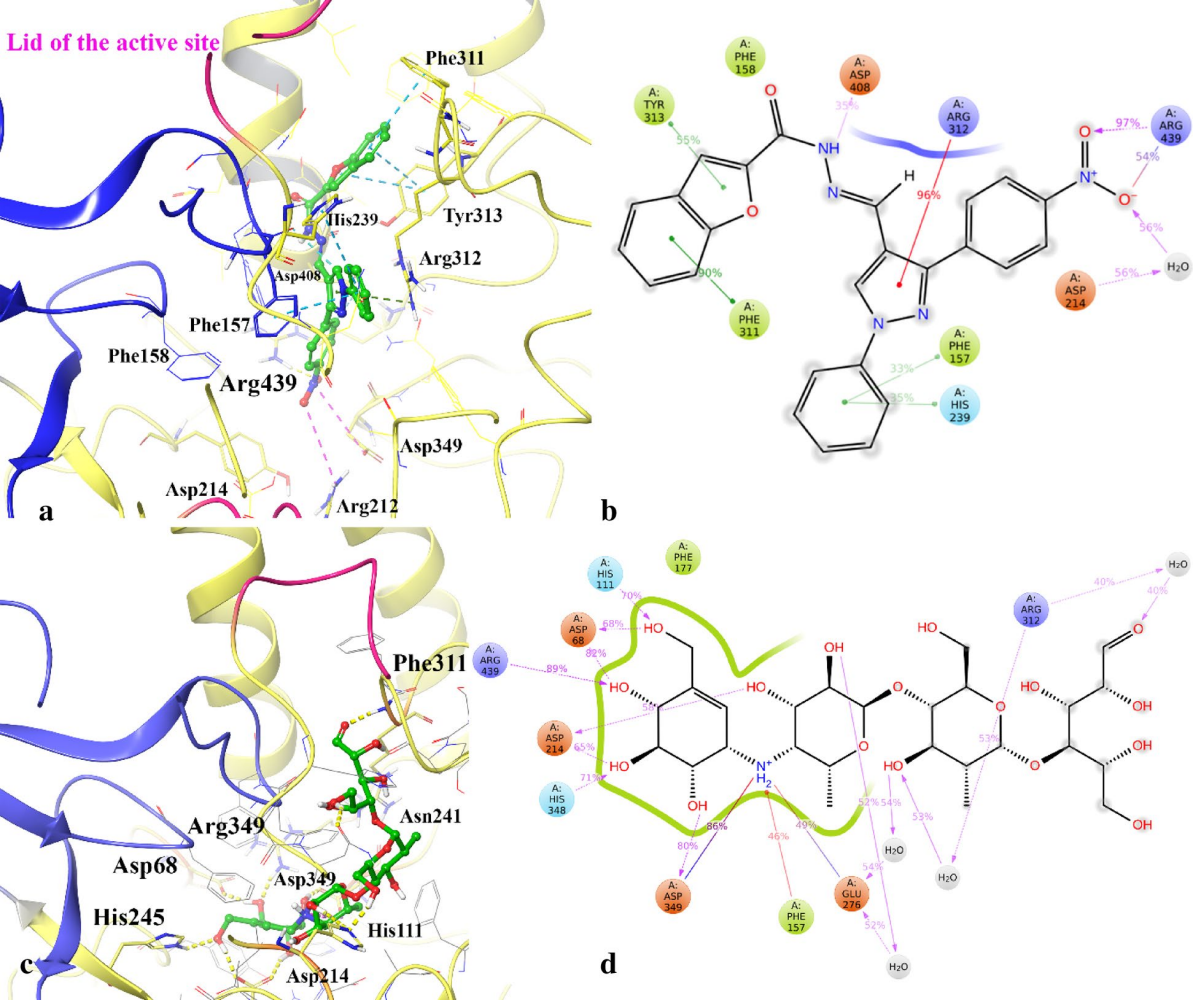
shows the detailed orientation and ligand atom interactions that occurred more than 30.0% of the simulation time during the equilibrated phase over α-glycosidase complexed with compound **8e** (**a**,**b**) and acarbose (**c**,**d**). Domain A, domain B and the flap region covered the mouth of the active site colored in yellow, blue and pink, respectively.

Figure 9a,b represent the detailed orientation and interactions that occurred more than 30% of the simulation time during the equilibrated phase over α-glycosidase complexed with compound **8e**. The interaction analysis depicts compound **8e** oriented vertically from the mouth to the bottom of the active site and stabilized the enzyme domains by interacting with Phe311, Tyr313, Arg312 from the A domain side and Phe158, Phe177 and His239 from the B domain side of the active site mouth (Fig. 9a). In addition, polar residues including; Asp214, Asp349 and Arg439 provide polar interactions with compound **8e** at the depth part of the active site. In the same way, acarbose disposed vertically and formed non-binding interactions with the Phe311, Asn241, Arg439, Asp68, His245, Asp349, Asp214, which belong to the domain A of the enzyme (Fig. 9c).

Comparing MD simulation of compound **8e** and acarbose proposed the long-lasting non-binding interactions with Asp349, Asp214 and Arg439 have a significant role in inhibition activity of the mentioned compounds (Fig. 9b,d). Figure 9a,b represent two important structural moieties in stabilizing compound **8e** at the mouth of the active site. The first one is the benzofuran ring, which interacts with Phe311 and Tyr313 through T-shape π–π hydrophobic interactions for 90% and 55% of the simulation time and the next one, pyrazole ring which interacts with Arg312 through π-cation hydrophobic interaction for 96% of the simulation time. Along with the interactions which stabilized compound **8e** in front of the A domain side of the active site entrance, the phenyl substituent interacts with Phe157 and His239, which faced at the B domain side of the entrance for about one-third of the simulation time. In addition, the hydrazide moiety can provide H-bond interaction with Asp408 at the back part of the active site (previously known as the back wall side) for about 35% of simulation time. Finally, the nitrophenyl group as a polar moiety interacts with polar catalytic residues Arg439, Asp349 and Asp215 through ion-bridge, H-bond and water-mediated H-bond interactions for a significant amount of simulation time that is similar to the behavior of the NH2 group in the acarbose (Fig. 9d). It is obvious from the MD study that H-bond, hydrophobic interactions and ion-bridge interactions have a critical role in stabilizing compound **8e** at different sides of the active site during the simulation time and cause α-glycosidase inhibition activity. This observation may propose the contribution to the higher α-glycosidase inhibition activity.

In addition to the interaction analysis, the Prime/MM-GBSA module was used to estimate the strengths of interactions between the ligand–protein complexes generated by the clustering method. ΔG_bind_ of α-glycosidase/compound **8e** complex and α-glycosidase/acarbose complex was estimated to be − 90.83 and − 62.49 kcal/mol, respectively, revealing stronger binding interaction of compound **8e** than acarbose which also supported by experimental assay.

## Conclusion

With aim of developing a novel class of α-glucosidase inhibitors, new series of biphenyl pyrazole-benzofuran hybrids derivatives were designed, synthesized and evaluated for their α-glucosidase inhibition. All screened compounds displayed multifold enhanced inhibitory strength in the range of 40.6 ± 0.2–164.3 ± 1.8 µM when compared to acarbose (IC_50_ = 750.0 ± 10.0 µM). Among them, compound **8e**, having a nitro group in R_1_ position, was found to be the most potent inhibitor. This is approximately 18-fold more potent than the standard acarbose. Also, the kinetic analysis revealed that compound **8e** compete with the substrate for binding to the binding site of the enzyme. Limited SAR studies indicated that the in vitro α-glucosidase inhibitory activity mainly depends upon the substituents on R_1_ and the nature of substituents at this position affected the efficacy of the methyl group on R_2_. Binding mode analysis showed that almost all structural features such as pyrazole ring, Phenyl groups attached to the pyrazole moiety, amide linkage and benzofuran scaffold are contributing to binding affinity through hydrogen bonding, hydrophobic and electrostatic interactions. In addition, MD simulations showed compound **8e** oriented vertically into the active site from mouth to the bottom and stabilized the enzyme domains by interacting with the interface of domain A and domain B and the back side of the active site, while acarbose formed non-binding interaction with the residue belong to the domain A of the enzyme. Moreover, carbonyl hydrazide linker, pyrazole and its related substituents provide such a strategic point with the ability to interact with various parts of the active site, which has the binding and catalytic role for α-glycosidase activity.

Taken together, the above results suggest that newly synthesized hybrids could be promising hits for the further development of α-glucosidase inhibitors for the treatment of diabetes patients.

## Experimental

All reagents and organic solvents were purchased from Sigma Chemical Co. (St. Louis, USA) and used without further purification. Thin-layer chromatography (TLC) was carried out on pre-coated silica gel aluminum plates (Merck silica gel 60, F254). Melting points of target compound **8a–o** were measured on a Kofler hot stage apparatus and were uncorrected. ^1^H NMR and ^13^C NMR spectra were recorded on Bruker FT-500 spectrometer (Bruker, Rheinstetten, Germany) in DMSO-d_6_ with tetramethylsilane (TMS) as the internal standard. IR spectra were recorded on Nicolet Magna FTIR 550 spectrophotometer (resolution 2 cm^−1^) in KBr pellets. Elemental analysis was carried out with an Elemental Analyzer system GmbH VarioEL CHNS mode (Germany). Mass spectra were recorded on Agilent 5975C Mass Spectrometer.

### General procedure for the preparation of 1,3-disubstituted-4-pyrazole carbaldehydes *4a-n*

To a solution of 4-substituted phenylhydrazine hydrochloride **2** (20 mmol) in ethanol (15 mL), substituted acetophenone **1** (20 mmol) and catalytic amounts of sulfuric acid was added and then the mixture was refluxed for 8–12 h. After reaction completion, the mixture was cooled to room temperature and poured on crushed ice to afford hydrazone intermediate **3a–n**. The resulting solid was filtered and recrystallized from ethanol. A solution of hydrazone **3a–n** (20 mmol) in DMF (5 mL) was added drop-wise to an ice-cold solution of DMF (15 mL) and phosphorus oxychloride (60 mmol) and the resulting mixture refluxed at 60–70 °C for 5–8 h. After the completion of the reaction, the reaction mixture allowed to cool, poured into ice-cold water and then neutralized with saturated aqueous sodium hydroxide solution. Further, the solid precipitated was filtered, washed with excess cold water and recrystallized from ethanol to afford aldehydes **4a–n**.

### General procedure for the preparation of ethyl benzofuran-2-carboxylate *6*

A mixture of salicylaldehyde (20 mmol), ethyl bromoacetate (20 mmol) and K_2_CO_3_ (40 mmol) in acetonitrile (10 mL) heated under reflux for 4 h. After completion of the reaction (monitored by TLC), the reaction mixture was allowed to cool to room temperature and poured into crushed ice. After extracting the product with ethyl acetate (2 × 25 mL), the organic layer was washed using brine solution (2 × 20 mL) and dried over anhydrous MgSO_4_. The solvent was evaporated under vacuum to afford the product as an oil.

### General procedure for the preparation of benzofuran-2-carbohydrazide *7*

benzofuran-2-carboxylate 6 (20 mmol) and hydrazine hydrate (30 mmol) in EtOH (10 mL) heat under reflux overnight. Upon cooling, the product precipitated was filtered, washed with cold water and recrystallized in methanol to afford the pure product.

### General procedure for the preparation of pyrazole-benzofuran hybrids *8a-n*

To a mixture of the appropriate pyrazole aldehydes 4 (1 mmol) and a catalytic amount of glacial acetic acid (3–4 drops) in absolute ethanol (10 mL), were added hydrazide 7 (1.1 mmol) and refluxed for 12–18 h. As the reaction was completed, the mixture was allowed to cool to room temperature, the precipitate was filtered off and crystallized from ethanol to give the pure final derivatives **8a–n** (see related spectra in Supplementary data).

#### N'-((1,3-Diphenyl-1H-pyrazol-4-yl)methylene)benzofuran-2-carbohydrazide (8a)

Creamish powder; yield: 86%, mp 210–211 °C. IR (KBr, cm^−1^): 3345(N–H), 3080–2980 (C–H), 1656(C=O), 15,615(C=N), 1435(C=C).^1^H NMR (400 MHz, DMSO-*d*_6_) δ 12.22 (s, 1H, NH), 9.15 (s, 1H, H_5_-pyr), 8.79 (s, 1H, CH=N), 8.16 (d, *J* = 7.9 Hz, 2H, Ar–H), 7.88–7.79 (m, 4H, Ar–H), 7.65 (m, 7H, Ar–H), 7.49 (m, 2H, Ar–H). ^13^C NMR (101 MHz, DMSO) δ 154.35, 154.20, 151.99, 148.06, 142.00, 139.00, 131.89, 129.57, 128.76, 128.61, 128.44, 127.15, 127.12, 127.00, 123.85, 122.85, 118.85, 116.74, 111.82, 110.69. EI-MS m/z (% rel. abund.): 406 (M^+^, 14), 245 (100), 161 (64), 145 (70), 104 (9), 89 (29), 77 (34). Anal. Calcd for C_25_H_18_N_4_O_2_: C, 73.88; H, 4.46; N, 13.78. Found: C, 73.21; H, 4.24; N, 13.92.

#### N'-((1-Phenyl-3-p-tolyl-1H-pyrazol-4-yl)methylene)benzofuran-2-carbohydrazide (8b)

Creamish powder; yield: 81%, mp 234–235 °C. IR (KBr, cm^−1^): 3385(N–H), 3010–2923 (C–H), 1648(C=O), 1555(C=N), 1450(C=C). ^1^H NMR (400 MHz, DMSO-*d*_6_) δ 12.20 (s, 1H, NH), 9.13 (s, 1H, H_5_-pyr), 8.77 (s, 1H, CH=N), 8.15 (d, *J* = 7.9 Hz, 2H, Ar–H), 7.93 (m, *J* = 7.7 Hz,1H, Ar–H), 7.80–7.82 (m, 2H, Ar–H), 7.76 (d, *J* = 8.0 Hz, 2H, Ar–H), 7.63 (m, 3H, Ar–H), 7.48 (m, 4H, Ar–H), 2.52 (s, 3H, CH_3_). ^13^C NMR (101 MHz, DMSO) δ 154.34, 154.19, 152.04, 148.07, 142.14, 139.02, 138.07, 129.55, 129.31, 129.05, 128.33, 127.15, 127.02, 126.99, 126.93, 123.85, 122.84, 118.81, 116.64, 111.82, 110.67, 20.86. EI-MS m/z (% rel. abund.): 420 (M^+^, 14), 259 (100), 161 (30), 145 (43), 104 (6), 89 (21), 77 (21). Anal. Calcd for C_26_H_20_N_4_O_2_: C, 74.27; H, 4.79; N, 13.33. Found: C, 74.83; H, 3.99; N, 13.73.

#### N'-((3-(4-Methoxyphenyl)-1-phenyl-1H-pyrazol-4-yl)methylene)benzofuran-2-carbohydrazide (8c)

White powder; yield: 78%, mp 207–209 °C. IR (KBr, cm^−1^): 3408(N–H), 3214–2980 (C–H), 1670(C=O), 1537(C=N), 1446(C=C). ^1^H NMR (400 MHz, DMSO-*d*_6_) δ 12.22 (s, 1H, NH), 9.12 (s, 1H, H_5_-pyr), 8.77 (s, 1H, CH=N), 8.15 (d, *J* = 8.0 Hz, 2H, Ar–H), 7.94 (d, *J* = 7.7 Hz, 1H, Ar–H), 7.81–7.83 (m, 4H, Ar–H), 7.68–7.60 (m, 3H, Ar–H), 7.49 (t, *J* = 7.4 Hz, 2H, Ar–H), 7.23 (d, *J* = 8.6 Hz, 2H, Ar–H), 3.96 (s, 3H, OCH_3_). ^13^C NMR (101 MHz, DMSO) δ 159.59, 154.34, 154.18, 151.85, 148.08, 142.20, 139.03, 129.76, 129.54, 127.15, 127.00, 126.86, 124.27, 123.85, 122.84, 118.75, 116.46, 114.17, 111.82, 110.67, 55.25. EI-MS m/z (% rel. abund.): 436 (M^+^, 24), 275 (100), 276 (24), 260 (14), 161 (19), 145 (39), 104 (5), 89 (19), 77 (20). Anal. Calcd for C_26_H_20_N_4_O_3_: C, 71.55; H, 4.62; N, 12.84. Found: C, 71.83; H, 4.21; N, 12.20.

#### (E)-N'-((3-(4-hydroxyphenyl)-1-phenyl-1H-pyrazol-4-yl)methylene)benzofuran-2-carbohydrazide (8d)

Creamish powder; yield: 81%, mp 280–281 °C. IR (KBr, cm^−1^): 3506(N–H), 2996–3195 (C–H), 1646(C=O), 1521(C=N), 1469(C=C). ^1^H NMR (400 MHz, DMSO-*d*_6_) δ 12.20 (s, 1H, NH), 9.09 (s, 1H, H_5_-pyr), 8.76 (s, 1H, CH=N), [8.14 (d, *J* = 7.8 Hz, 2H), 7.93 (d, *J* = 7.8 Hz, 1H), 7.81–7.83 (m, 2H), 7.46–7.70 (m, 6H), 7.46–7.50 (m, 2H), 7.05 (d, *J* = 8.5 Hz, 2H)] (15H, Ar–H and OH). ^13^C NMR (101 MHz, DMSO) δ 157.97, 154.34, 154.18, 152.25, 148.09, 142.37, 139.06, 129.75, 129.53, 127.13, 126.99, 126.82, 126.77, 123.84, 122.84, 122.61, 118.69, 116.30, 115.51, 111.82, 110.63. EI-MS m/z (% rel. abund.): 422 (M^+^, 18), 261 (100), 262 (21), 161 (25), 145 (59), 89 (26), 77 (21). Anal. Calcd for C_25_H_18_N_4_O_3_: C, 71.08; H, 4.29; N, 13.26. Found: C, 71.68; H, 4.49; N, 13.76.

#### N'-((3-(4-Nitrophenyl)-1-phenyl-1H-pyrazol-4-yl)methylene)benzofuran-2-carbohydrazide (8e)

Yellow powder; yield: 73%, mp 241–243 °C. IR (KBr, cm^−1^): 3105(N–H), 2907 (C–H), 1669(C=O), 1552(C=N), 1444(C=C). ^1^H NMR (400 MHz, DMSO-*d*_6_) δ 12.31 (s, 1H, NH), 9.21 (s, 1H, H_5_-pyr), 8.82 (s, 1H, CH=N), 8.39–8.50 (m, 2H, Ar–H), 8.22–8.05 (m, 3H, Ar–H), 7.93 (d, *J* = 7.7 Hz, 1H, Ar–H), 7.89–7.75 (m, 2H, Ar–H), 7.75–7.56 (m, 4H, Ar–H), 7.35–7.46 (m, 2H, Ar–H). ^13^C NMR (101 MHz, DMSO) δ 154.36, 154.27, 149.31, 147.98, 147.15, 141.28, 138.77, 138.41, 129.61, 129.50, 128.55, 127.37, 127.22, 126.98, 123.87, 123.78, 123.53, 122.88, 119.00, 118.76, 117.52, 111.82, 110.90. EI-MS m/z (% rel. abund.): 451 (M^+^, 6), 290 (11), 275 (12), 259 (18), 161 (100), 145 (81), 89 (30), 77 (25). Anal. Calcd for C_25_H_17_N_5_O_4_: C, 66.51; H, 3.80; N, 15.51. Found: C, 65.83; H, 3.12; N, 14.93.

#### N'-((3-(4-Bromophenyl)-1-phenyl-1H-pyrazol-4-yl)methylene)benzofuran-2-carbohydrazide (8f)

White powder; yield: 88%, mp 243–245 °C. IR (KBr, cm^−1^): 3430(N–H), 2845–3165 (C–H), 1655(C=O), 1538(C=N), 1442(C=C). ^1^H NMR (400 MHz, DMSO-*d*_6_) δ 12.24 (s, 1H, NH), 9.19 (s, 1H, H_5_-pyr), 8.78 (s, 1H, CH=N), 8.17 (d, *J* = 7.9 Hz, 2H, Ar–H), 7.95 (d, *J* = 7.7 Hz, 1H, Ar–H), 7.91–7.81 (m, 6H, Ar–H), 7.82–7.96 (m, 3H, Ar–H), 7.48–7.54 (m, 2H, Ar–H). ^13^C NMR (101 MHz, DMSO) δ 154.35, 154.20, 150.69, 148.03, 141.72, 138.91, 131.66, 131.12, 130.49, 129.59, 127.69, 127.19, 127.12, 126.98, 123.87, 122.87, 122.07, 118.89, 116.83, 111.83, 110.77. EI-MS m/z (% rel. abund.): 487 (M^+^ + 2, 3), 485 (M^+^, 3), 325 (79), 323 (79), 161 (86), 145 (100), 89 (40), 77 (40). Anal. Calcd for C_25_H_17_BrN_4_O_2_: C, 61.87; H, 3.53; N, 11.54. Found: C, 61.16; H, 3.21; N, 12.04.

#### N'-((3-(4-Chlorophenyl)-1-phenyl-1H-pyrazol-4-yl)methylene)benzofuran-2-carbohydrazide (8g)

Creamish powder; yield: 81%, mp 241–243 °C. IR (KBr, cm^−1^): 3491(N–H), 2945–3223 (C–H), 1652(C=O), 1553(C=N), 1503(C=C). ^1^H NMR (400 MHz, DMSO-*d*_6_) δ 12.26 (s, 1H, NH), 9.17 (s, 1H, H_5_-pyr), 8.78 (s, 1H, CH=N), 8.16 (d, *J* = 7.9 Hz, 2H, Ar–H), 7.92–7.95 (m, 3H, Ar–H), 7.85–7.79 (m, 2H, Ar–H), 7.73 (d, *J* = 8.4 Hz, 2H), 7.66 (t, *J* = 8.0 Hz, 3H, Ar–H), 7.47–7.52 (m, 2H, Ar–H). ^13^C NMR (101 MHz, DMSO) δ 154.34, 150.59, 148.24, 141.66, 138.91, 133.39, 130.79, 130.19, 129.58, 128.74, 127.63, 127.11, 127.01, 123.83, 122.82, 118.86, 116.90, 111.80, 110.64. EI-MS m/z (% rel. abund.): 442 (M^+^ + 2, 4), 440 (M^+^, 13), 281 (35), 279 (100), 161 (80), 145 (91), 89 (37), 77 (34). Anal. Calcd for C_25_H_17_ClN_4_O_2_: C, 68.11; H, 3.89; N, 12.71. Found: C, 68.53; H, 3.16; N, 12.52.

#### N'-((3-(4-Fluorophenyl)-1-phenyl-1H-pyrazol-4-yl)methylene)benzofuran-2-carbohydrazide (8h)

White powder; yield: 84%, mp 253–254 °C. IR (KBr, cm^−1^): 3500 (N–H), 2926–3093 (C–H), 1661(C=O), 1553(C=N), 1441(C=C). ^1^H NMR (400 MHz, DMSO-*d*_6_) δ 12.22 (s, 1H, NH), 9.15 (s, 1H, H_5_-pyr), 8.76 (s, 1H, CH=N), 8.15 (d, *J* = 7.9 Hz, 2H, Ar–H), 7.92–7.96 (m, 3H, Ar–H), 7.80–7.82 (m, 2H), 7.60–7.67 (m, 3H), 7.53–7.46 (m, 4H). ^13^C NMR (101 MHz, DMSO) δ 163.58, 161.14, 154.34, 154.20, 150.95, 148.04, 141.82, 138.94, 130.65, 130.57, 129.57, 128.42, 127.41, 127.18, 127.03, 126.98, 123.86, 122.86, 118.84, 116.69, 115.76, 115.55, 111.82, 110.74. EI-MS m/z (% rel. abund.): 424 (M^+^, 15), 263 (100), 161 (70), 145 (83), 89 (32), 77 (32). Anal. Calcd for C_25_H_17_FN_4_O_2_: C, 70.75; H, 4.04; N, 13.20. Found: C, 70.26; H, 4.34; N, 13.61.

#### N'-((1-Phenyl-3-(4-(trifluoromethyl)phenyl)-1H-pyrazol-4-yl)methylene)benzofuran-2-carbohydrazide (8i)

White powder; yield: 78%, mp 217–218 °C. IR (KBr, cm^−1^): 337 (N–H), 2909–3050 (C–H), 1665(C=O), 1555(C=N), 1442(C=C). ^1^H NMR (400 MHz, DMSO-*d*_6_) δ 12.25 (s, 1H, NH), 9.23 (s, 1H, H_5_-pyr), 8.80 (s, 1H, CH=N), 8.21–8.13 (m, 4H, Ar–H), 8.04 (d, *J* = 8.2 Hz, 2H, Ar–H), 7.94 (d, *J* = 7.8 Hz, 1H, Ar–H), 7.81–7.84 (m, 2H, Ar–H), 7.61–7.70 (m, 3H, Ar–H), 7.47–7.54 (m, 2H, Ar–H). ^13^C NMR (101 MHz, DMSO) δ 154.35, 154.21, 150.33, 148.00, 141.53, 138.86, 135.96, 129.62, 129.24, 128.94, 128.62, 127.94, 127.26, 127.21, 126.98, 125.59, 125.55, 123.88, 122.87, 118.96, 117.15, 111.82, 110.83. EI-MS m/z (% rel. abund.): 474 (M^+^, 7), 313 (20), 161 (100), 145 (78), 89 (24), 77 (20). Anal. Calcd for C_26_H_17_F_3_N_4_O_2_: C, 65.82; H, 3.61; N, 11.81. Found: C, 65.21; H, 3.32; N, 11.10.

#### N'-((3-Phenyl-1-p-tolyl-1H-pyrazol-4-yl)methylene)benzofuran-2-carbohydrazide (8j)

White powder; yield: 81%, mp 255–256 °C. IR (KBr, cm^−1^): 3420(N–H), 2917–3050 (C–H), 1646(C=O), 1537(C=N), 1442(C=C). ^1^H NMR (400 MHz, DMSO-*d*_6_) δ 12.20 (s, 1H, NH), 9.08 (s, 1H, H_5_-pyr), 8.77 (s, 1H, CH=N), 8.02 (d, *J* = 8.4 Hz, 2H, Ar–H), 7.87–7.78 (m, 4H, Ar–H), 7.69–7.56 (m, 5H, Ar–H), 7.43–7.49 (m, 3H, Ar–H), 2.46 (s, 3H, CH_3_). ^13^C NMR (101 MHz, DMSO) δ 154.34, 154.19, 151.75, 148.08, 142.06, 136.80, 136.42, 131.96, 129.93, 128.74, 128.54, 128.42, 127.14, 126.99, 126.90, 123.84, 122.84, 118.77, 116.51, 111.81, 110.66, 20.47. EI-MS m/z (% rel. abund.): 420 (M^+^, 9), 259 (100), 161 (23), 145 (35), 91 (20), 77(9). Anal. Calcd for C_26_H_20_N_4_O_2_: C, 74.27; H, 4.79; N, 13.33. Found: C, 74.66; H, 4.32; N, 13.91.

#### N'-((1,3-Dip-tolyl-1H-pyrazol-4-yl)methylene)benzofuran-2-carbohydrazide (8k)

White powder; yield: 83%, mp 259–261 °C. IR (KBr, cm^−1^): 3450(N–H), 2945–3065 (C–H), 1665(C=O), 1580(C=N), 1455(C=C). ^1^H NMR (400 MHz, DMSO-*d*_6_) δ 12.18 (s, 1H, NH), 9.05 (s, 1H, H_5_-pyr), 8.76 (s, 1H, CH=N), 8.01 (d, *J* = 8.2 Hz, 2H, Ar–H), 7.91 (d, *J* = 7.7 Hz, 1H, Ar–H), 7.79–7.81 (m, 2H, Ar–H), 7.74 (d, *J* = 7.8 Hz, 2H, Ar–H), 7.60 (t, *J* = 7.7 Hz, 1H, Ar–H), 7.48–7.38 (m, 5H, Ar–H), 2.50 (s, 3H, CH_3_), 2.46 (s, 3H, CH_3_). ^13^C NMR (101 MHz, DMSO) δ 154.34, 154.18, 151.80, 148.09, 142.20, 137.98, 136.82, 136.33, 129.92, 129.56, 129.28, 129.12, 128.75, 128.30, 127.13, 126.99, 126.80, 123.84, 122.83, 118.72, 116.41, 111.81, 110.64, 20.85, 20.46. EI-MS m/z (% rel. abund.): 434 (M^+^, 16), 273 (100), 161 (24), 145 (37), 91 (19). Anal. Calcd for C_27_H_22_N_4_O_2_: C, 74.64; H, 5.10; N, 12.89. Found: C, 74.25; H, 5.42; N, 12.24.

#### N'-((3-(4-Methoxyphenyl)-1-p-tolyl-1H-pyrazol-4-yl)methylene)benzofuran-2-carbohydrazide (8l)

White powder; yield: 82%, mp 230–232 °C. IR (KBr, cm^−1^): 3442(N–H), 2890–3120 (C–H), 1670(C=O), 1540(C=N), 1450(C=C). ^1^H NMR (400 MHz, DMSO-*d*_6_) δ 12.20 (s, 1H, NH), 9.06 (s, 1H, H_5_-pyr), 8.76 (s, 1H, CH=N), 8.03 (d, *J* = 8.4 Hz, 2H, Ar–H), 7.94 (d, *J* = 7.7 Hz, 1H, Ar–H), 7.80–7.83 (m, 4H, Ar–H), 7.62 (t, *J* = 7.5 Hz, 1H, Ar–H), 7.50–7.43 (m, 3H, Ar–H), 7.23 (d, *J* = 8.7 Hz, 2H, Ar–H), 3.96 (s, 3H, OCH_3_), 2.48 (s, 3H, CH_3_). ^13^C NMR (101 MHz, DMSO) δ 159.55, 154.34, 154.16, 151.62, 148.10, 142.25, 136.83, 136.26, 129.92, 129.73, 127.14, 126.99, 126.80, 124.35, 123.85, 122.84, 118.68, 116.23, 114.15, 111.82, 110.63, 55.25, 20.47. EI-MS m/z (% rel. abund.): 450 (M^+^, 4), 313 (20), 259 (33), 161 (100), 145 (96), 89 (38), 77 (38), 43 (34). Anal. Calcd for C_27_H_22_N_4_O_3_: C, 71.99; H, 4.92; N, 12.44. Found: C, 71.43; H, 4.36; N, 12.72.

#### N'-((3-(4-Hydroxyphenyl)-1-p-tolyl-1H-pyrazol-4-yl)methylene)benzofuran-2-carbohydrazide (8m)

Creamish powder; yield: 82%, mp 293–295 °C. IR (KBr, cm^−1^): 3460(N–H), 2990–3165 (C–H), 1666(C=O), 1540(C=N), 1430(C=C). ^1^H NMR (400 MHz, DMSO-*d*_6_) δ 12.19 (s, 1H, NH), 9.02 (s, 1H, H_5_-pyr), 8.75 (s, 1H, CH=N), [8.00 (d, *J* = 8.4 Hz, 2H), 7.92 (d, *J* = 7.6 Hz, 1H), 7.80–7.82 (m, 2H), 7.59–7.68 (m, 4H), 7.49–7.40 (m, 3H), 7.04 (d, *J* = 8.5 Hz, 2H)] (14H, Ar–H and OH), 2.46 (s, 3H, CH_3_). ^13^C NMR (101 MHz, DMSO) δ 157.92, 154.33, 154.16, 152.03, 148.10, 142.42, 136.86, 136.16, 129.90, 129.72, 127.12, 126.99, 126.61, 123.84, 122.83, 122.68, 118.62, 116.07, 115.50, 111.82, 110.61, 20.46. EI-MS m/z (% rel. abund.): 436 (M^+^, 17), 275 (100), 161 (21), 145 (41), 89 (20). Anal. Calcd for C_26_H_20_N_4_O_3_: C, 71.55; H, 4.62; N, 12.84. Found: C, 71.10; H, 4.15; N, 12.28.

#### N'-((3-(4-Bromophenyl)-1-p-tolyl-1H-pyrazol-4-yl)methylene)benzofuran-2-carbohydrazide (8n)

White powder; yield: 75%, mp 258–259 °C. IR (KBr, cm^−1^): 3425(N–H), 2898–3098 (C–H), 1666(C=O), 1535(C=N), 1450(C=C). ^1^H NMR (400 MHz, DMSO-*d*_6_) δ 12.19 (s, 1H, NH), 9.10 (s, 1H, H_5_-pyr), 8.75 (s, 1H, CH=N), 8.02 (d, *J* = 8.3 Hz, 2H, Ar–H), 7.92 (d, *J* = 7.7 Hz, 1H, Ar–H), 7.82–7.85 (m, 5H, Ar–H), 7.61 (t, *J* = 7.7 Hz, 1H, Ar–H), 7.44–7.50 (m, 4H, Ar–H), 2.47 (s, 3H, CH_3_). ^13^C NMR (101 MHz, DMSO) δ 154.34, 154.18, 150.45, 148.04, 141.77, 136.71, 136.55, 131.64, 131.19, 130.45, 129.95, 127.47, 127.18, 126.98, 123.87, 122.86, 121.99, 118.80, 116.61, 111.82, 110.75, 20.47. EI-MS m/z (% rel. abund.): 501 (M^+^ + 2, 3), 499 (M^+^, 3), 339 (100), 337 (100), 161 (56), 145 (75), 91 (34), 69 (32), 57 (40), 43 (47). Anal. Calcd for C_26_H_19_BrN_4_O_2_: C, 62.54; H, 3.84; N, 11.22. Found: C, 62.91; H, 3.52; N, 11.76.

#### N'-((3-(4-Chlorophenyl)-1-p-tolyl-1H-pyrazol-4-yl)methylene)benzofuran-2-carbohydrazide (8o)

White powder; yield: 83%, mp 248–249 °C. IR (KBr, cm^−1^): 3435(N–H), 2925–3050 (C–H), 1675(C=O), 1542(C=N), 1438(C=C). ^1^H NMR (400 MHz, DMSO-*d*_6_) δ 12.22 (s, 1H, NH), 9.09 (s, 1H, H_5_-pyr), 8.74 (s, 1H, CH=N), 8.02 (d, *J* = 8.4 Hz, 2H, Ar–H), 7.90–7.94 (m, 3H, Ar–H), 7.82–7.76 (m, 2H, Ar–H), 7.72 (d, *J* = 8.4 Hz, 2H, Ar–H), 7.63–7.57 (m, 1H, Ar–H), 7.45–7.49 (m, 3H, Ar–H), 2.48 (s, 3H, CH_3_). ^13^C NMR (101 MHz, DMSO) δ 154.30, 150.24, 141.39, 136.74, 136.47, 133.26, 130.93, 130.11, 129.95, 128.71, 127.30, 127.15, 126.82, 123.70, 122.65, 118.74, 116.96, 111.74, 110.10, 20.47. EI-MS m/z (% rel. abund.): 456 (M^+^ + 2, 5), 454 (M^+^, 14), 295 (35), 293 (100), 161 (57), 145 (77), 89 (34). Anal. Calcd for C_26_H_19_ClN_4_O_2_: C, 68.65; H, 4.21; N, 12.32. Found: C, 68.99; H, 4.53; N, 12.81.

### In vitro α-glucosidase inhibition assay

Stock solutions of the test compounds and acarbose were prepared in DMSO and eventually diluted with phosphate buffer saline to attain the desired concentration. A mixture containing various concentrations of test compound and enzyme (Saccharomyces cerevisiae, EC3.2.1.20, 0.2 U/mL) in phosphate buffer saline was added to the 96-well plate and incubated at 37 °C for 10 min. Then, the enzyme-catalyzed reaction was commenced by the addition of p-nitrophenyl-α-glucopyranoside as substrate (25 μL, 4 mM) and the absorbance was measured spectrophotometrically at 400 nm after 20 min incubation at 37 °C. DMSO (10% final concentration) and acarbose were used as the control and standard inhibitor, respectively. The percentage of enzyme inhibition for each entry was calculated using the following formula:

% Inhibition = [(Abs control—Abs sample)/Abs control] × 100.

The concentrations of the compounds that inhibited 50% of *α*-glucosidase activity (IC_50_ values) were calculated from non-linear regression curve using the Logit method.

### Kinetic study

The kinetics of enzyme inhibition was performed according to the reaction conditions in 2.5 by preparing a series of test solutions in which the concentration of the substrate (PNPG) was varied (2–10 mM) in the absence and presence of compound **8e** at different concentrations (0, 10, 25, and 40 µM). A Lineweaver–Burk plot was generated to identify the type of inhibition and the Michaelis–Menten constant (*K*_*m*_) value was determined from the plot between reciprocal of the substrate concentration (1/[S]) and reciprocal of enzyme rate (1/V) over various inhibitor concentrations. The experimental inhibitor constant (*K*_*i*_) value was constructed by secondary plots of the inhibitor concentration [I] versus *K*_m_.

### Cytotoxicity evaluation of the most potent compounds on 3T3 cell line

The cytotoxicity of the compounds 8e, 8l, 8f and 8n was determined using the 3-(4,5 Dimethylthiazol-2-yl)-2,5-diphenyltetrazolium bromide (MTT) assay according to previously described methods^44^.

### Homology modeling and docking study

The primary sequence of Saccharomyces cerevisiae α-glucosidase downloaded from UniProtKB database (Uni- ProtKB, http://www.uniprot.org/) with accession number P53341. Hence, a search was executed to identify a protein with a high sequence similarity using NCBI BLAST server (https://blast.ncbi.nlm.nih.gov/Blast.cgi). The crystallographic structure of Saccharomyces cerevisiae (PDB ID: 3A4A) was chosen as a template and subjected to sequence alignment using Align sequence to template protocol in Discovery Studio v4.1 (DS) (Accelrys, San Diego, CA) (http://accelrys.com). The 3D structure of α-glucosidase for S cerevisiae was predicted using the modeler module of the DS4.1. The number of models was set to 10, and the optimization level was changed to high. DOPE score and profile-3D for preliminary evaluation of developed model were carried out using verify protein protocol in DS. Ramachandran plot with PROCHECK program (http://servicesn.mbi.ucla.edu/PROCHECK) was also applied to verify the quality of the obtained homology model^52,53^. With the modeled structure, the docking of selected compounds was carried out.

The predicted model was subjected to protein preparation using the prepare protein protocol in DS software. In this step, the complex was typed with CHARMm force field, hydrogen atoms were added, all water molecules were removed and pH of protein was adjusted to almost neutral, 7.4. The 3D structure of the most active compounds **8e**, **8l**, **8f** and **8n** were built using the Marvin v15.4.6 program (https://chemaxon.com/products/marvin) and then were transferred into DS. Ligand structures were typed with CHARMm force field and partial charges were calculated by Momany-Rone option. Subsequently, resulting structures were minimized with Smart Minimizer, which performs 1000 steps of steepest descent with a RMS gradient tolerance of 3, followed by conjugate gradient minimization. Molecular docking approach has been carried out using genetic algorithm based docking program (GOLD v5.3.0) (https://www.ccdc.cam.ac.uk/solutions/csd-discovery/components/gold/), which considers complete flexibility of side chains of amino acids at the active site. A 9 A˚ radius sphere was defined as a docking pocket. Acarbose and most potent compounds were docked into the active site of the protein and generated binding modes of ligands were ranked based on GOLD score fitness function. A top-score binding pose was selected for analyzing the interactions between the enzyme and the inhibitors using Discovery Studio Visualizer v4.1 (http://accelrys.com/products/discovery-studio)^54^.

### Molecular dynamic simulation

Molecular dynamic (MD) simulation of this study was performed by using the Desmond v5.3 module (https://www.schrodinger.com/products/desmond) implemented in Maestro interface (from Schrödinger 2018‐4 suite)^55^. The appropriate pose for MD simulation procedure of the compounds was obtained by the docking method.

In order to build the system for MD simulation, the protein–ligand complexes were solvated with SPC explicit water molecules and placed in the center of an orthorhombic box of appropriate size in the Periodic Boundary Condition. Sufficient counter‐ions and a 0.15 M solution of NaCl were also utilized to neutralize the system and to simulate the real cellular ionic concentrations, respectively. The MD protocol involved minimization, pre-production, and finally, production MD simulation steps. In the minimization procedure, the entire system was allowed to relax for 2500 steps by the steepest descent approach. Then the temperature of the system was raised from 0 to 300 K with a small force constant on the enzyme in order to restrict any drastic changes. MD simulations were performed via NPT (constant number of atoms, constant pressure i.e. 1.01325 bar and constant temperature i.e. 300 K) ensemble. The Nose‐Hoover chain method was used as the default thermostat with 1.0 ps interval and Martyna‐Tobias‐Klein as the default barostat with 2.0 ps interval by applying isotropic coupling style. Long‐range electrostatic forces were calculated based on Particle‐mesh‐based Ewald approach with the he cut‐off radius for columbic forces set to 9.0 Å. Finally, the system was ubjected to produce MD simulations for 20 ns for the protein–ligand complex. During the simulation, every 1000 ps of the actual frame was stored. The dynamic behavior and structural changes of the systems were analyzed by the calculation of the root mean square deviation (RMSD) and RMSF. Subsequently, the energy-minimized structure calculated from the equilibrated trajectory system was evaluated to investigate of each ligand–protein complex interaction.

### Prime MM-GBSA

The ligand binding energies (ΔG_Bind_) were calculated for compound **8o** and acarbose using Molecular mechanics/generalized born surface area (MM-GBSA) modules (Schrödinger LLC 2018) (75) based on the following equation;

$$\Delta {\text{G}}\;{\text{Bind = E}}_{{{\text{Complex}}}} - \left[ {E_{{{\text{receptor}}}} + E_{{{\text{Ligand}}}} } \right]$$

where ΔG Bind is the calculated relative free energy which includes both ligand and receptor strain energy. E_Complex_ is the MM-GBSA energy of the minimized complex, and E_Ligand_ is the MM-GBSA energy of the ligand after removing it from the complex and allowing it to relax. E_Receptor_ is the MM-GBSA energy of relaxed protein after separating it from the ligand. The MM-GBSA calculation was performed based on the clustering method for energy calculation.

## Supplementary Information

Supplementary Information.
