# Supplementary material for: Design, synthesis, biological evaluation, and molecular modeling studies of pyrazole-benzofuran hybrids as new α-glucosidase inhibitor
Source: Sci Rep. 2021 Oct 21;11:20776. doi: 10.1038/s41598-021-99899-1 (PMC8531348; doi:10.1038/s41598-021-99899-1)
Supplement: Supplementary file 1 — Supplementary Information. [file 41598_2021_99899_MOESM1_ESM.docx]

**Supplementary Information**

Design and synthesis of novel pyrazole-benzofuran hybrids: *in vitro* α-glucosidase inhibitory activity, kinetic and molecular modeling study

Fateme Azimi ^a^, Homa Azizian ^b^, Mohammad Najafi ^c^, Ghadamali khodarahmi ^a^, Lotfollah Saghaei ^a^, Motahareh hassanzadeh ^a^, Jahan B. Ghasemi ^d^, Mohammad Ali Faramarzi ^e^, Bagher Larijani ^f^, [Farshid Hassanzadeh](https://www.sciencedirect.com/science/article/pii/S0968089603005248#!) ^a,*^, Mohammad Mahdavi ^f,*^

*^a^ Bioinformatics Research Center, School of Pharmacy and Pharmaceutical Sciences, Isfahan University of Medical Sciences, 81746-73461 Isfahan, Iran*

*^b^ Department of Medicinal Chemistry, School of Pharmacy-International Campus, Iran University of Medical Science, Tehran, Iran*

*^c^ Department of Chemistry, Isfahan University of Technology, Isfahan, 84156-83111, Iran*

*^d^ School of Chemistry, University College of Science, University of Tehran, P.O. Box 14155-6455, Tehran, Iran*

*^e^ Department of Pharmaceutical Biotechnology, Faculty of Pharmacy, Tehran University of Medical Sciences, P.O. Box 14155-6451, Tehran 1417614411, Iran*

*^f^ Endocrinology and Metabolism Research Center, Endocrinology and Metabolism Research Institute, Tehran University of Medical Sciences, Tehran, Iran*

* Corresponding authors.

E-mail addresses: [hassanzadeh@pharm.mui.ac.ir](mailto:hassanzadeh@pharm.mui.ac.ir) (F. Hassanzadeh), momahdavi@tums.ac.ir (M. Mahdavi)

***List of Figures***

**Fig. S1.** ^1^H NMR spectra of N'-((1,3-Diphenyl-1H-pyrazol-4-yl)methylene)benzofuran-2-carbohydrazide (8a)……………………………………………………………………………………………………...…..6

**Fig. S2.** ^13^C NMR spectra of N'-((1,3-Diphenyl-1H-pyrazol-4-yl)methylene)benzofuran-2-carbohydrazide (8a)………………………………………………………..…………….…………………………………..7

**Fig. S3.** Mass spectra of N'-((1,3-Diphenyl-1H-pyrazol-4-yl)methylene)benzofuran-2-carbohydrazide (8a) ……………………………………………………………………....………………………….…………..8

**Fig. S4.** ^1^H NMR spectra of N'-((1-Phenyl-3-p-tolyl-1H-pyrazol-4-yl)methylene)benzofuran-2-carbohydrazide(8b)……………….………………………………………………….…….………………9

**Fig. S5.** ^13^C NMR spectra of N'-((1-Phenyl-3-p-tolyl-1H-pyrazol-4-yl)methylene)benzofuran-2-carbohydrazide(8b)………………………………………………………………………………………..10

**Fig. S6.** Mass spectra of N'-((1-Phenyl-3-p-tolyl-1H-pyrazol-4-yl)methylene)benzofuran-2-carbohydrazide (8b)………………………………………………………….……………………………………………..11

**Fig. S7.** ^1^H NMR spectra of N'-((3-(4-Methoxyphenyl)-1-phenyl-1H-pyrazol-4-yl)methylene)benzofuran-2-carbohydrazide (8c)………………………………………………………………………………….....12

**Fig. S8.** ^13^C NMR spectra of N'-((3-(4-Methoxyphenyl)-1-phenyl-1H-pyrazol-4-yl)methylene)benzofuran-2-carbohydrazide (8c)………………………………………………………….…………………………13

**Fig. S9.** Mass spectra of N'-((3-(4-Methoxyphenyl)-1-phenyl-1H-pyrazol-4-yl)methylene)benzofuran-2-carbohydrazide (8c)……………………………………………………………………………………….14

**Fig. S10.** 1H NMR spectra of N'-((3-(4-hydroxyphenyl)-1-phenyl-1H-pyrazol-4-yl)methylene)benzofuran-2-carbohydrazide (8d)…………………………………………………………………………………….15

**Fig. S11.** ^13^C NMR spectra of N'-((3-(4-hydroxyphenyl)-1-phenyl-1H-pyrazol-4-yl)methylene)benzofuran-2-carbohydrazide (8d)……………………………………………………………….……………………16

**Fig. S12.** Mass spectra of N'-((3-(4-hydroxyphenyl)-1-phenyl-1H-pyrazol-4-yl)methylene)benzofuran-2-carbohydrazide (8d)……………………………………………………………………………………….17

**Fig. S13.** ^1^H NMR spectra of N'-((3-(4-Nitrophenyl)-1-phenyl-1H-pyrazol-4-yl)methylene)benzofuran-2-carbohydrazide (8e)……………………………………………………………………………………….18

**Fig. S14.** ^13^C NMR spectra of N'-((3-(4-Nitrophenyl)-1-phenyl-1H-pyrazol-4-yl)methylene)benzofuran-2-carbohydrazide (8e)……………………………………………………………………………………….19

**Fig. S15.** Mass spectra of N'-((3-(4-Nitrophenyl)-1-phenyl-1H-pyrazol-4-yl)methylene)benzofuran-2-carbohydrazide (8e)……………………………………………………………………………………….20

**Fig. S16.** ^1^H NMR spectra of N'-((3-(4-Bromophenyl)-1-phenyl-1H-pyrazol-4-yl)methylene)benzofuran-2-carbohydrazide (8f)…………………………………………………………………………………......21

**Fig. S17.** ^13^C NMR spectra of N'-((3-(4-Bromophenyl)-1-phenyl-1H-pyrazol-4-yl)methylene)benzofuran-2-carbohydrazide (8f)…………………………………………………………………………………….22

**Fig. S18.** Mass spectra of N'-((3-(4-Bromophenyl)-1-phenyl-1H-pyrazol-4-yl)methylene)benzofuran-2-carbohydrazide (8f)………………………………………………………………………………………23

**Fig. S19.** ^1^H NMR spectra of N'-((3-(4-Chlorophenyl)-1-phenyl-1H-pyrazol-4-yl)methylene)benzofuran-2-carbohydrazide (8g)……………………………………………………………………………………..24

**Fig. S20.** ^13^C NMR spectra of N'-((3-(4-Chlorophenyl)-1-phenyl-1H-pyrazol-4-yl)methylene)benzofuran-2-carbohydrazide (8g)…………………………………………………………………………………….25

**Fig. S21.** Mass spectra of N'-((3-(4-Chlorophenyl)-1-phenyl-1H-pyrazol-4-yl)methylene)benzofuran-2-carbohydrazide (8g)………………………………………………………………………………………26

**Fig. S22.** ^1^H NMR spectra of N'-((3-(4-Fluorophenyl)-1-phenyl-1H-pyrazol-4-yl)methylene)benzofuran-2-carbohydrazide (8h)……………………………………………………………………………………….27

**Fig. S23.** ^13^C NMR spectra of N'-((3-(4-Fluorophenyl)-1-phenyl-1H-pyrazol-4-yl)methylene)benzofuran-2-carbohydrazide (8h)…………………………………………………………………………………….28

**Fig. S24.** Mass spectra of N'-((3-(4-Fluorophenyl)-1-phenyl-1H-pyrazol-4-yl)methylene)benzofuran-2-carbohydrazide (8h)………………………………………………………………………………………29

**Fig. S25.** ^1^H NMR spectra of N'-((1-Phenyl-3-(4-(trifluoromethyl)phenyl)-1H-pyrazol-4-yl)methylene)benzofuran-2-carbohydrazide (8i)…………………………………………………………30

**Fig. S26.** ^13^C NMR spectra of N'-((1-Phenyl-3-(4-(trifluoromethyl)phenyl)-1H-pyrazol-4-yl)methylene)benzofuran-2-carbohydrazide (8i)…………………………………………………………31

**Fig. S27.** Mass spectra of N'-((1-Phenyl-3-(4-(trifluoromethyl)phenyl)-1H-pyrazol-4-yl)methylene)benzofuran-2-carbohydrazide (8i)…………………………………………………………32

**Fig. S28.** ^1^H NMR spectra of N'-((3-Phenyl-1-p-tolyl-1H-pyrazol-4-yl)methylene)benzofuran-2-carbohydrazide (8j)……………………………………………………………………………………….33

**Fig. S29.** ^13^C NMR spectra of N'-((3-Phenyl-1-p-tolyl-1H-pyrazol-4-yl)methylene)benzofuran-2-carbohydrazide (8j)……………………………………………………………………………………….34

**Fig. S30.** Mass spectra of N'-((3-Phenyl-1-p-tolyl-1H-pyrazol-4-yl)methylene)benzofuran-2-carbohydrazide (8j)……………………………………………………………………………………….35

**Fig. S31.** ^1^H NMR spectra of N'-((1,3-Dip-tolyl-1H-pyrazol-4-yl)methylene)benzofuran-2-carbohydrazide (8k)………………………………………………….…………………………………………………….36

**Fig. S32.** ^13^C NMR spectra of N'-((1,3-Dip-tolyl-1H-pyrazol-4-yl)methylene)benzofuran-2-carbohydrazide (8k)………………………………………………………………..………………………………………37

**Fig. S33.** Mass spectra of N'-((1,3-Dip-tolyl-1H-pyrazol-4-yl)methylene)benzofuran-2-carbohydrazide (8k)…………………………………………………….………………………………………………….38

**Fig. S34.** ^1^H NMR spectra of N'-((3-(4-Methoxyphenyl)-1-p-tolyl-1H-pyrazol-4-yl)methylene)benzofuran-2-carbohydrazide (8l)………………………………………………………………..……………………39

**Fig. S35.** ^13^C NMR spectra of N'-((3-(4-Methoxyphenyl)-1-p-tolyl-1H-pyrazol-4-yl)methylene)benzofuran-2-carbohydrazide (8l)…………………………………………………………40

**Fig. S36.** Mass spectra of N'-((3-(4-Methoxyphenyl)-1-p-tolyl-1H-pyrazol-4-yl)methylene)benzofuran-2-carbohydrazide (8l)…………………………………………………….………………………………….41

**Fig. S37.** 1H NMR spectra of N'-((3-(4-Hydroxyphenyl)-1-p-tolyl-1H-pyrazol-4-yl)methylene)benzofuran-2-carbohydrazide (8m)……………………………………..... ………………42

**Fig. S38.** ^13^C NMR spectra of N'-((3-(4-Hydroxyphenyl)-1-p-tolyl-1H-pyrazol-4-yl)methylene)benzofuran-2-carbohydrazide (8m) ………………………………………………………...43

**Fig. S39.** Mass spectra of N'-((3-(4-Hydroxyphenyl)-1-p-tolyl-1H-pyrazol-4-yl)methylene)benzofuran-2-carbohydrazide (8m)………………………………………………………………………………………44

**Fig. S40.** ^1^H NMR spectra of N'-((3-(4-Bromophenyl)-1-p-tolyl-1H-pyrazol-4-yl)methylene)benzofuran-2-carbohydrazide (8n)………………………………………………………………………………………..45

**Fig. S41.** ^13^C NMR spectra of N'-((3-(4-Bromophenyl)-1-p-tolyl-1H-pyrazol-4-yl)methylene)benzofuran-2-carbohydrazide (8n)……………………………………………………………………………………..46

**Fig. S42.** Mass spectra of N'-((3-(4-Bromophenyl)-1-p-tolyl-1H-pyrazol-4-yl)methylene)benzofuran-2-carbohydrazide (8n)……………………………………………………………………………………….47

**Fig. S43.** ^1^H NMR spectra of N'-((3-(4-Chlorophenyl)-1-p-tolyl-1H-pyrazol-4-yl)methylene)benzofuran-2-carbohydrazide (8o)……………………………………………………………………………………….48

**Fig. S44.** ^13^C NMR spectra of N'-((3-(4-Chlorophenyl)-1-p-tolyl-1H-pyrazol-4-yl)methylene)benzofuran-2-carbohydrazide (8o)…………………….……………………………………………………………….49

**Fig. S45.** Mass spectra of N'-((3-(4-Chlorophenyl)-1-p-tolyl-1H-pyrazol-4-yl)methylene)benzofuran-2-carbohydrazide (8o)…………………………….…………………………………………………………50


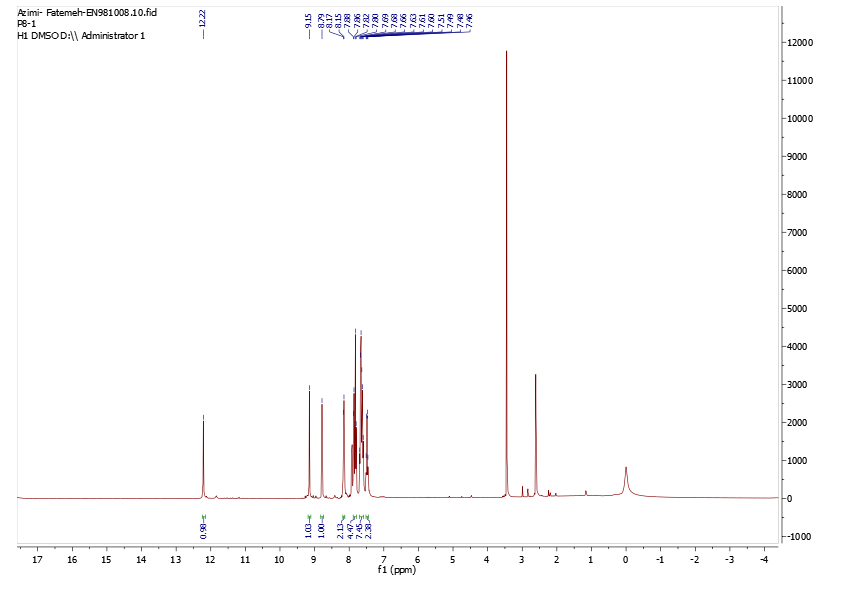


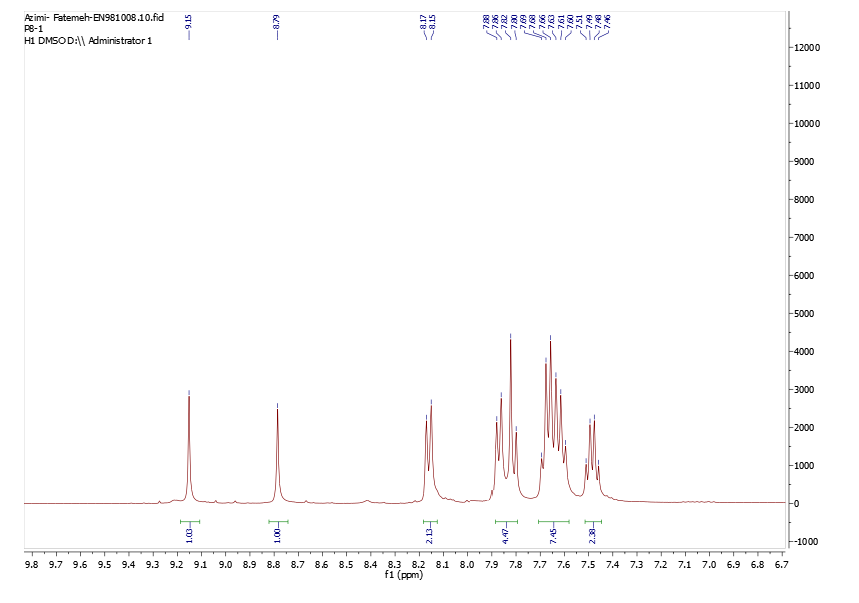


**Fig. S1.** ^1^H NMR spectra of N'-((1,3-Diphenyl-1H-pyrazol-4-yl)methylene)benzofuran-2-carbohydrazide (8a)


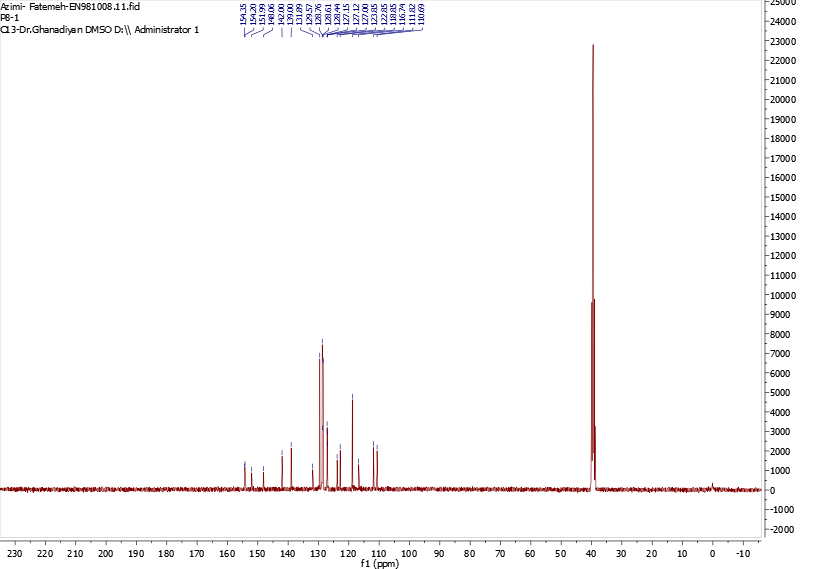


**Fig. S2.** ^13^C NMR spectra of N'-((1,3-Diphenyl-1H-pyrazol-4-yl)methylene)benzofuran-2-carbohydrazide (8a)


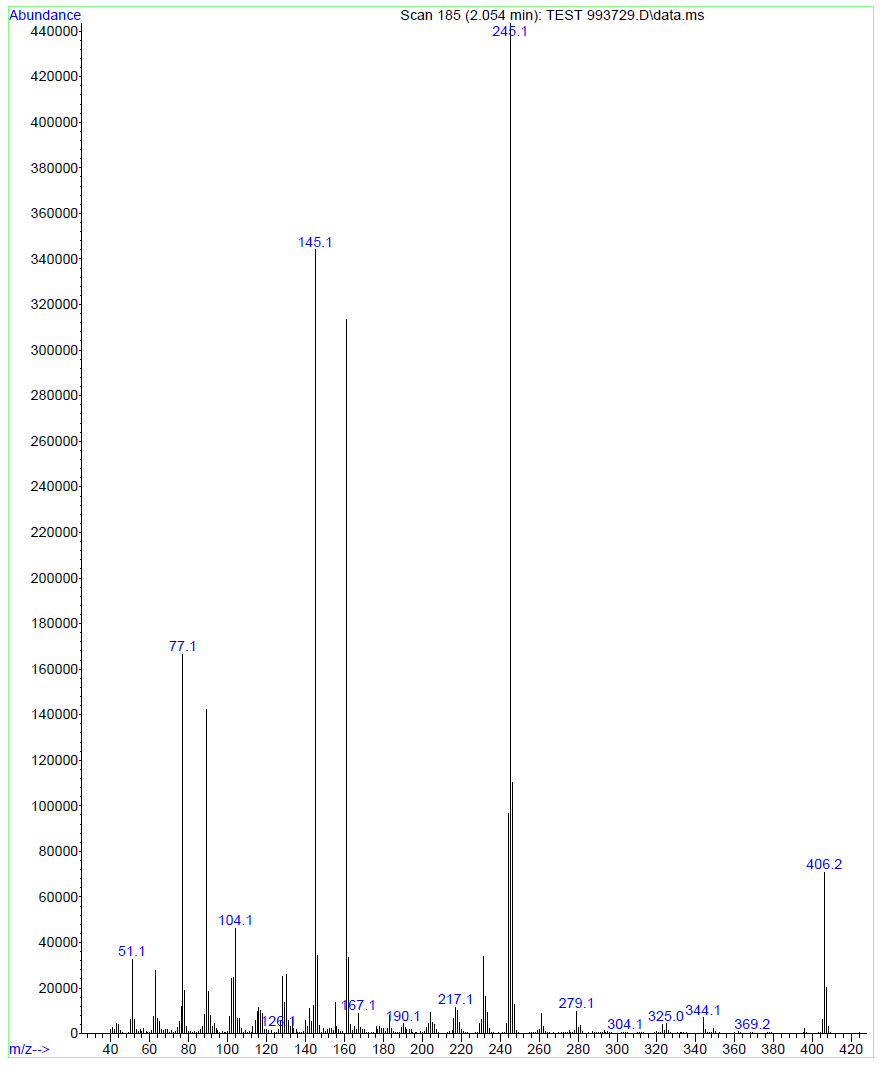


**Fig. S3.** Mass spectra of N'-((1,3-Diphenyl-1H-pyrazol-4-yl)methylene)benzofuran-2-carbohydrazide (8a)


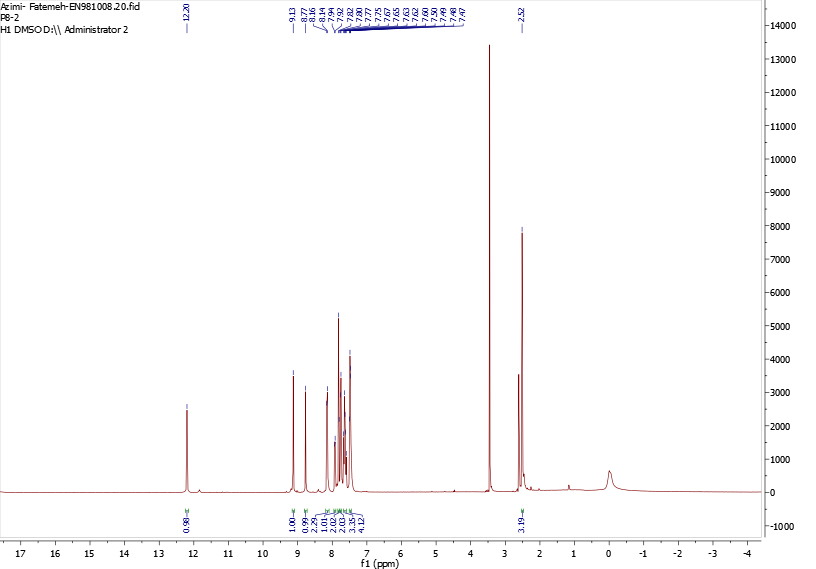


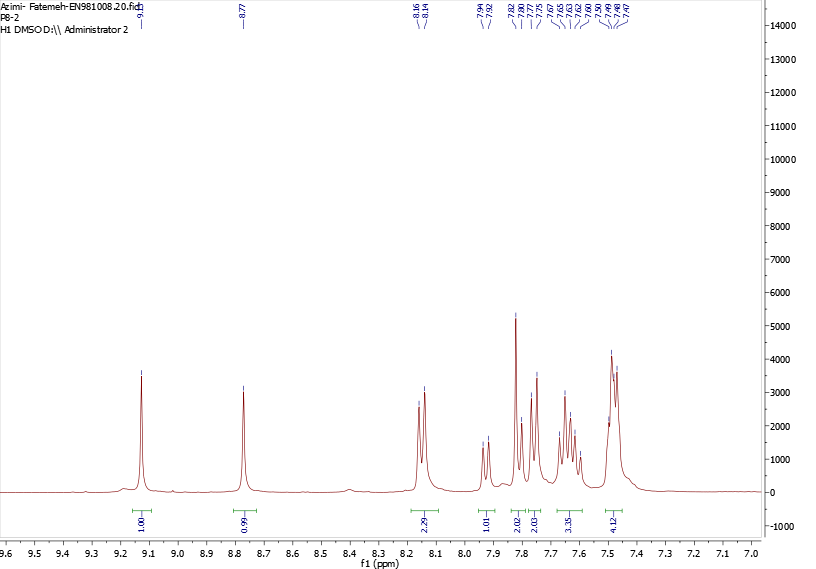


**Fig. S4.** ^1^H NMR spectra of N'-((1-Phenyl-3-p-tolyl-1H-pyrazol-4-yl)methylene)benzofuran-2-carbohydrazide (8b)


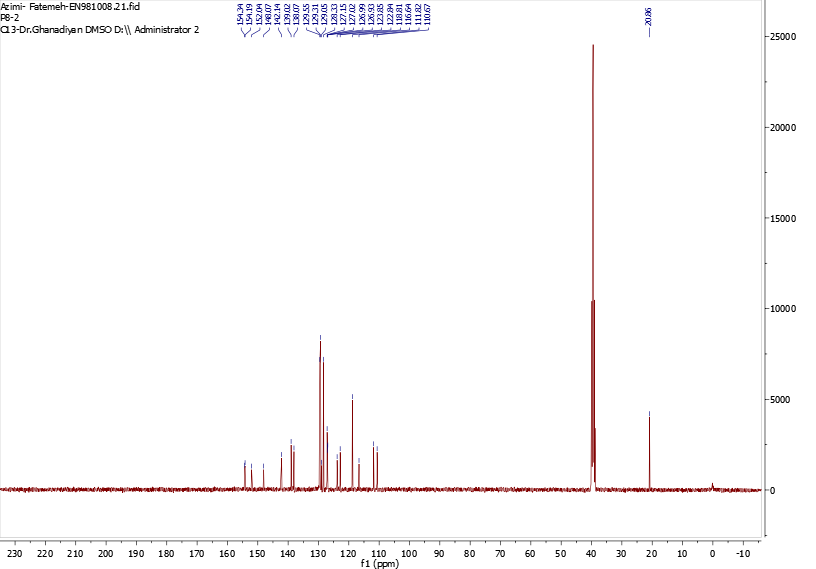


**Fig. S5.** ^13^C NMR spectra of N'-((1-Phenyl-3-p-tolyl-1H-pyrazol-4-yl)methylene)benzofuran-2-carbohydrazide (8b)


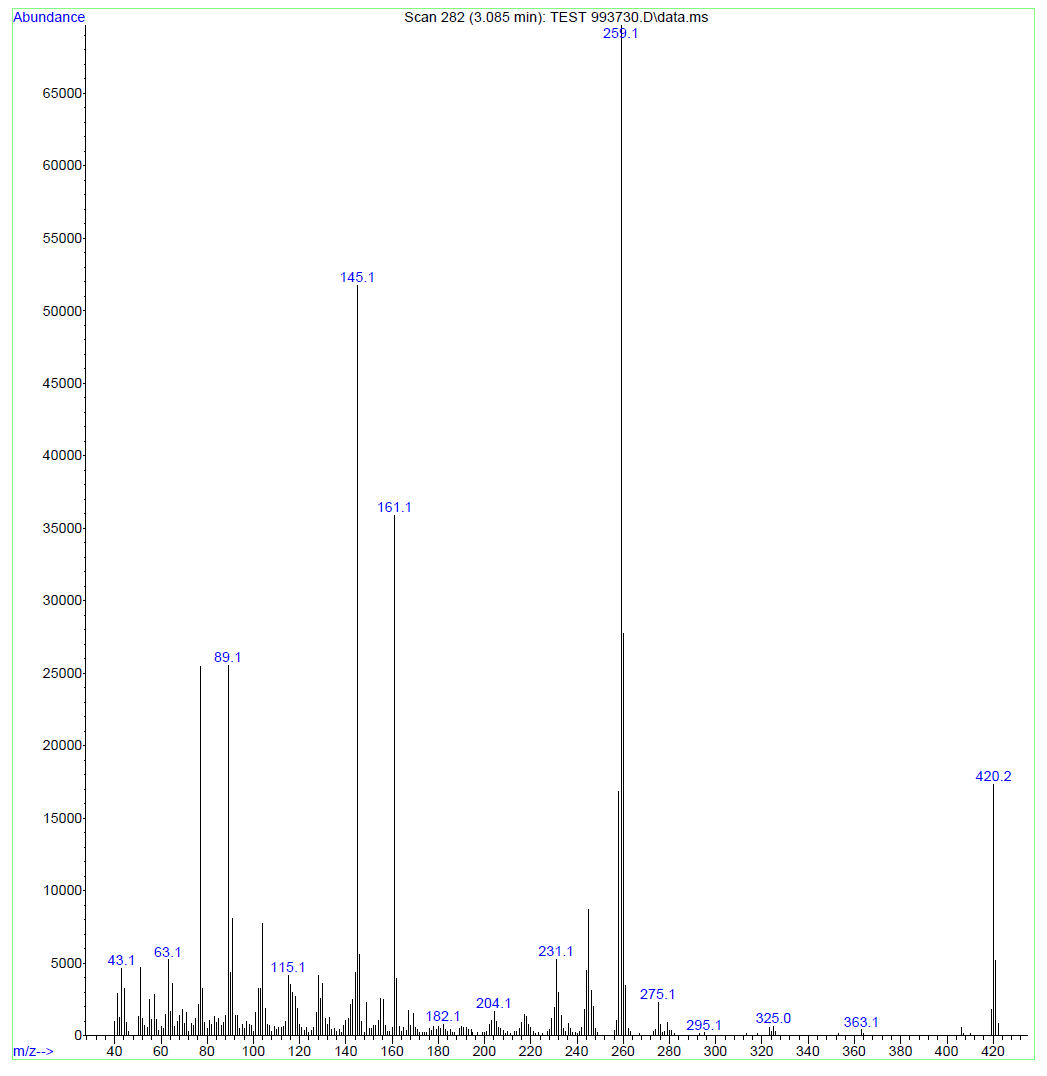


**Fig. S6.** Mass spectra of N'-((1-Phenyl-3-p-tolyl-1H-pyrazol-4-yl)methylene)benzofuran-2-carbohydrazide (8b)


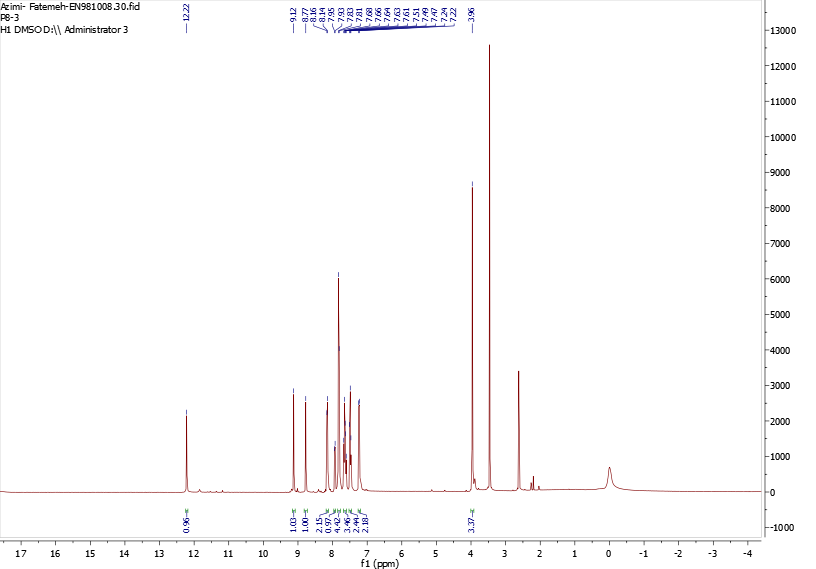


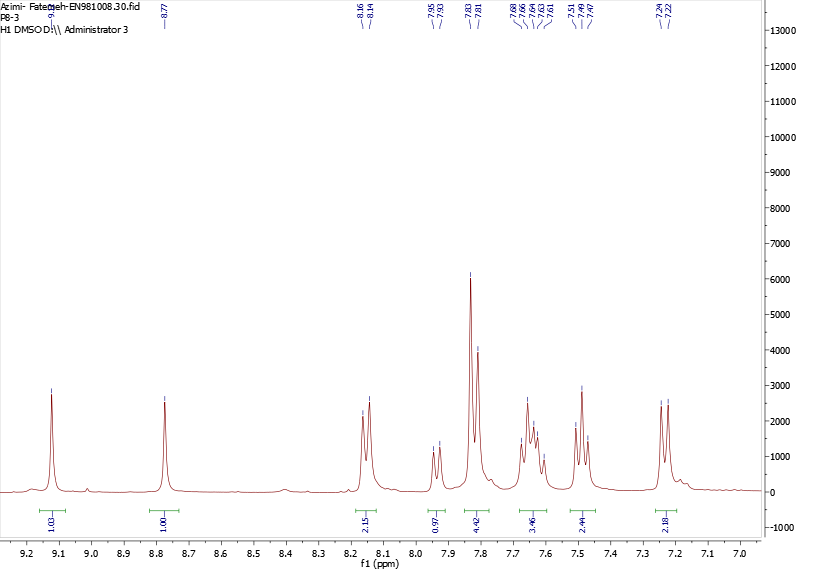


**Fig. S7.** ^1^H NMR spectra of N'-((3-(4-Methoxyphenyl)-1-phenyl-1H-pyrazol-4-yl)methylene)benzofuran-2-carbohydrazide (8c)


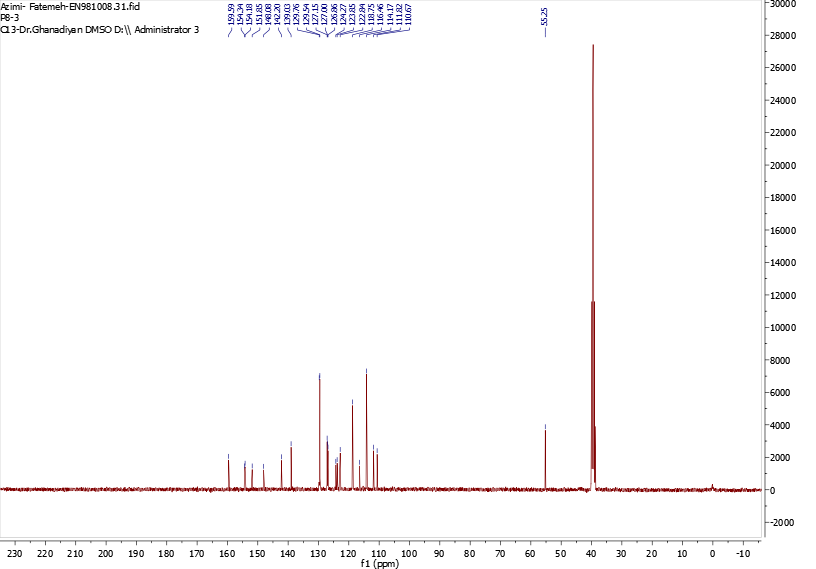


**Fig. S8.** ^13^C NMR spectra of N'-((3-(4-Methoxyphenyl)-1-phenyl-1H-pyrazol-4-yl)methylene)benzofuran-2-carbohydrazide (8c)


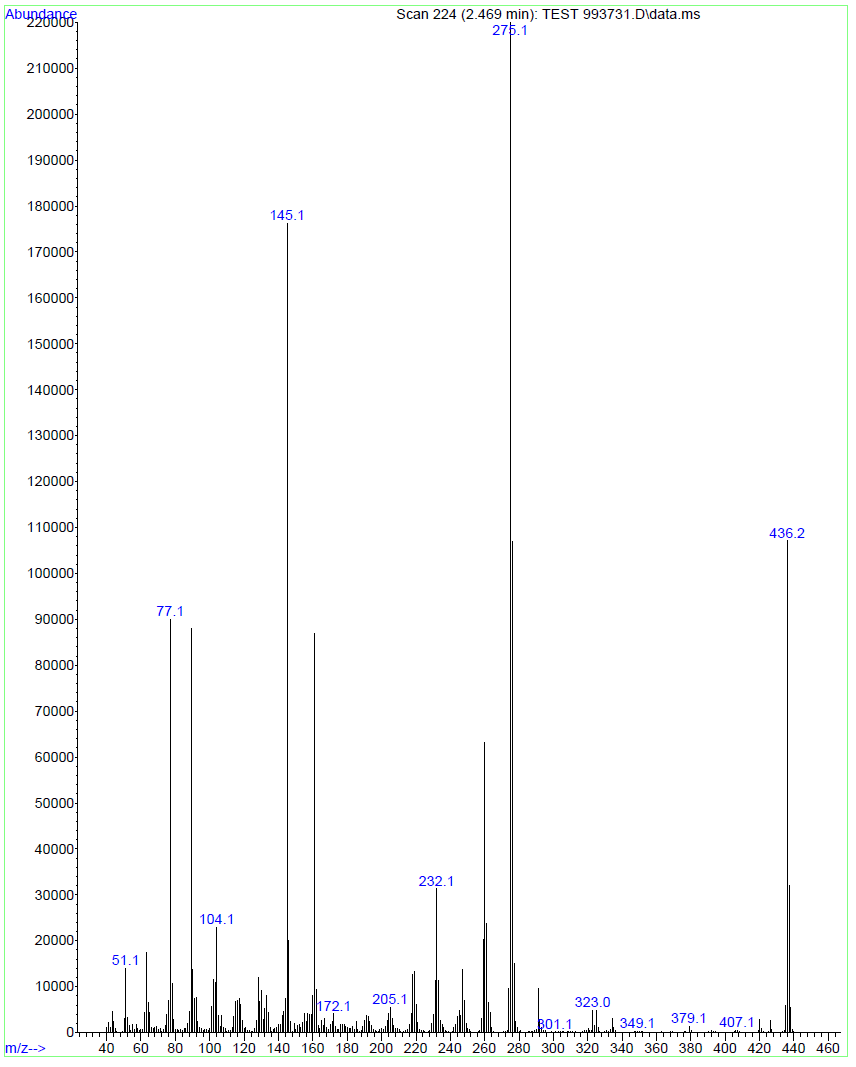


**Fig. S9.** Mass spectra of N'-((3-(4-Methoxyphenyl)-1-phenyl-1H-pyrazol-4-yl)methylene)benzofuran-2-carbohydrazide (8c)


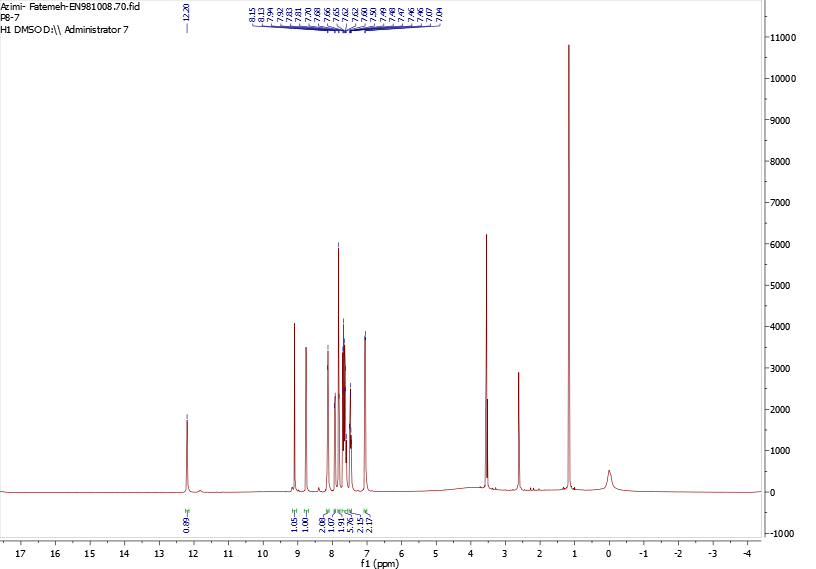


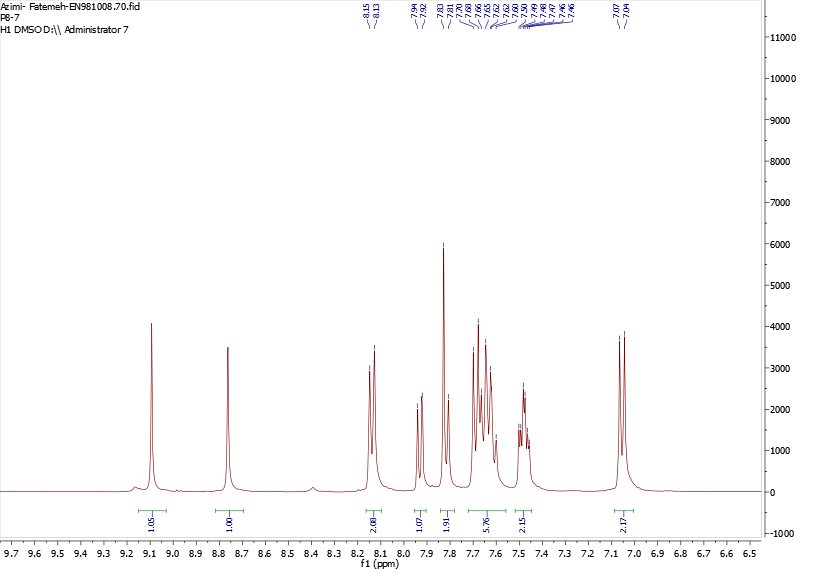


**Fig. S10.** ^1^H NMR spectra of N'-((3-(4-hydroxyphenyl)-1-phenyl-1H-pyrazol-4-yl)methylene)benzofuran-2-carbohydrazide (8d)


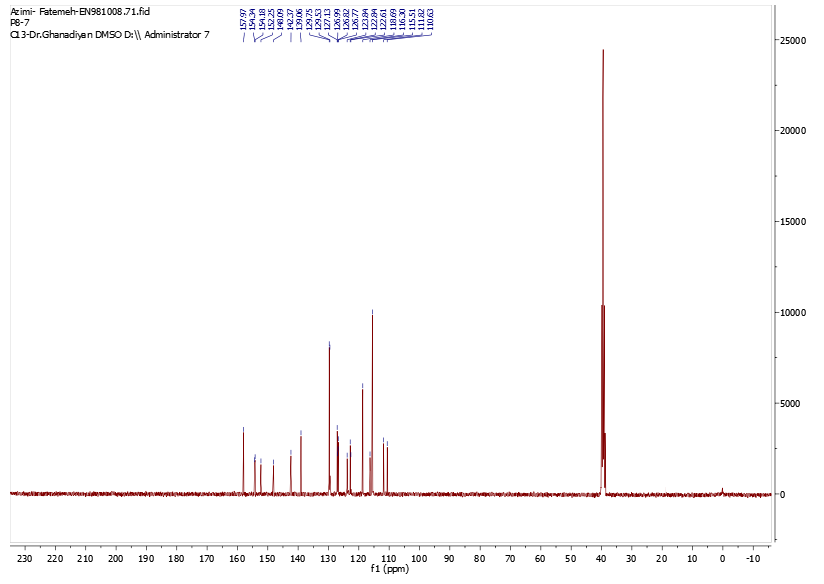


**Fig. S11.** ^13^C NMR spectra of N'-((3-(4-hydroxyphenyl)-1-phenyl-1H-pyrazol-4-yl)methylene)benzofuran-2-carbohydrazide (8d)


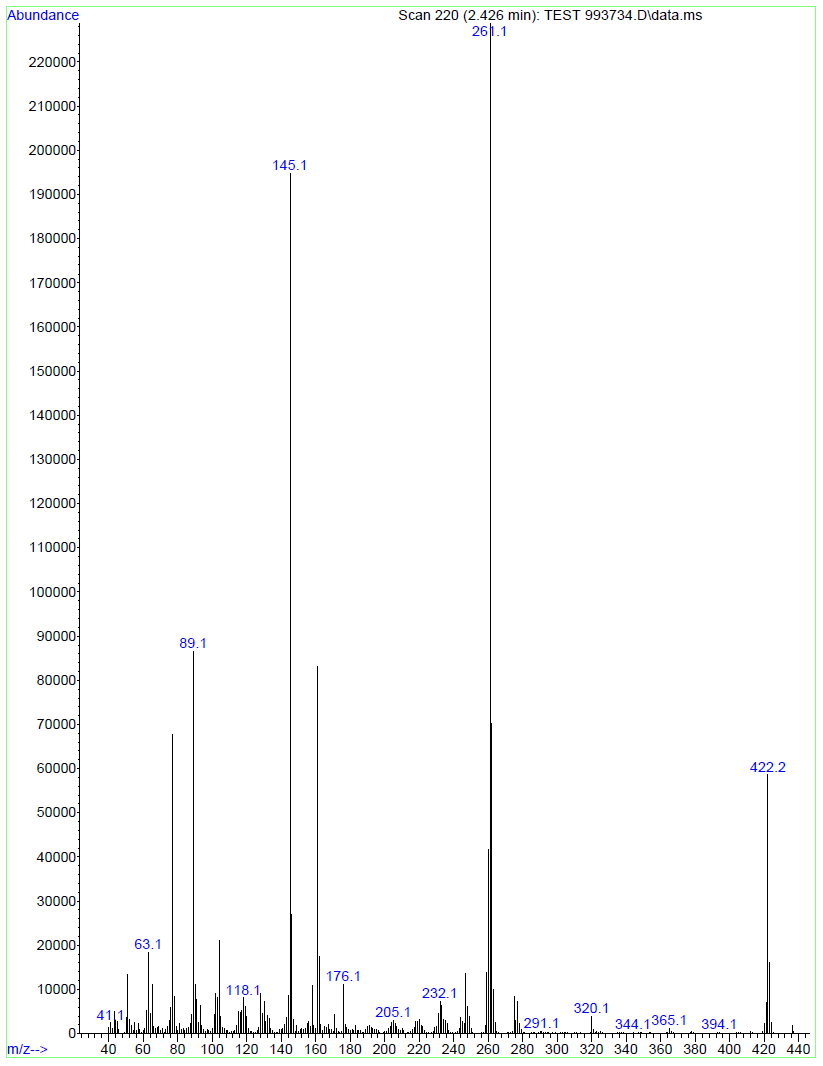


**Fig. S12.** Mass spectra of N'-((3-(4-hydroxyphenyl)-1-phenyl-1H-pyrazol-4-yl)methylene)benzofuran-2-carbohydrazide (8d)


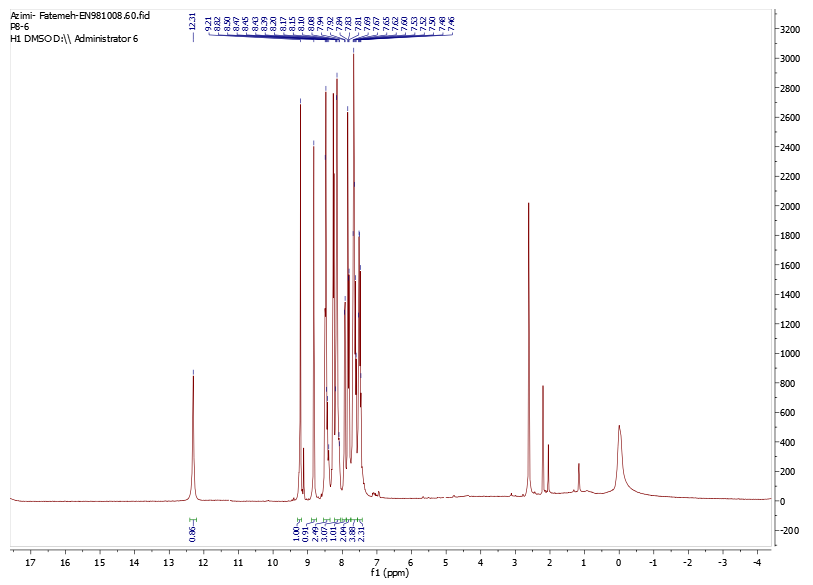


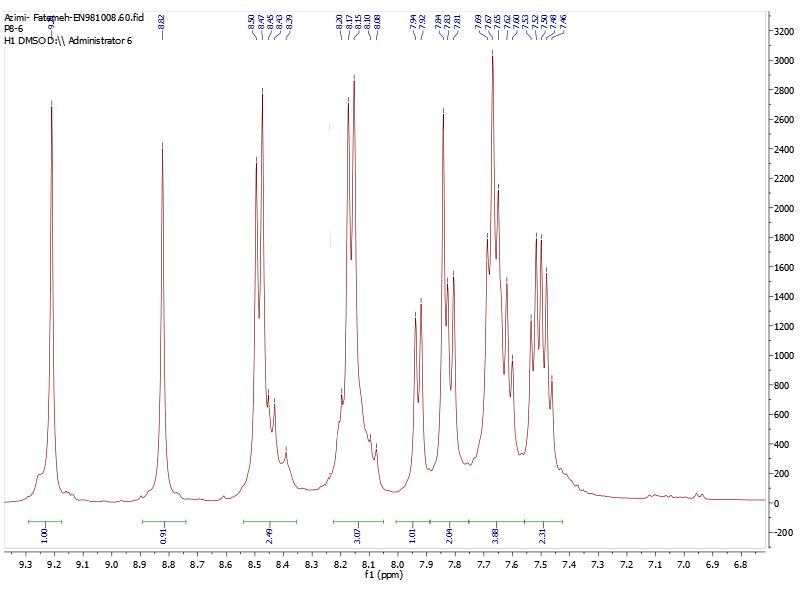


**Fig. S13.** ^1^H NMR spectra of N'-((3-(4-Nitrophenyl)-1-phenyl-1H-pyrazol-4-yl)methylene)benzofuran-2-carbohydrazide (8e)


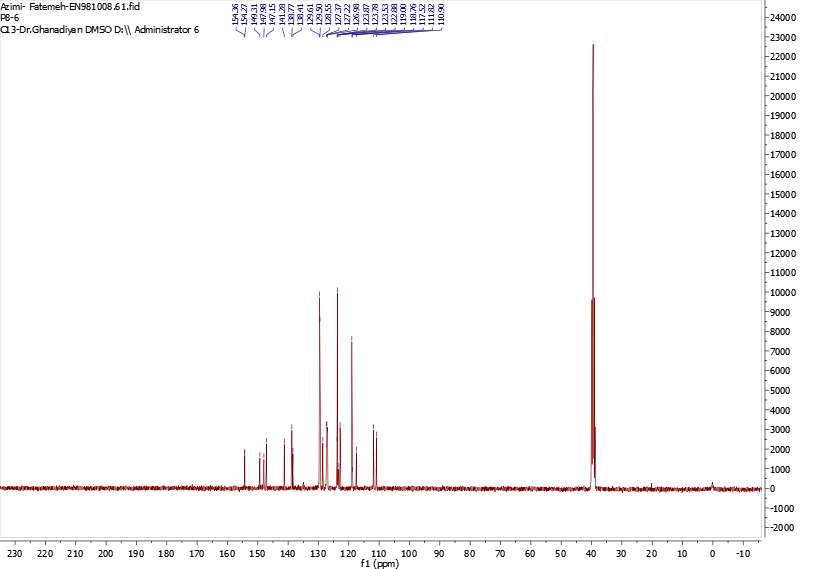


**Fig. S14.** ^13^C NMR spectra of N'-((3-(4-Nitrophenyl)-1-phenyl-1H-pyrazol-4-yl)methylene)benzofuran-2-carbohydrazide (8e)


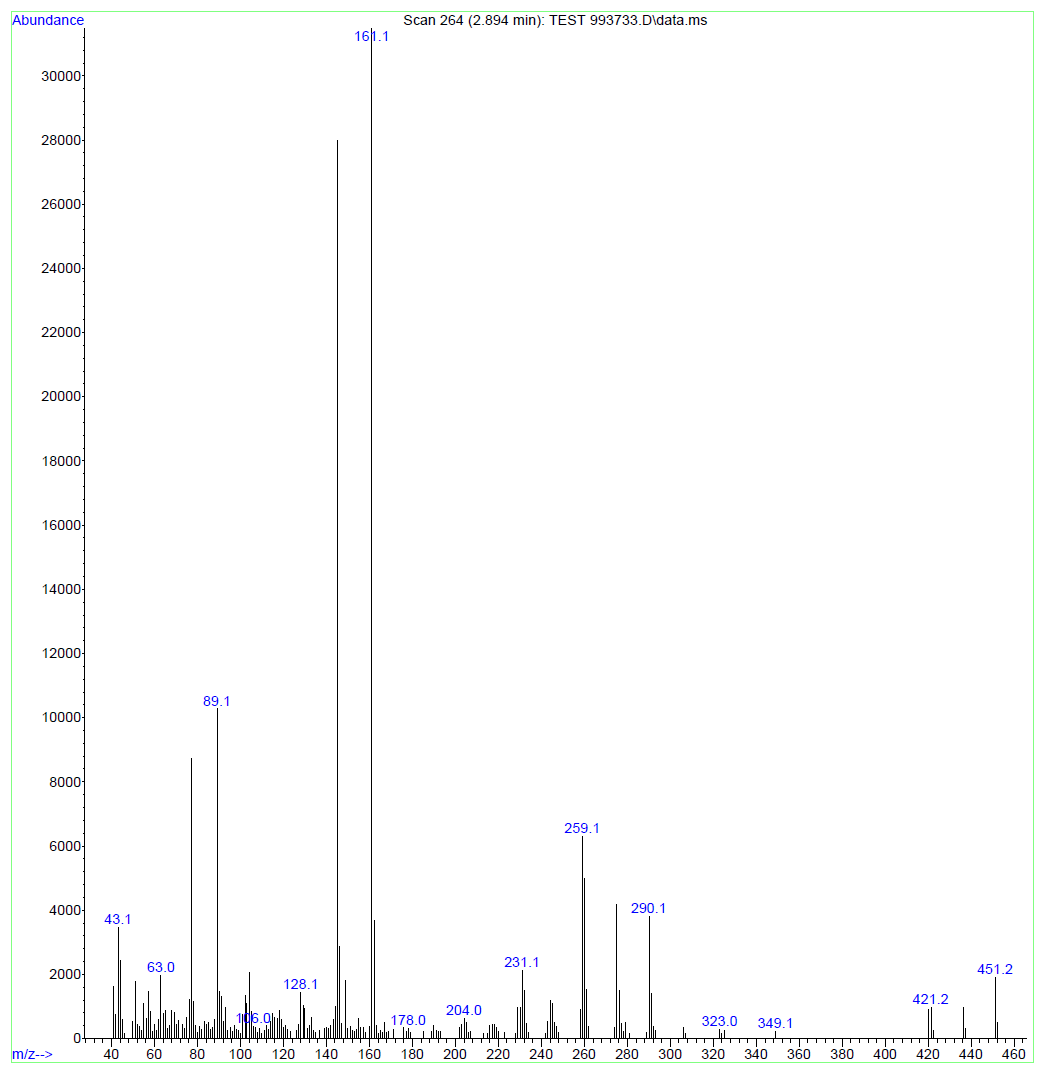


**Fig. S15.** Mass spectra of N'-((3-(4-Nitrophenyl)-1-phenyl-1H-pyrazol-4-yl)methylene)benzofuran-2-carbohydrazide (8e)


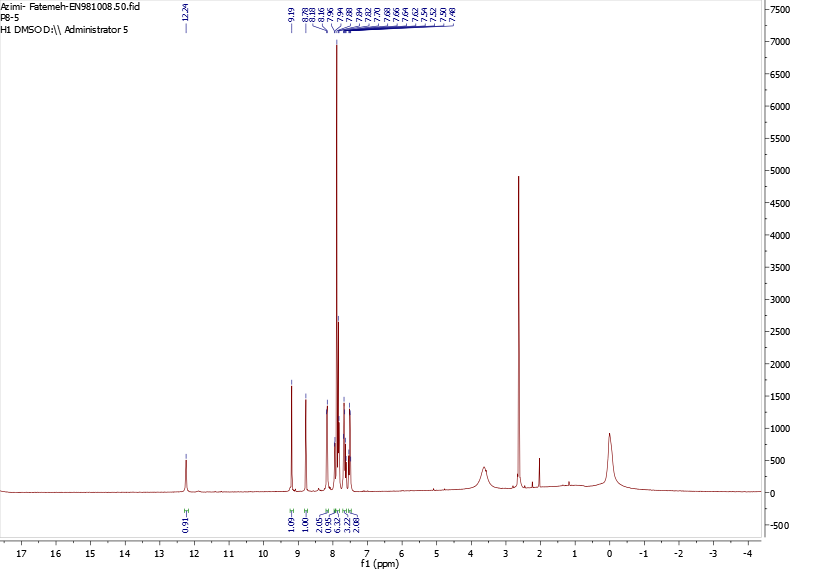


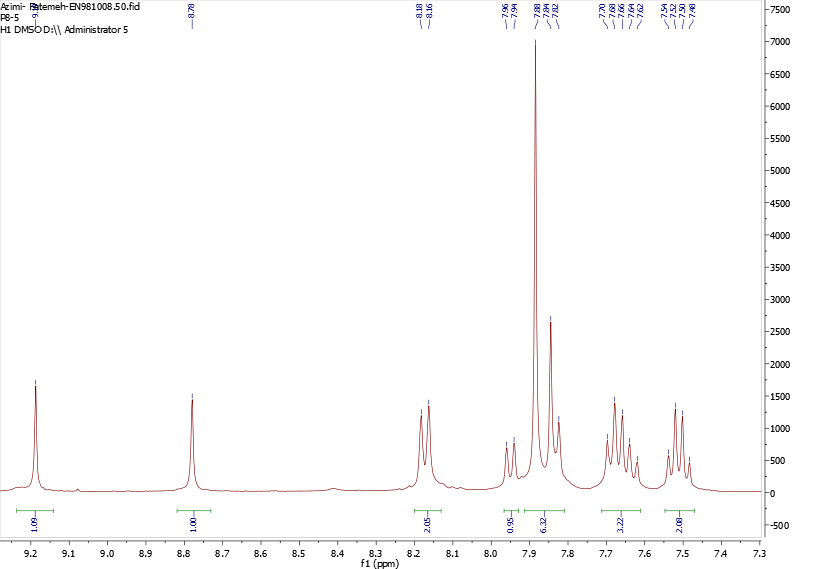


**Fig. S16.** ^1^H NMR spectra of N'-((3-(4-Bromophenyl)-1-phenyl-1H-pyrazol-4-yl)methylene)benzofuran-2-carbohydrazide (8f)


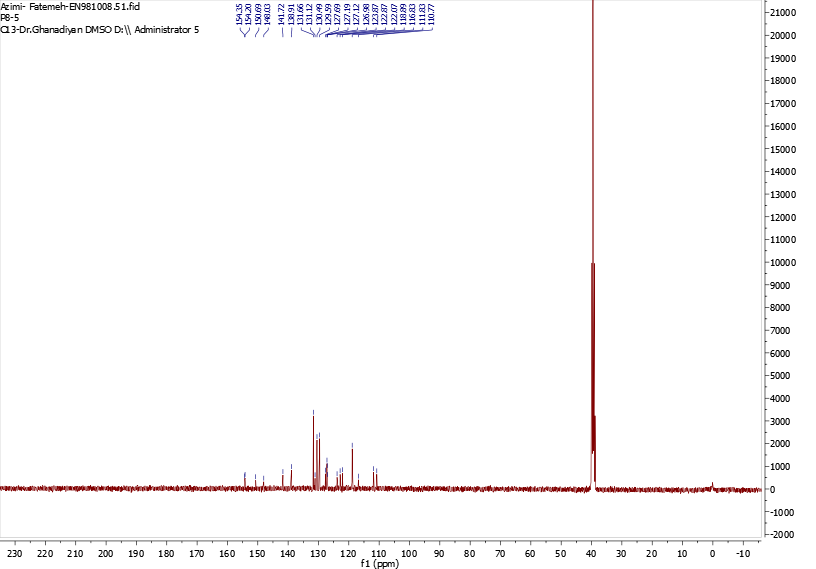


**Fig. S17.** ^13^C NMR spectra of N'-((3-(4-Bromophenyl)-1-phenyl-1H-pyrazol-4-yl)methylene)benzofuran-2-carbohydrazide (8f)


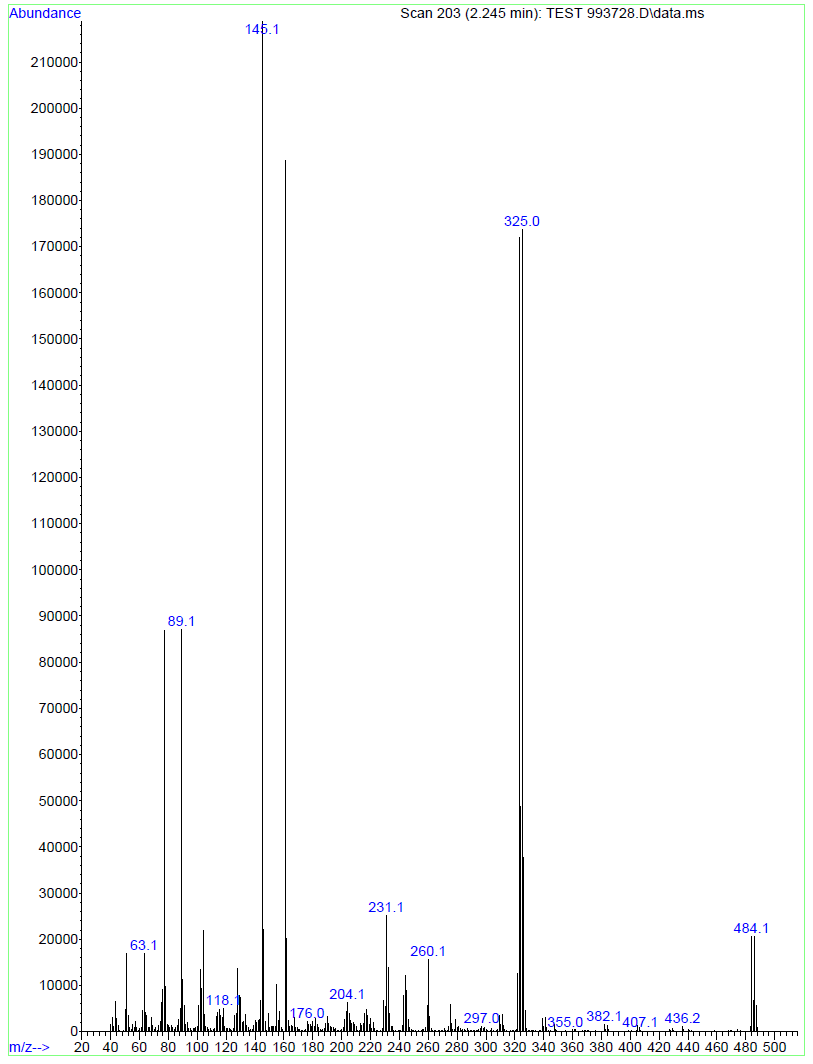


**Fig. S18.** Mass spectra of N'-((3-(4-Bromophenyl)-1-phenyl-1H-pyrazol-4-yl)methylene)benzofuran-2-carbohydrazide (8f)


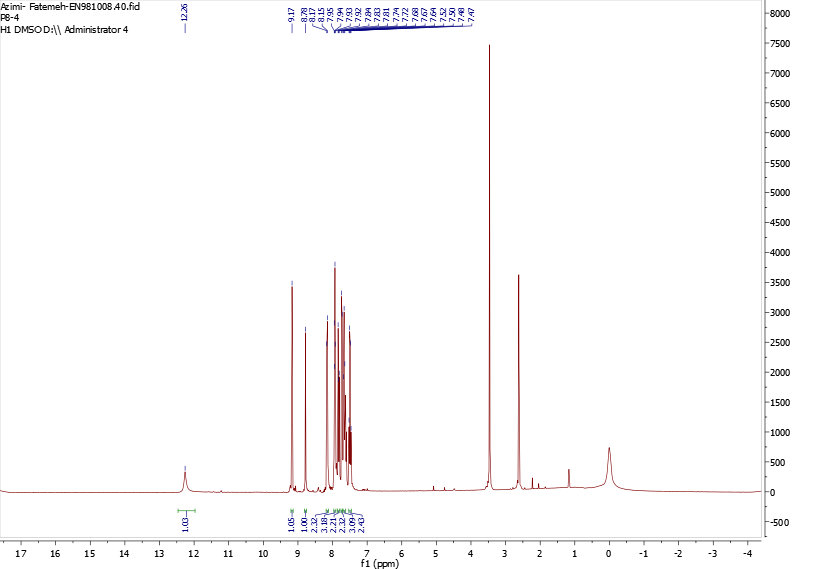


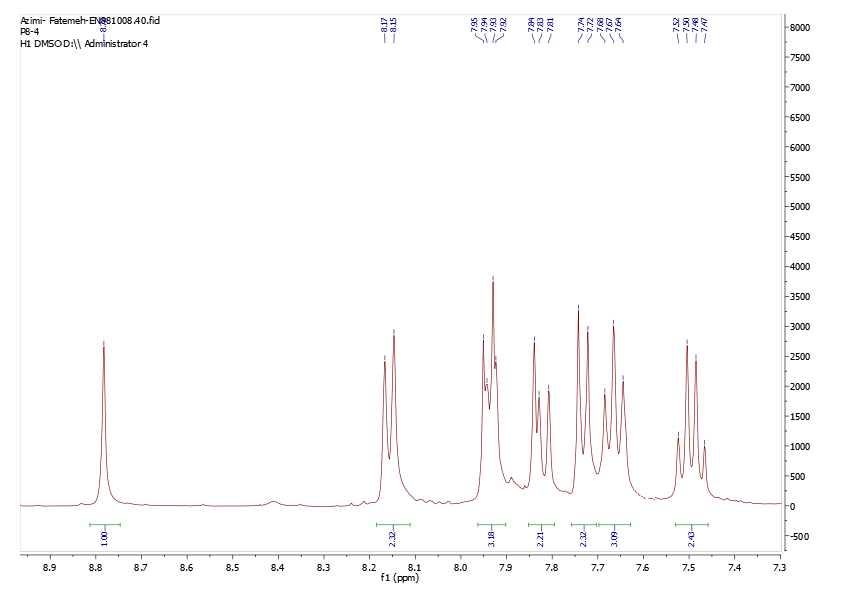


**Fig. S19.** ^1^H NMR spectra of N'-((3-(4-Chlorophenyl)-1-phenyl-1H-pyrazol-4-yl)methylene)benzofuran-2-carbohydrazide (8g)


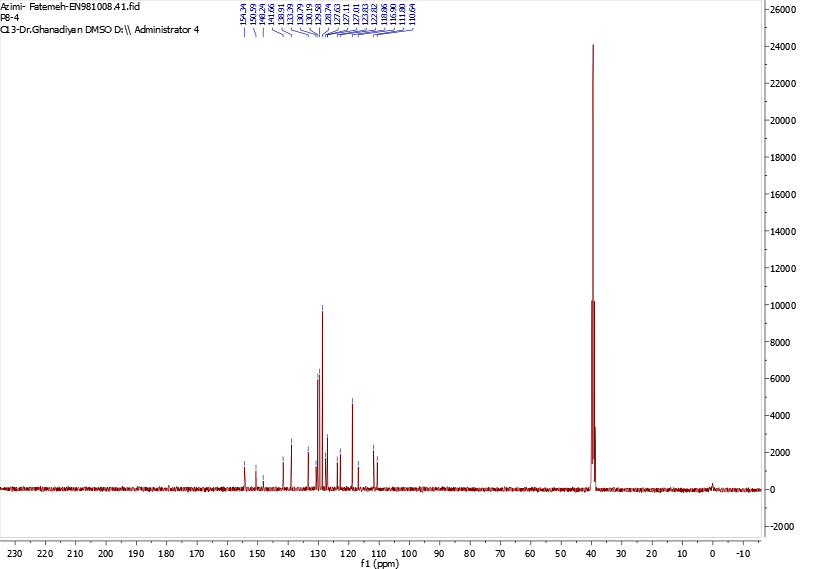


**Fig. S20.** ^13^C NMR spectra of N'-((3-(4-Chlorophenyl)-1-phenyl-1H-pyrazol-4-yl)methylene)benzofuran-2-carbohydrazide (8g)


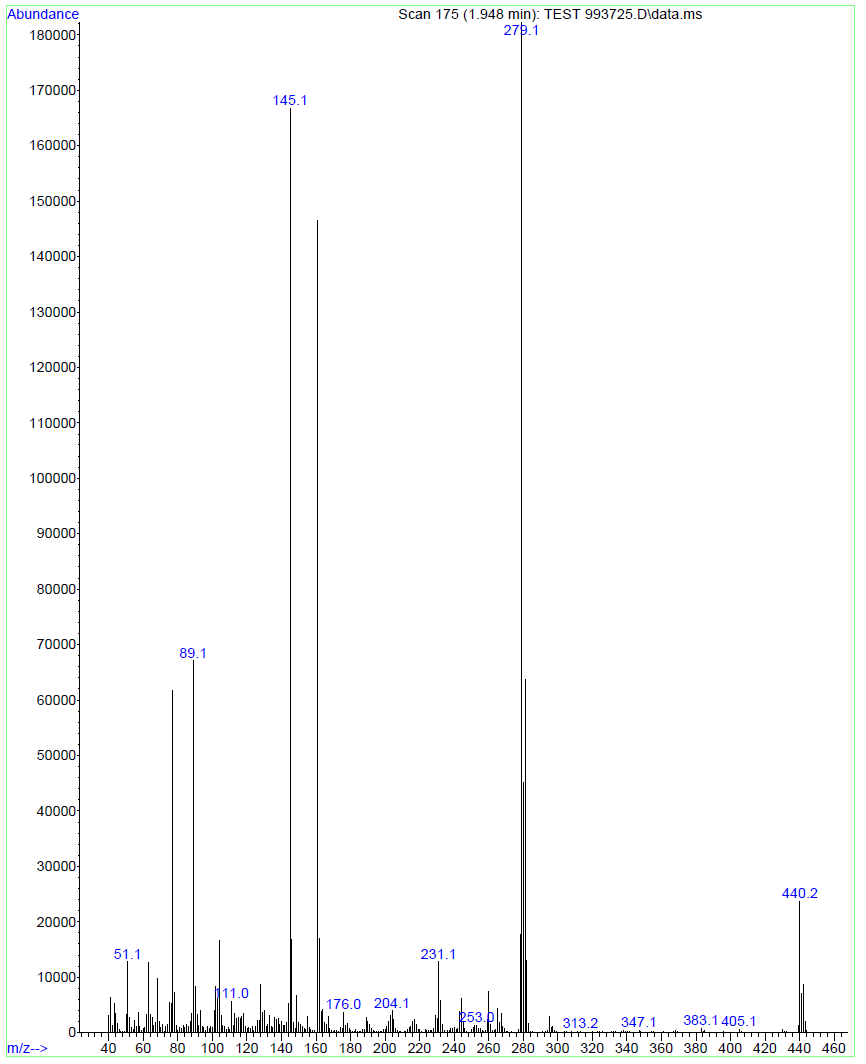


**Fig. S21.** Mass spectra of N'-((3-(4-Chlorophenyl)-1-phenyl-1H-pyrazol-4-yl)methylene)benzofuran-2-carbohydrazide (8g)


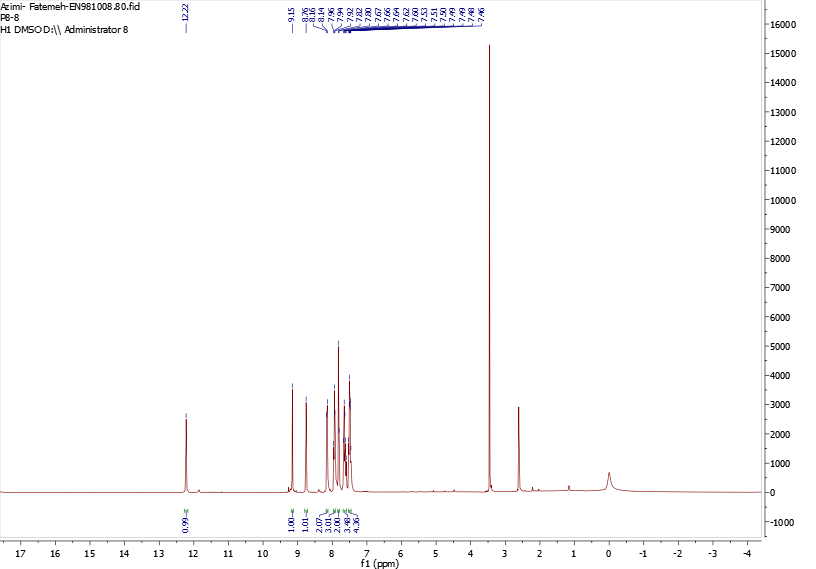


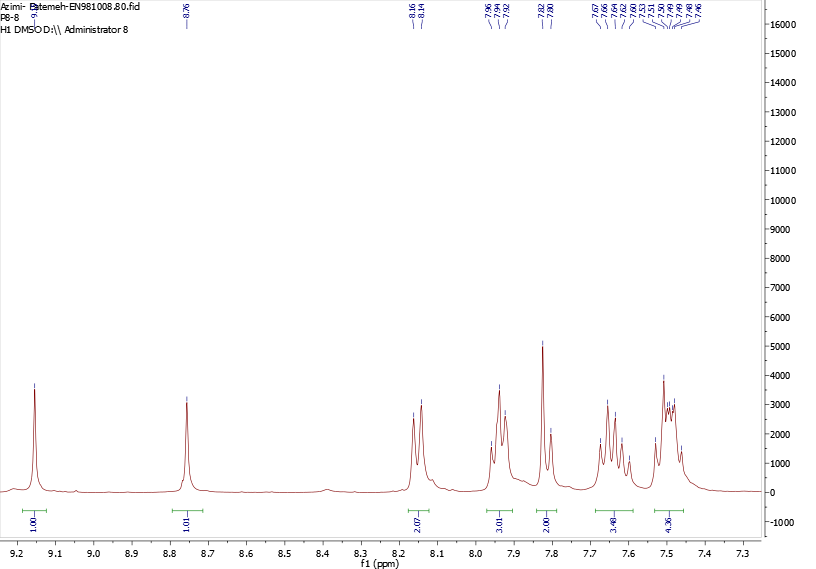


**Fig. S22.** ^1^H NMR spectra of N'-((3-(4-Fluorophenyl)-1-phenyl-1H-pyrazol-4-yl)methylene)benzofuran-2-carbohydrazide (8h)


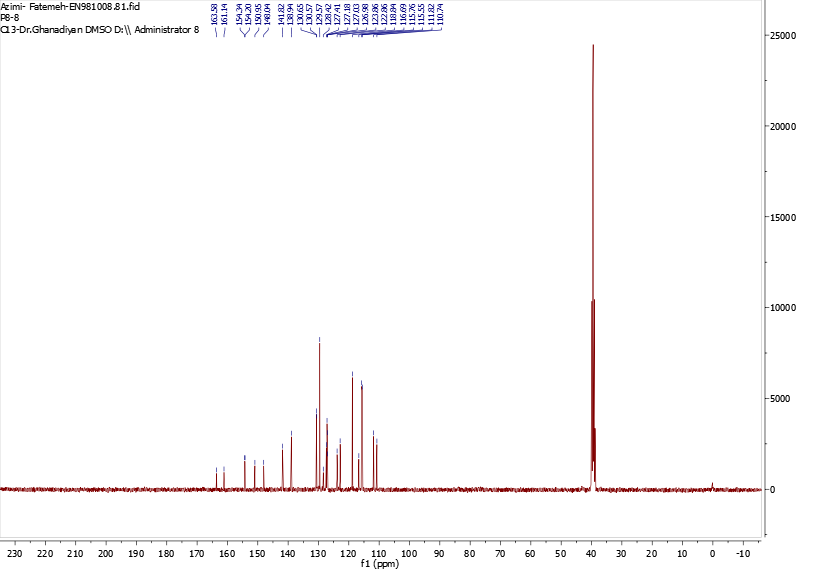


**Fig. S23.** ^13^C NMR spectra of N'-((3-(4-Fluorophenyl)-1-phenyl-1H-pyrazol-4-yl)methylene)benzofuran-2-carbohydrazide (8h)


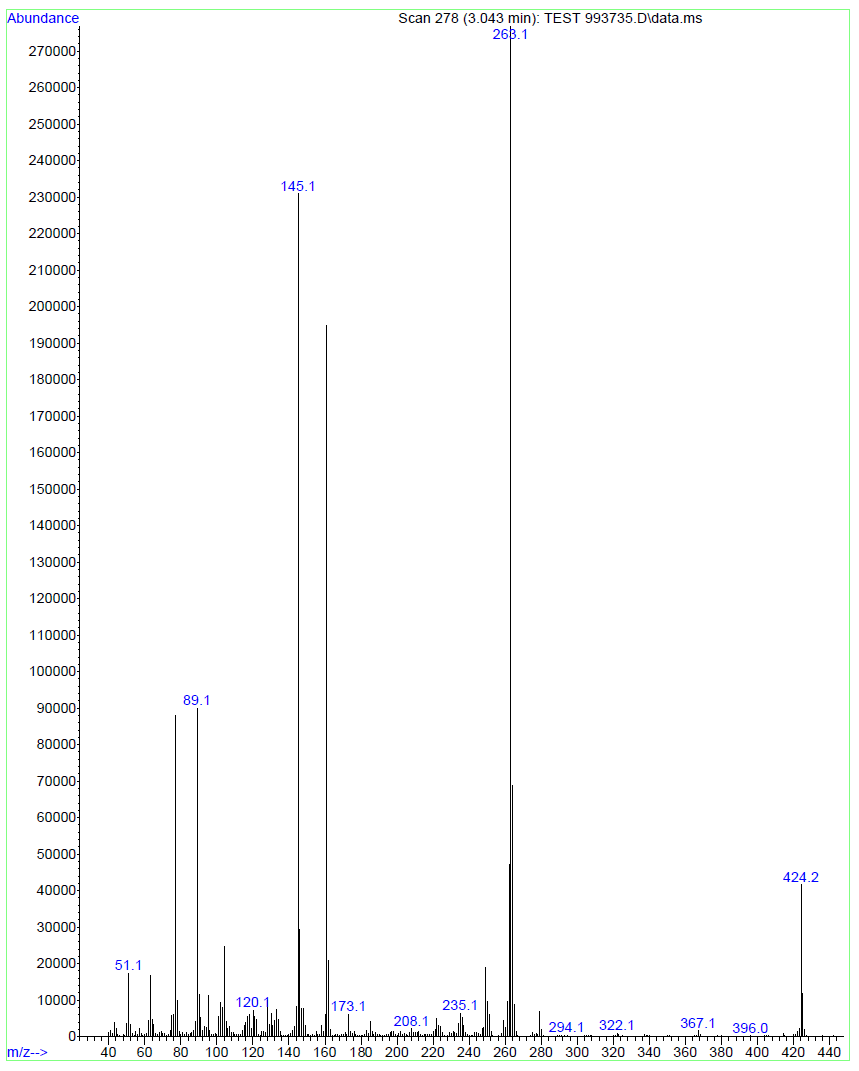


**Fig. S24.** Mass spectra of N'-((3-(4-Fluorophenyl)-1-phenyl-1H-pyrazol-4-yl)methylene)benzofuran-2-carbohydrazide (8h)


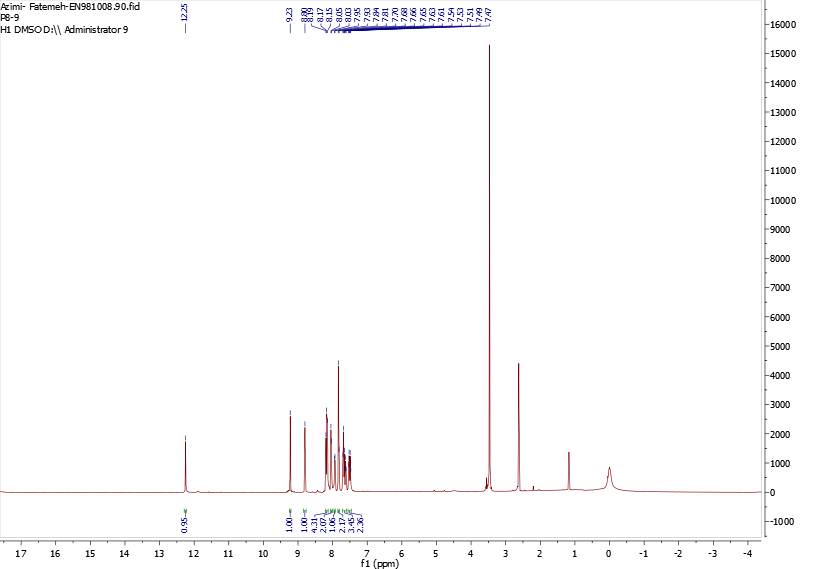


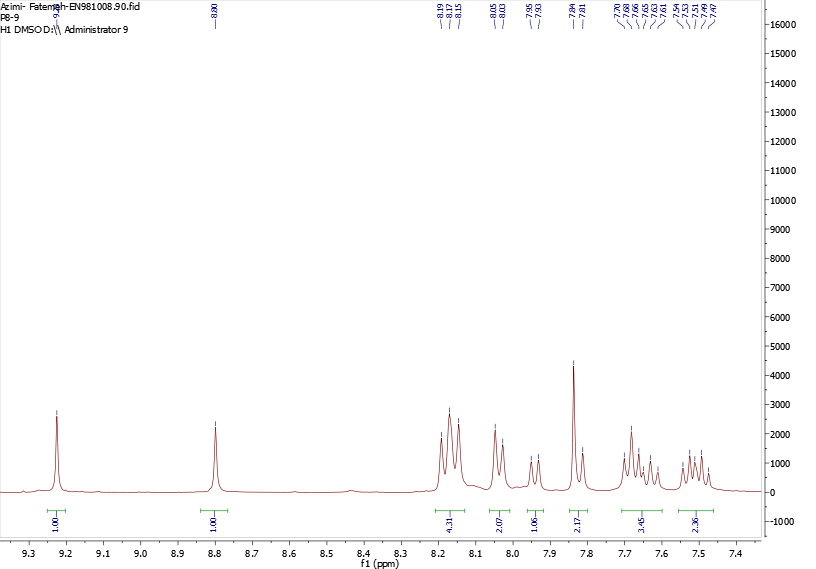


**Fig. S25.** ^1^H NMR spectra of N'-((1-Phenyl-3-(4-(trifluoromethyl)phenyl)-1H-pyrazol-4-yl)methylene)benzofuran-2-carbohydrazide (8i)


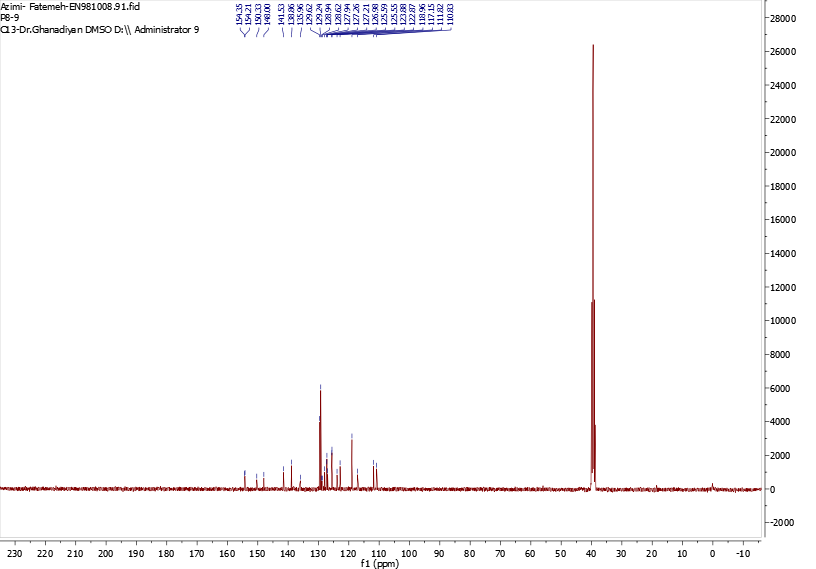


**Fig. S26.** ^13^C NMR spectra of N'-((1-Phenyl-3-(4-(trifluoromethyl)phenyl)-1H-pyrazol-4-yl)methylene)benzofuran-2-carbohydrazide (8i)


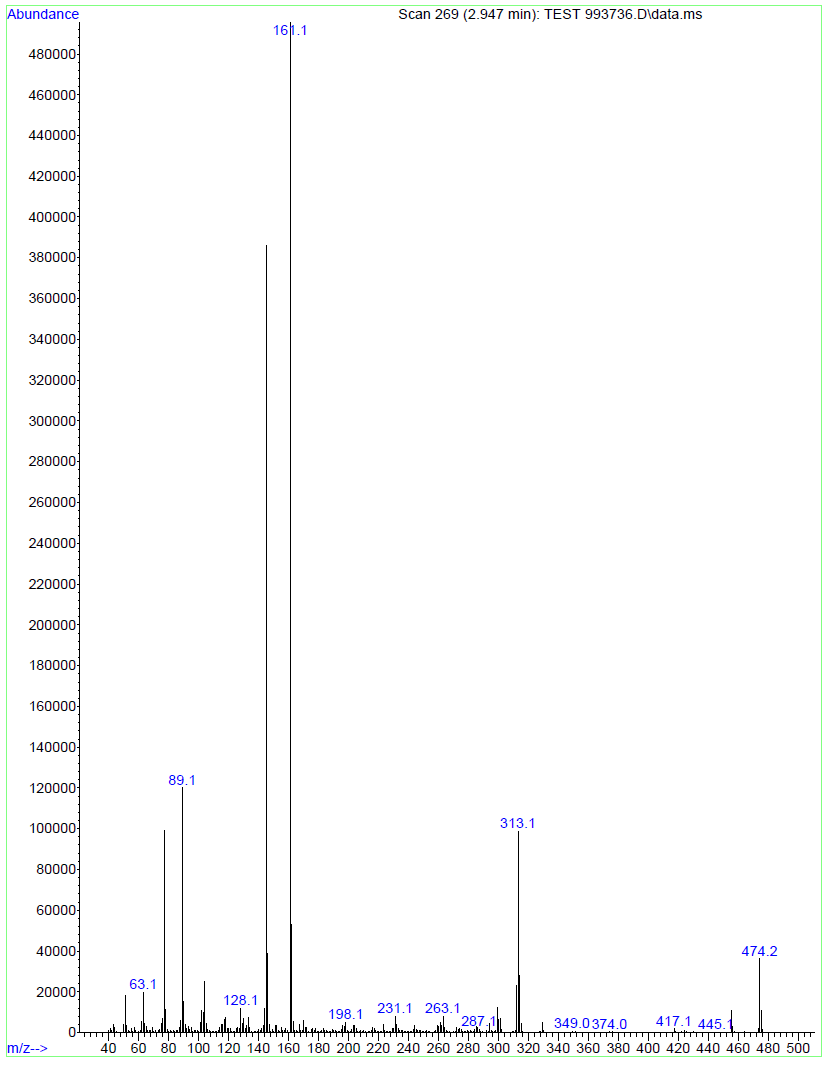


**Fig. S27.** Mass spectra of N'-((1-Phenyl-3-(4-(trifluoromethyl)phenyl)-1H-pyrazol-4-yl)methylene)benzofuran-2-carbohydrazide (8i)


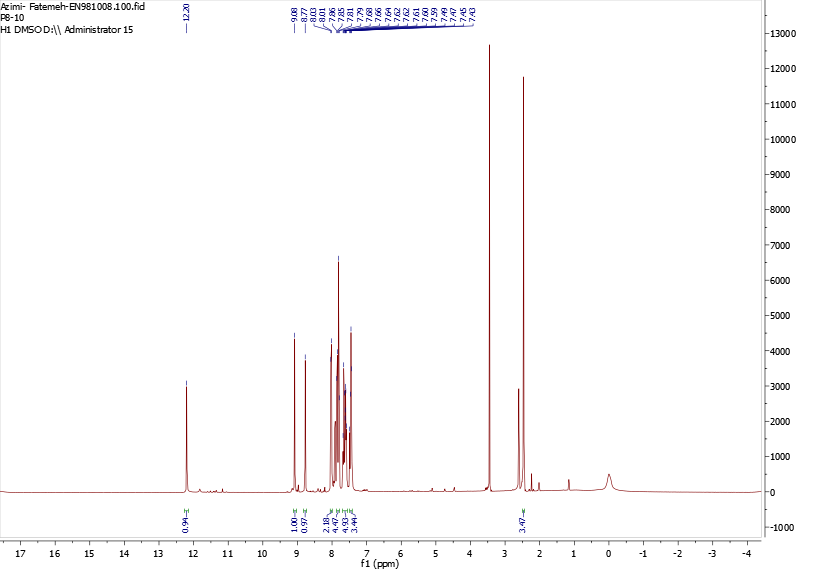


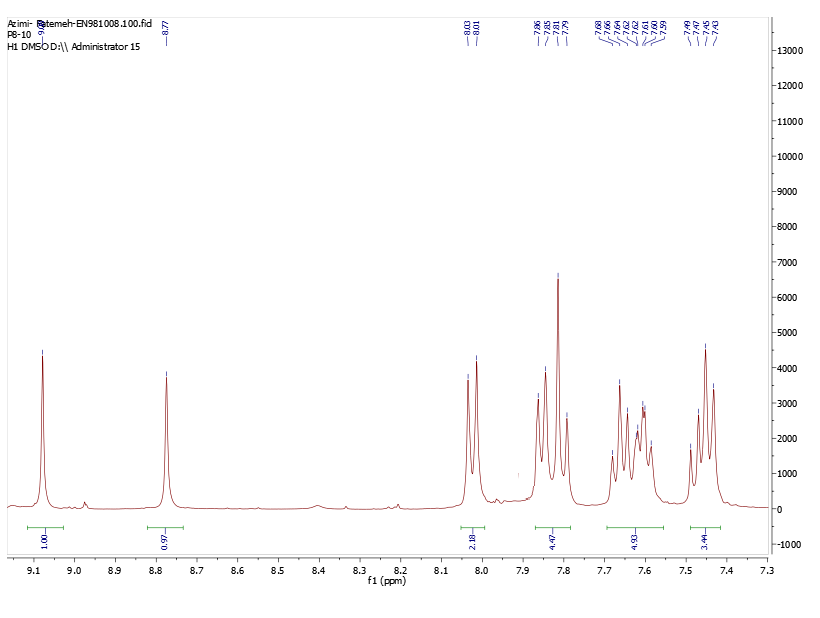


**Fig. S28.** ^1^H NMR spectra of N'-((3-Phenyl-1-p-tolyl-1H-pyrazol-4-yl)methylene)benzofuran-2-carbohydrazide (8j)


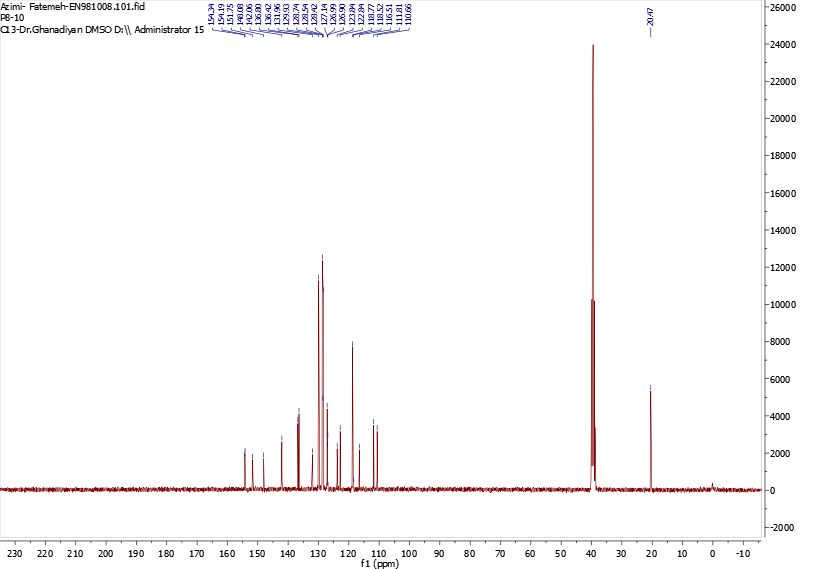


**Fig. S29.** ^13^C NMR spectra of N'-((3-Phenyl-1-p-tolyl-1H-pyrazol-4-yl)methylene)benzofuran-2-carbohydrazide (8j)


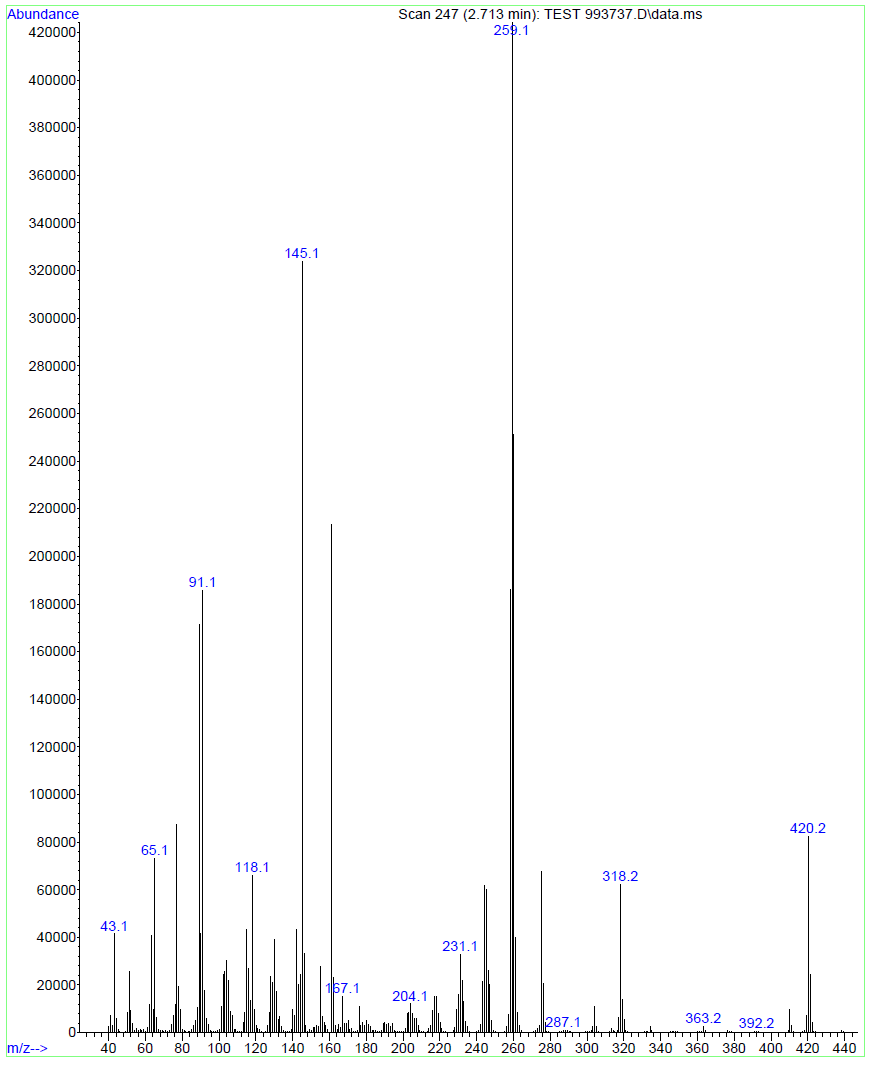


**Fig. S30.** Mass spectra of N'-((3-Phenyl-1-p-tolyl-1H-pyrazol-4-yl)methylene)benzofuran-2-carbohydrazide (8j)


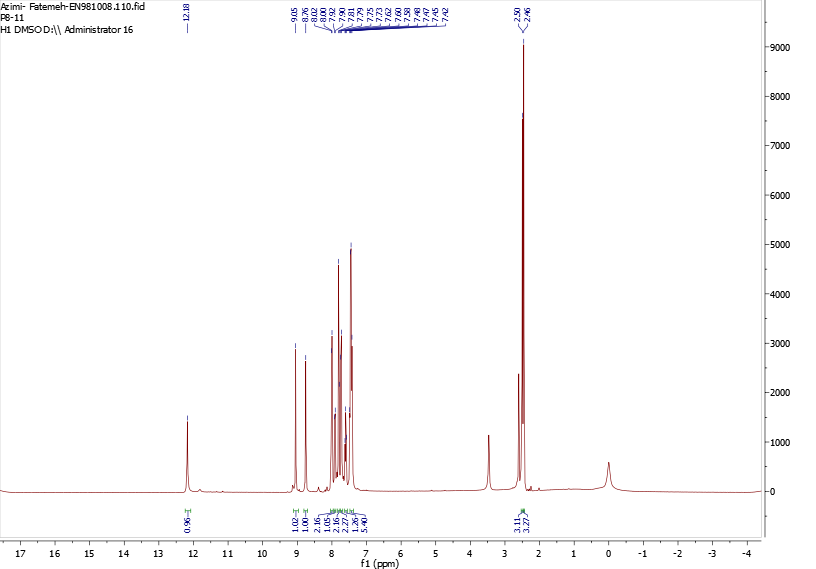


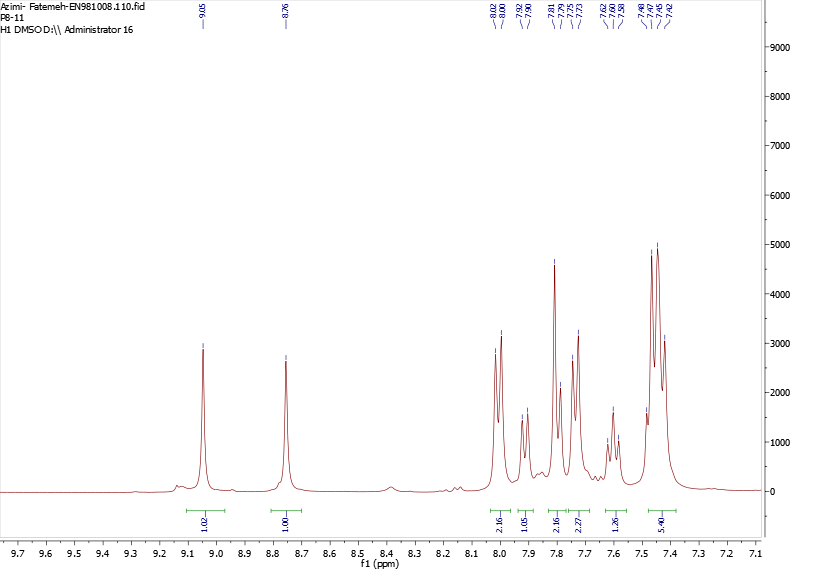


**Fig. S31.** ^1^H NMR spectra of N'-((1,3-Dip-tolyl-1H-pyrazol-4-yl)methylene)benzofuran-2-carbohydrazide (8k)


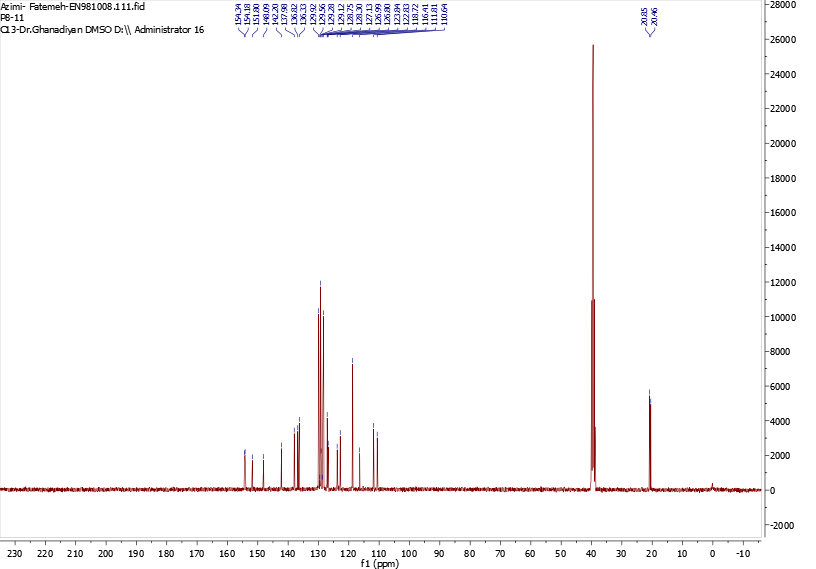


**Fig. S32.** ^13^C NMR spectra of N'-((1,3-Dip-tolyl-1H-pyrazol-4-yl)methylene)benzofuran-2-carbohydrazide (8k)


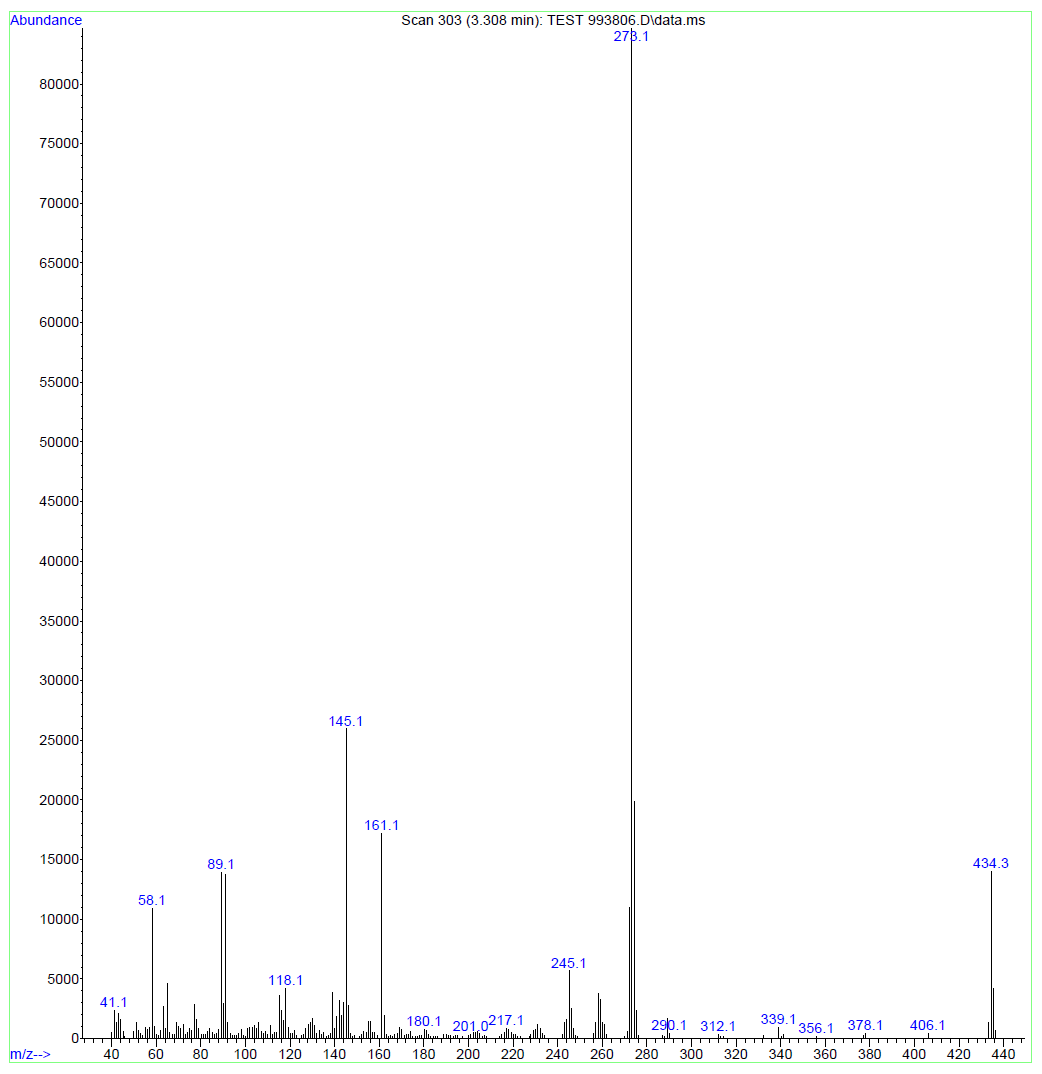


**Fig. S33.** Mass spectra of N'-((1,3-Dip-tolyl-1H-pyrazol-4-yl)methylene)benzofuran-2-carbohydrazide (8k)


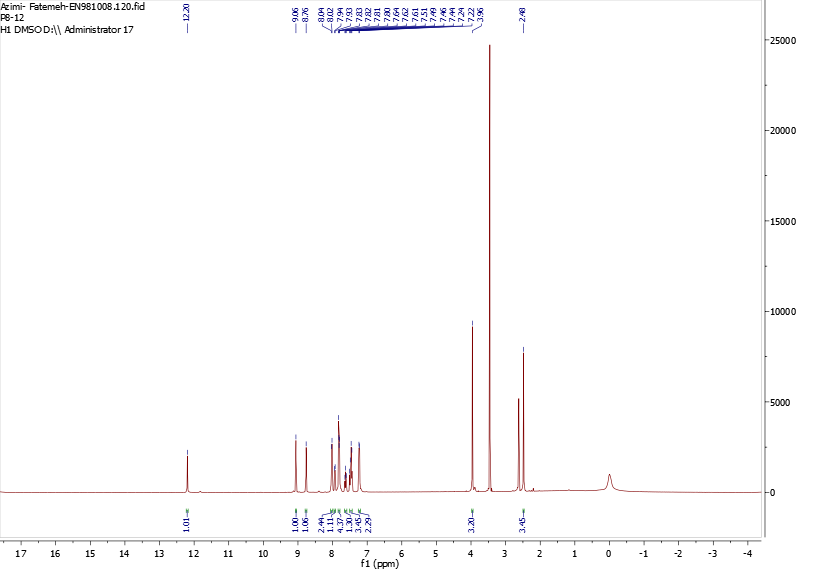


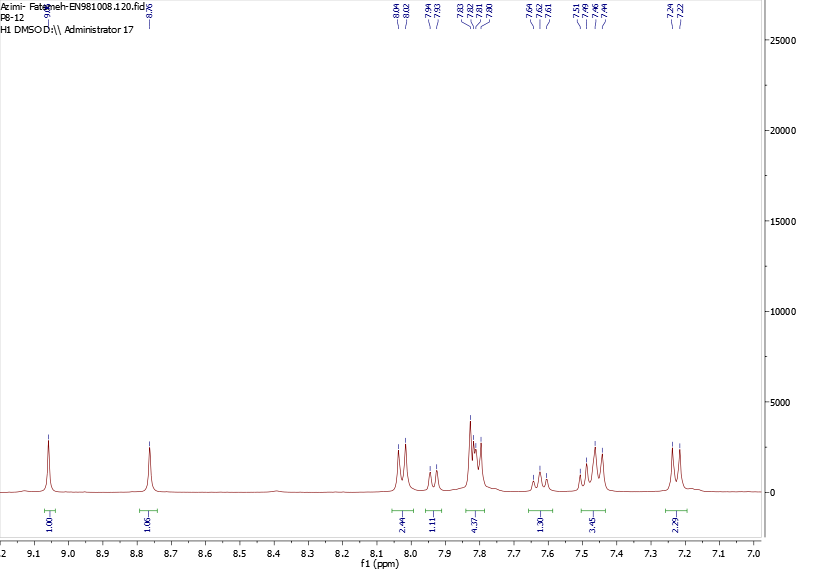


**Fig. S34.** ^1^H NMR spectra of N'-((3-(4-Methoxyphenyl)-1-p-tolyl-1H-pyrazol-4-yl)methylene)benzofuran-2-carbohydrazide (8l)


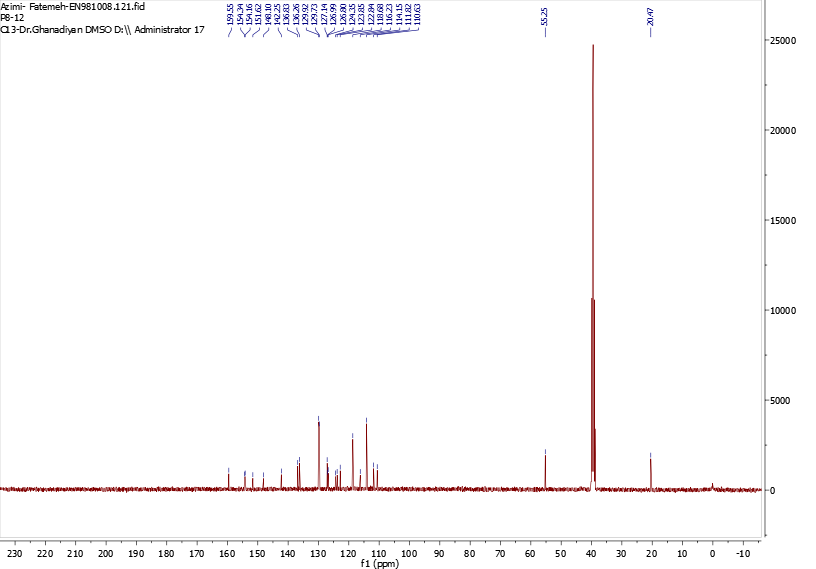


**Fig. S35.** ^13^C NMR spectra of N'-((3-(4-Methoxyphenyl)-1-p-tolyl-1H-pyrazol-4-yl)methylene)benzofuran-2-carbohydrazide (8l)


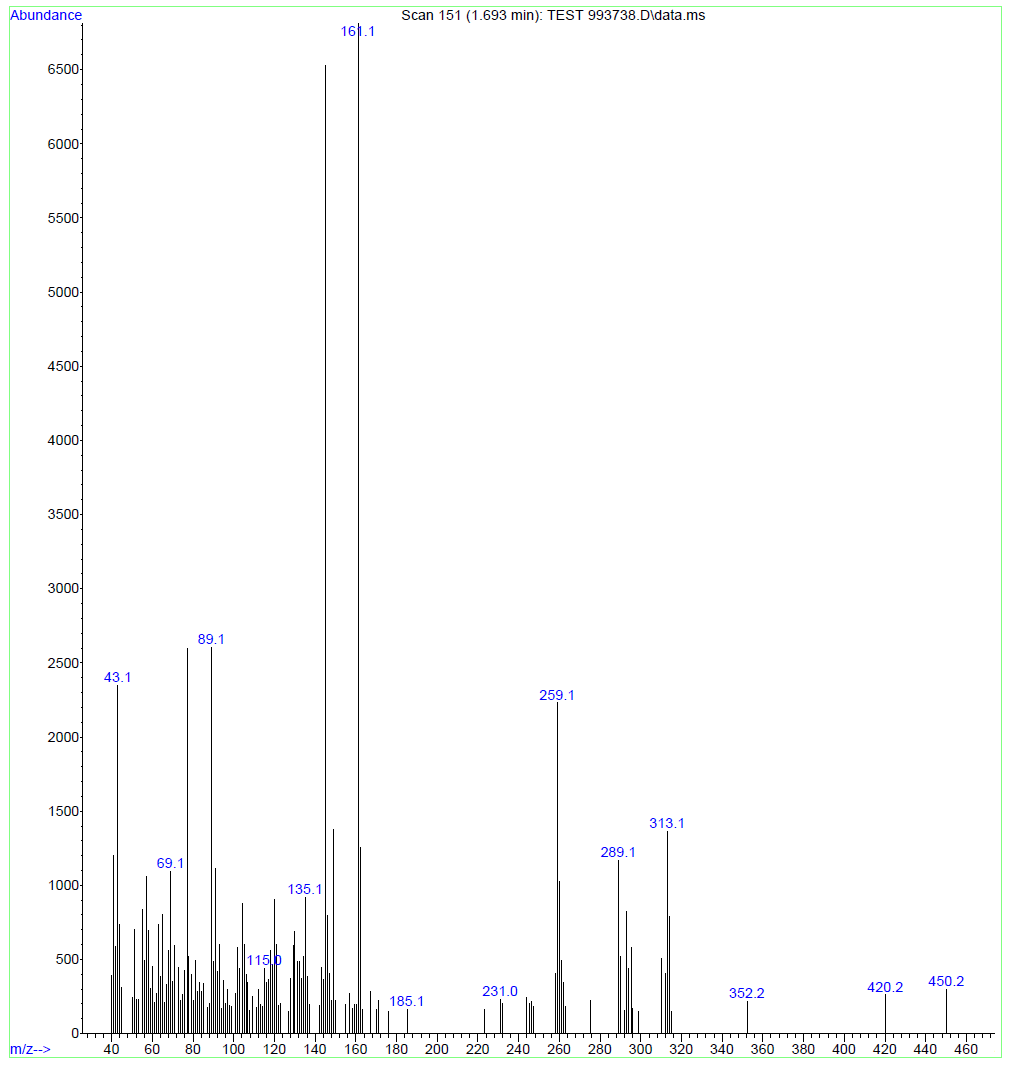


**Fig. S36.** Mass spectra of N'-((3-(4-Methoxyphenyl)-1-p-tolyl-1H-pyrazol-4-yl)methylene)benzofuran-2-carbohydrazide (8l)


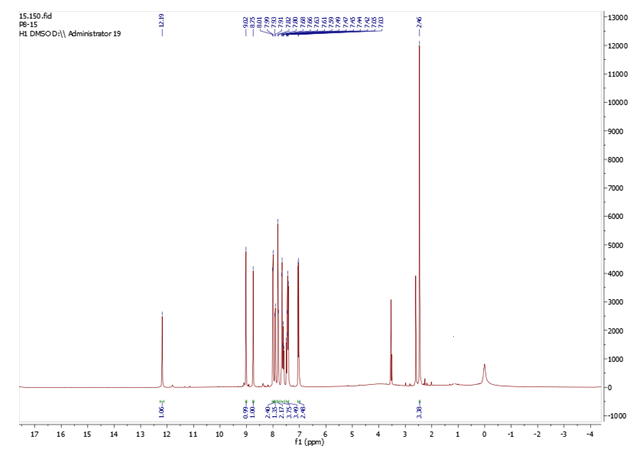


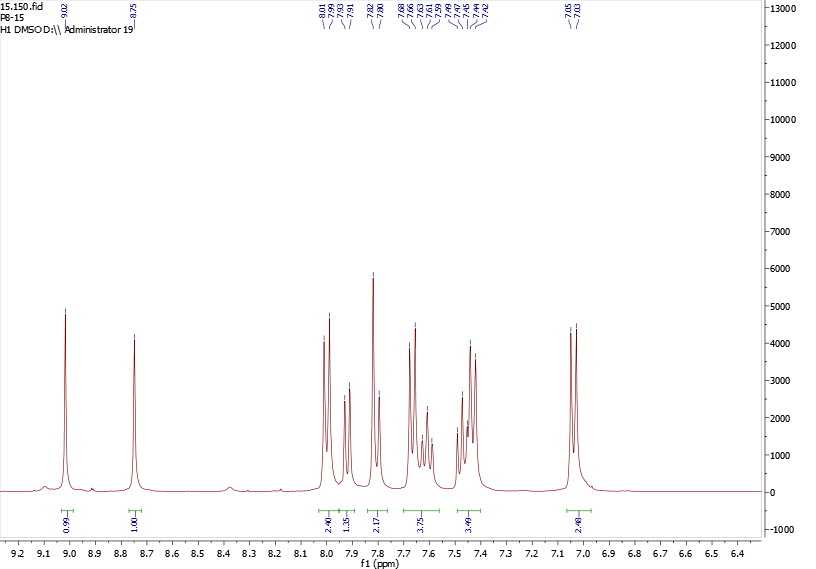


**Fig. S37.** ^1^H NMR spectra of N'-((3-(4-Hydroxyphenyl)-1-p-tolyl-1H-pyrazol-4-yl)methylene)benzofuran-2-carbohydrazide (8m)


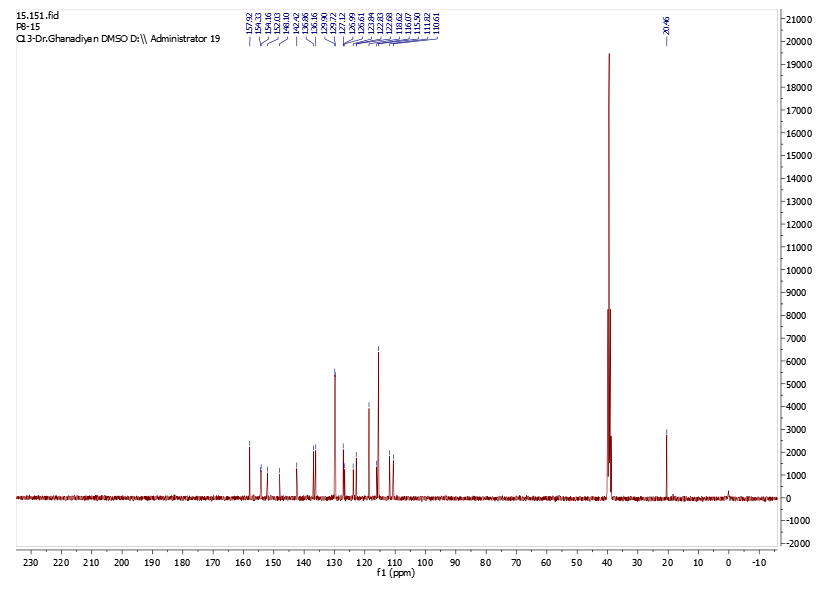


**Fig. S38.** ^13^C NMR spectra of N'-((3-(4-Hydroxyphenyl)-1-p-tolyl-1H-pyrazol-4-yl)methylene)benzofuran-2-carbohydrazide (8m)


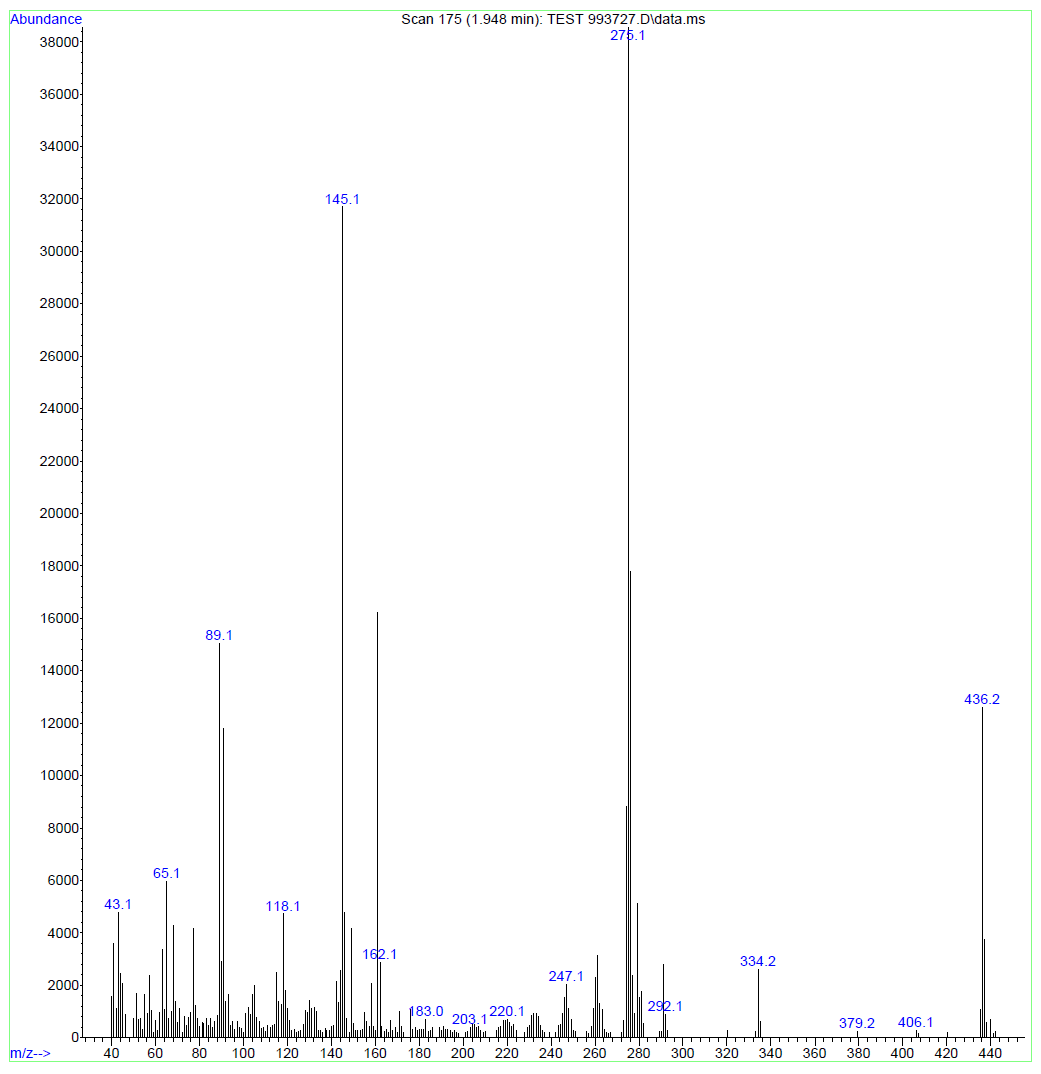


**Fig. S39.** Mass spectra of N'-((3-(4-Hydroxyphenyl)-1-p-tolyl-1H-pyrazol-4-yl)methylene)benzofuran-2-carbohydrazide (8m)


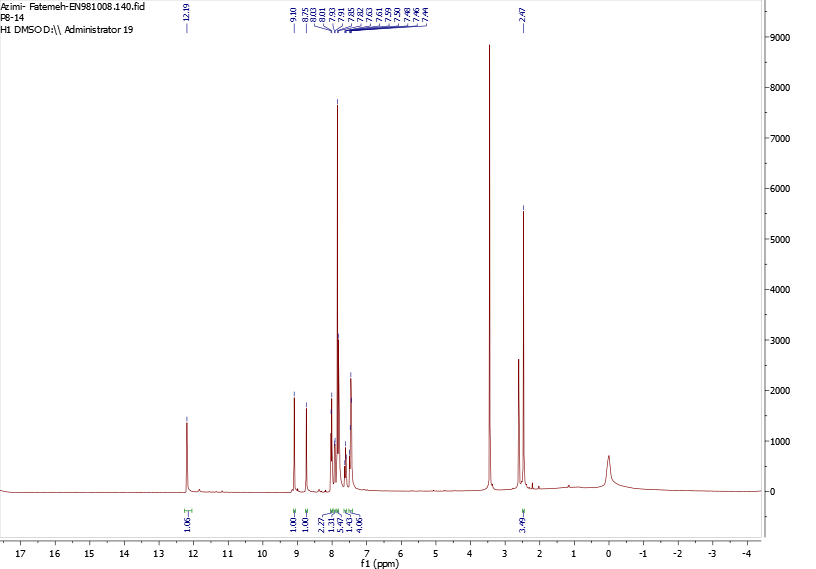


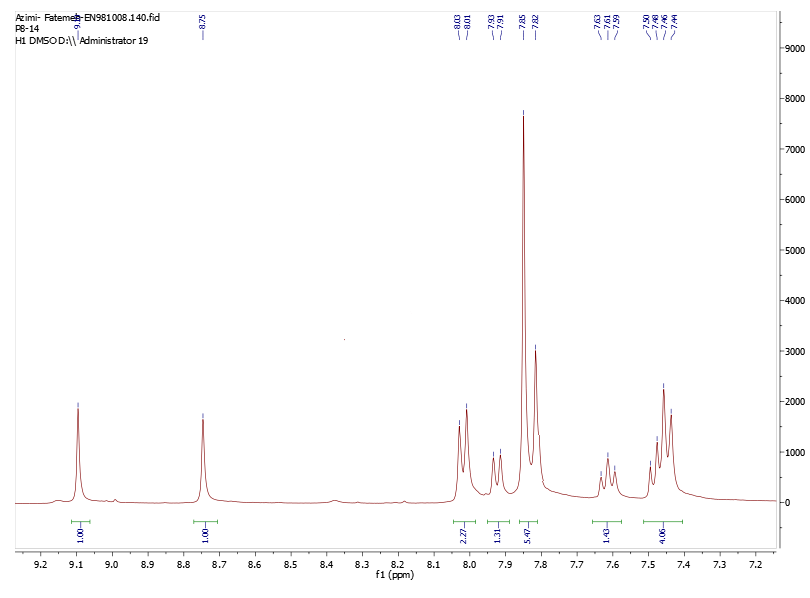


**Fig. S40.** ^1^H NMR spectra of N'-((3-(4-Bromophenyl)-1-p-tolyl-1H-pyrazol-4-yl)methylene)benzofuran-2-carbohydrazide (8n)


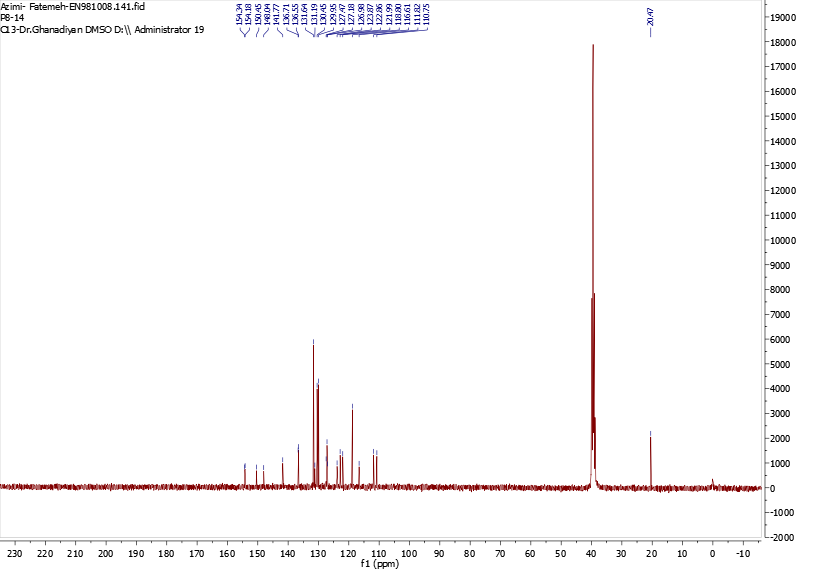


**Fig. S41.** ^13^C NMR spectra of N'-((3-(4-Bromophenyl)-1-p-tolyl-1H-pyrazol-4-yl)methylene)benzofuran-2-carbohydrazide (8n)


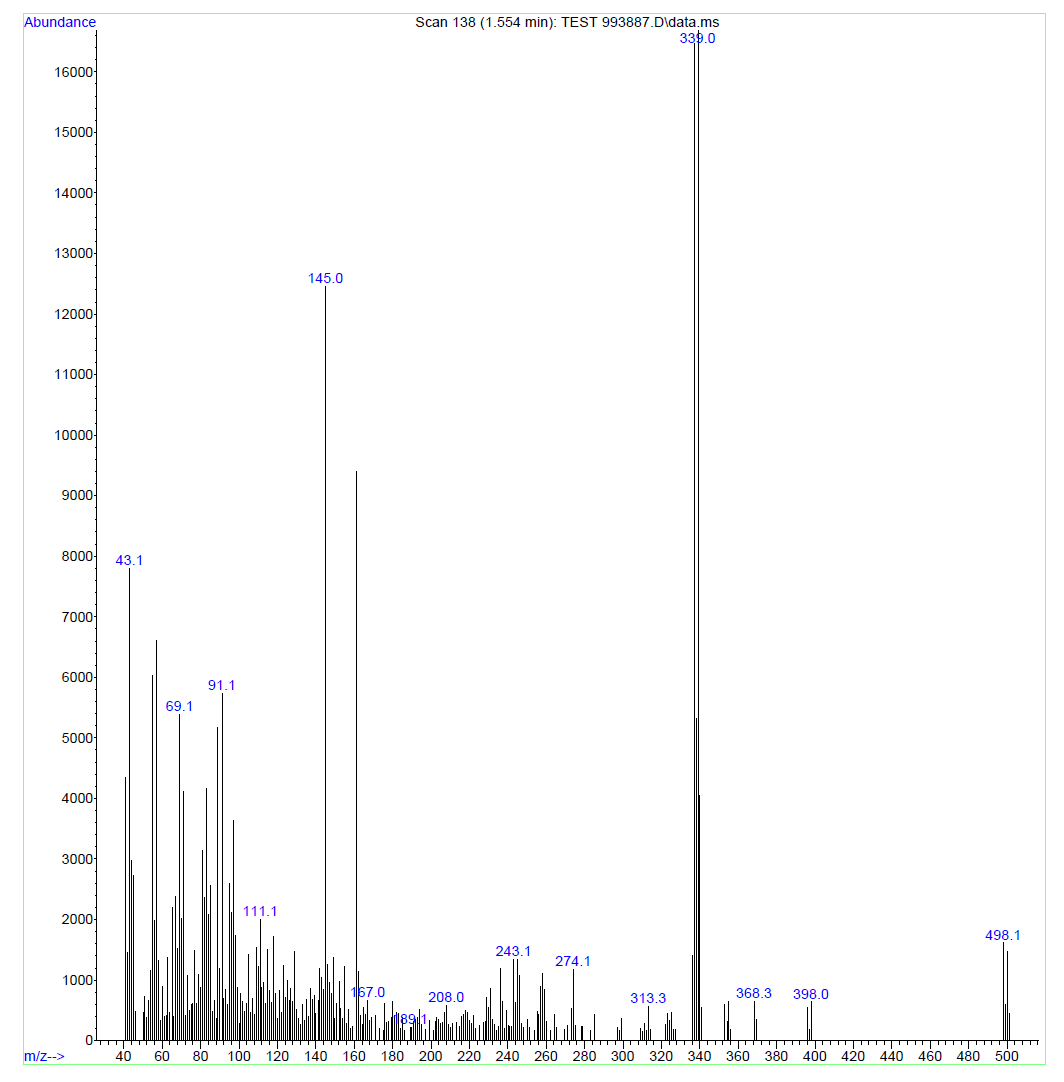


**Fig. S42.** Mass spectra of N'-((3-(4-Bromophenyl)-1-p-tolyl-1H-pyrazol-4-yl)methylene)benzofuran-2-carbohydrazide (8n)


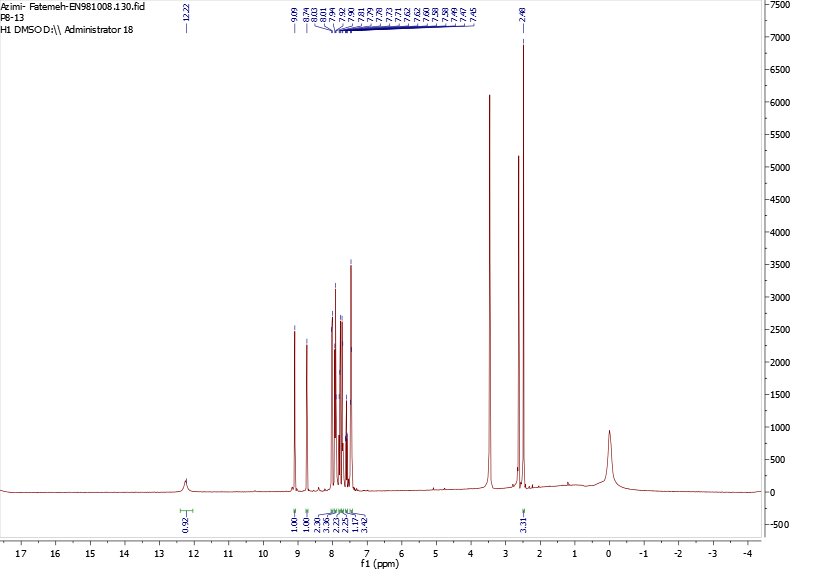


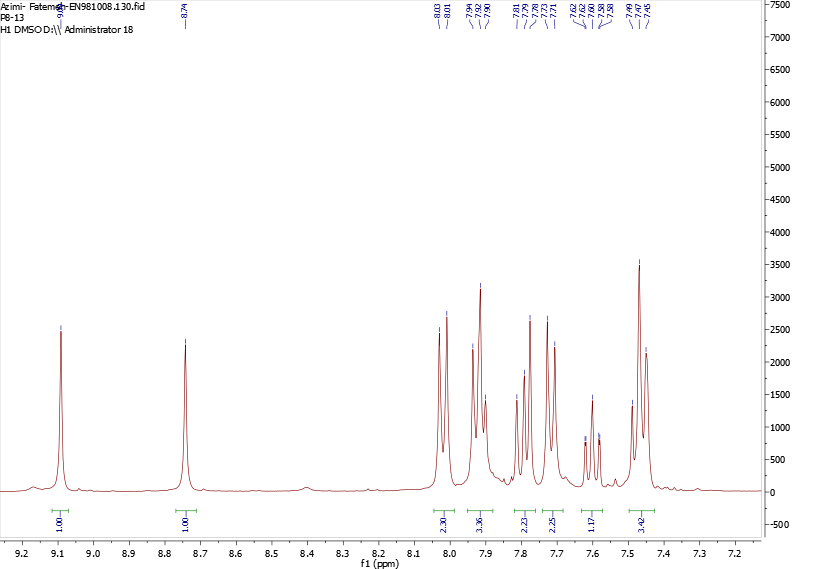


**Fig. S43.** ^1^H NMR spectra of N'-((3-(4-Chlorophenyl)-1-p-tolyl-1H-pyrazol-4-yl)methylene)benzofuran-2-carbohydrazide (8o)


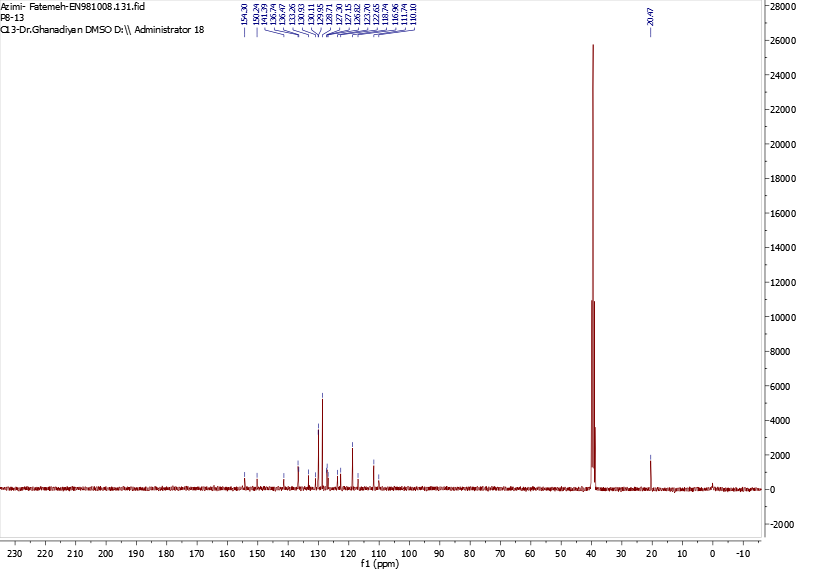


**Fig. S44.** ^13^C NMR spectra of N'-((3-(4-Chlorophenyl)-1-p-tolyl-1H-pyrazol-4-yl)methylene)benzofuran-2-carbohydrazide (8o)


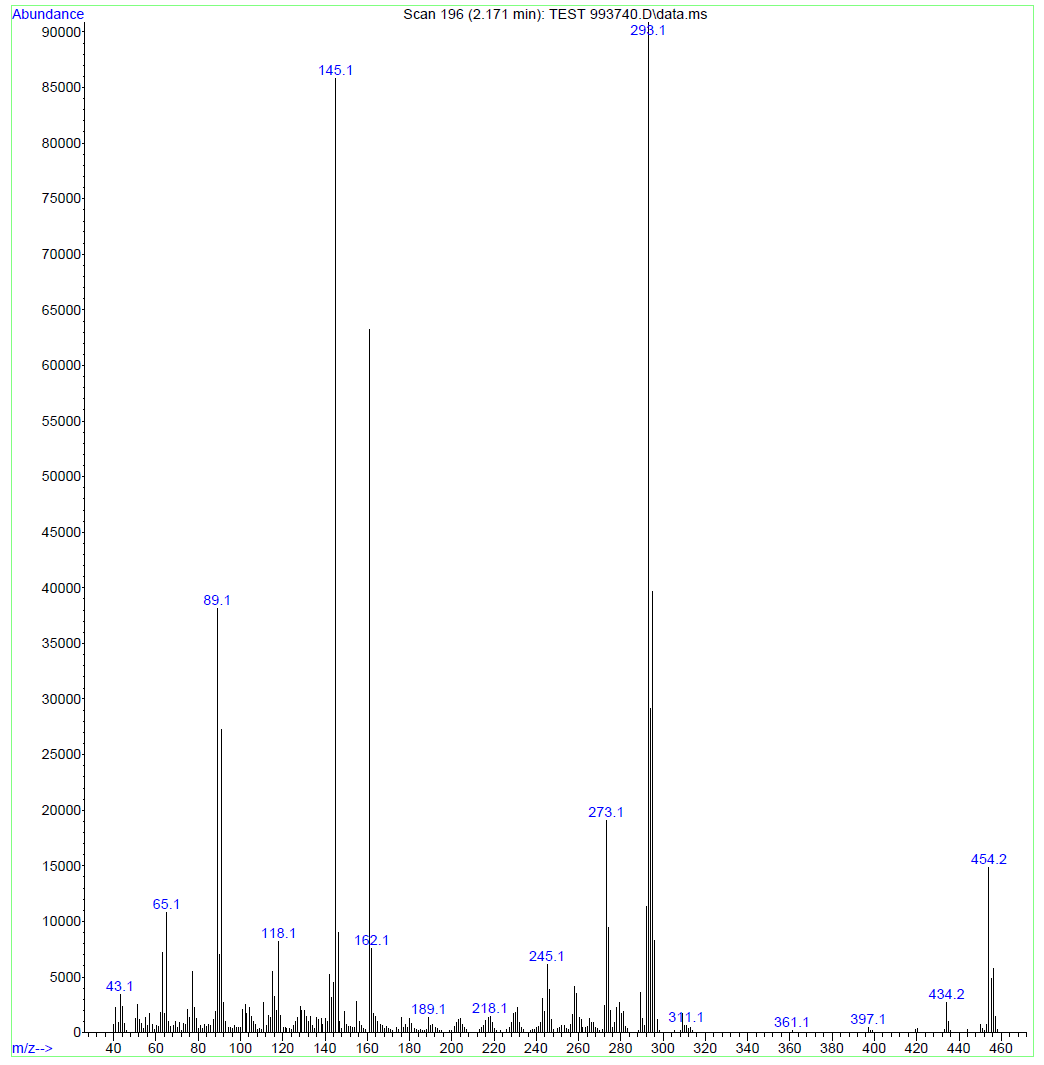


**Fig. S45.** Mass spectra of N'-((3-(4-Chlorophenyl)-1-p-tolyl-1H-pyrazol-4-yl)methylene)benzofuran-2-carbohydrazide (8o)
